# Adherence interventions and outcomes of tuberculosis treatment: A systematic review and meta-analysis of trials and observational studies

**DOI:** 10.1371/journal.pmed.1002595

**Published:** 2018-07-03

**Authors:** Narges Alipanah, Leah Jarlsberg, Cecily Miller, Nguyen Nhat Linh, Dennis Falzon, Ernesto Jaramillo, Payam Nahid

**Affiliations:** 1 University of California San Francisco, Division of Pulmonary and Critical Care Medicine, Zuckerberg San Francisco General, San Francisco, California, United States of America; 2 Santa Clara Valley Medical Center, Department of Internal Medicine, San Jose, California, United States of America; 3 Global TB Programme, World Health Organization, Geneva, Switzerland; Harvard School of Public Health, UNITED STATES

## Abstract

**Background:**

Incomplete adherence to tuberculosis (TB) treatment increases the risk of delayed culture conversion with continued transmission in the community, as well as treatment failure, relapse, and development or amplification of drug resistance. We conducted a systematic review and meta-analysis of adherence interventions, including directly observed therapy (DOT), to determine which approaches lead to improved TB treatment outcomes.

**Methods and findings:**

We systematically reviewed Medline as well as the references of published review articles for relevant studies of adherence to multidrug treatment of both drug-susceptible and drug-resistant TB through February 3, 2018. We included randomized controlled trials (RCTs) as well as prospective and retrospective cohort studies (CSs) with an internal or external control group that evaluated any adherence intervention and conducted a meta-analysis of their impact on TB treatment outcomes. Our search identified 7,729 articles, of which 129 met the inclusion criteria for quantitative analysis. Seven adherence categories were identified, including DOT offered by different providers and at various locations, reminders and tracers, incentives and enablers, patient education, digital technologies (short message services [SMSs] via mobile phones and video-observed therapy [VOT]), staff education, and combinations of these interventions. When compared with DOT alone, self-administered therapy (SAT) was associated with lower rates of treatment success (CS: risk ratio [RR] 0.81, 95% CI 0.73–0.89; RCT: RR 0.94, 95% CI 0.89–0.98), adherence (CS: RR 0.83, 95% CI 0.75–0.93), and sputum smear conversion (RCT: RR 0.92, 95% CI 0.87–0.98) as well as higher rates of development of drug resistance (CS: RR 4.19, 95% CI 2.34–7.49). When compared to DOT provided by healthcare providers, DOT provided by family members was associated with a lower rate of adherence (CS: RR 0.86, 95% CI 0.79–0.94). DOT delivery in the community versus at the clinic was associated with a higher rate of treatment success (CS: RR 1.08, 95% CI 1.01–1.15) and sputum conversion at the end of two months (CS: RR 1.05, 95% CI 1.02–1.08) as well as lower rates of treatment failure (CS: RR 0.56, 95% CI 0.33–0.95) and loss to follow-up (CS: RR 0.63, 95% CI 0.40–0.98). Medication monitors improved adherence and treatment success and VOT was comparable with DOT. SMS reminders led to a higher treatment completion rate in one RCT and were associated with higher rates of cure and sputum conversion when used in combination with medication monitors. TB treatment outcomes improved when patient education, healthcare provider education, incentives and enablers, psychological interventions, reminders and tracers, or mobile digital technologies were employed. Our findings are limited by the heterogeneity of the included studies and lack of standardized research methodology on adherence interventions.

**Conclusion:**

TB treatment outcomes are improved with the use of adherence interventions, such as patient education and counseling, incentives and enablers, psychological interventions, reminders and tracers, and digital health technologies. Trained healthcare providers as well as community delivery provides patient-centered DOT options that both enhance adherence and improve treatment outcomes as compared to unsupervised, SAT alone.

## Abstract 2

In this systematic review and meta-analysis, Payam Nahid and colleagues investigate results from trials and observational studies to identify methods for encouraging patients to complete tuberculosis treatment that are associated with improved TB outcomes.

## Author summary

**Why was this study done?:**

- Tuberculosis remains a global problem and is the leading cause of death from a single infectious pathogen.
- Tuberculosis treatment involves taking multiple medications daily for months to years, depending on the level of drug resistance, and failure to complete therapy as prescribed can lead to poor outcomes, including increased risk of failure, disease relapse, continued transmission, development of drug resistance, and death.
- Public health programs and researchers have studied different ways of encouraging patients to take tuberculosis medications as prescribed in order to improve the likelihood of curing patients, but it is unclear which interventions are most effective.

**What did the researchers do and find?:**

- We systematically reviewed all published articles on this topic and found 129 research studies on different methods to encourage patients to complete tuberculosis treatment.
- We found that providing patients with more support during their treatment increases the number of patients who successfully complete their treatment and reduces the number of patients who develop drug-resistant tuberculosis.
- Some of these support measures include different methods of reminding patients to take their medication doses, such as having a healthcare worker observe them, sending them reminder text messages, or helping them with financial resources to make it to their clinic appointments.

**What do these findings mean?:**

- In addition to prescribing tuberculosis medications to patients with active tuberculosis, public health programs should include resources to help patients overcome individual challenges to completing treatment.
- Using financial incentives, tools such as text messaging, and the use of healthcare workers to observe patients taking their medications at home or in the community can help patients overcome some of the barriers to treatment completion.
- Combining multiple different types of patient-centered support interventions tailored to an individual’s needs and values can improve tuberculosis control efforts.

## Introduction

Adherence to treatment is challenging, given the complexity, modest tolerability, and long duration of treatment regimens currently available for both drug-susceptible and -resistant tuberculosis (TB). In turn, low adherence increases the risk of poor outcomes, including treatment failure, relapse, and development or amplification of drug resistance [1–6]. Public health programs have used a variety of strategies to improve adherence at the health system level via financial incentives or enablers to offset the cost of accessing treatment, improving coordination and logistics around TB treatment delivery, and training healthcare providers. Other strategies tackle barriers to completing TB treatment by addressing knowledge gaps, attitudes, and behaviors surrounding adherence to TB treatment [7–11]. One of the most commonly used adherence interventions is directly observed therapy (DOT), in which a health worker, family member, or community member observes the patient taking TB medications [12]. In recent years, video-observed therapy (VOT) has gained attention as an alternative way of delivering DOT [13,14]. Other interventions aimed at supporting adherence through DOT include incentives, which are material or financial rewards provided to those adhering treatment [15], and enablers, which are interventions that allow patients to overcome economic constraints associated with DOT, such as absence from work or the direct and indirect patient costs of accessing TB treatment. Other interventions focus on providing education on TB, its treatment, and prevention to help patients make informed decisions and the healthcare team to deliver patient-centered care [16]. Reminder systems and patient tracers are targeted at assisting patients to keep appointments and to take action when patients miss appointments [17]. These interventions include reminder letters, phone calls, home visits, and, more recently, short message service (SMS) technology as well as electronic pill boxes. Psychological interventions aim to support via psychological or emotional counseling or a social network of peers undergoing TB treatment as a means of improving adherence to TB treatment [18,19]. Given the significant losses patients and the health system incur as a result of poor TB treatment outcomes, identifying those interventions that are most likely to improve adherence and outcomes, especially in resource-limited settings, is crucial.

The first pillar of the End TB Strategy of the World Health Organization (WHO)—Integrated, Patient Centered Care and Prevention—calls for “treatment of all people with tuberculosis including drug-resistant tuberculosis; and patient support” [20]. In 2015, WHO commissioned a series of systematic reviews and meta-analyses ahead of a Guideline Development Group meeting tasked with the revision of its TB treatment guidelines in accordance with the Grading of Recommendations Assessment, Development, and Evaluation (GRADE) approach [21–23]. Until then, no evidence-based recommendations on TB treatment adherence and delivery existed. In order to inform this process, we conducted a systematic review and meta-analysis of studies of adherence interventions on drug-susceptible and -resistant TB treatment outcomes. Our goal was to identify any adherence interventions associated with improvement in TB treatment outcomes.

## Methods

### Search strategy and selection criteria

This study has been designed and reported according to the Preferred Reporting Items for Systematic Reviews and Meta-Analysis (PRISMA, S1 Text). The full protocol for this study is available in the supplementary material (S2 Text).

We included all randomized controlled trials (RCTs) as well as prospective and retrospective cohort studies (CSs). The population of interest included all adults or children in any setting undergoing active TB treatment. This included patients with pulmonary, extrapulmonary, smear-positive or -negative, and drug-susceptible and -resistant TB as well as patients with HIV coinfection. To be included, studies must have had an intervention targeted to increase adherence to TB treatment and an internal or historical control group (Table 1). We excluded articles on patients with only latent TB infection. We also excluded studies that compared DOT delivered in a hospital versus clinic setting because of a separate focused systematic review being conducted at the time of this review.

**Table 1:** PICO question breakdown for adherence interventions in TB treatment.

Population	Intervention	Comparator	Outcome
Patients on treatment for drug-sensitive TB Patients on MDR-TB treatment Children (0–14 y) and adults TB patients infected with HIV and not infected with HIV	Any intervention to promote treatment adherence Supervising treatment (DOT, VOT) Measures to improve treatment adherence (e.g., medication monitors and/or SMS or phone call reminders) Social support (educational, psychological, material) Combinations of the above interventions	Routine practice*	Adherence to treatment (or treatment interruption because of nonadherence) Conventional TB treatment outcomes: cured/completed, failure, relapse, survival/death Adverse reactions from TB drugs (severity, type, organ class)

*Routine practice refers to regular TB drugs pickup and consultations with a physician or other healthcare workers being available when necessary, TB treatment being free of charge; and essential information/health education in relation to TB treatment being provided [24].

Abbreviations: DOT, directly observed therapy; MDR-TB, multidrug-resistant tuberculosis; PICO, population, intervention, comparison, outcome; SMS, short message service; TB, tuberculosis; VOT, video-observed therapy.

The following were the primary outcomes of interest as defined by WHO [25]: Cure, a pulmonary TB patient with bacteriologically confirmed TB at the beginning of treatment who was smear or culture negative in the last month of treatment and on at least one previous occasion; Treatment completion, a TB patient who completed treatment without evidence of failure but with no record to show that sputum smear or culture results in the last month of treatment and on at least one previous occasion were negative, either because tests were not done or because results are unavailable; Treatment success, the sum of “cure” and “treatment completion”; Treatment failure, a TB patient whose sputum smear or culture is positive at month 5 or later during treatment; Death, a TB patient who dies for any reason before starting or during the course of treatment; Loss to follow-up, a TB patient who did not start treatment or whose treatment was interrupted for two consecutive months or more; Relapse, a patient with a bacteriologically positive sputum smear or culture after the completion of any of the study TB regimens; Adherence, defined using parent study definitions such as being lost to follow-up (or default), isoniazid (INH) urine test, appointment keeping, etc.; and Development of resistance, identification of new drug resistance in a subsequent isolate otherwise matched with the baseline isolate. In post hoc analyses, given the number of articles that defined adherence as having taken more than 80%–90% of treatment doses, and for ease of comparison between studies, we chose to use this definition for the outcome of adherence when available.

We searched the literature using Medline with two search strategies, one to include all types of adherence interventions and the second targeted at SMS/VOT through February 3, 2018. The complete search strategy is available in S1 and S2 Tables. We also reviewed references of relevant articles and systematic reviews and contacted experts in the field for unpublished studies. We included all studies in the English language regardless of publication status or date. However, two foreign language articles were included, as data from them were previously abstracted by a different systematic review. Titles and abstracts were reviewed by one investigator (NA) and full manuscript content reviewed by multiple investigators (NA, PN, LJ, CM). Ethics approval was not required for this study, as all information was abstracted from published literature without access to any individual and/or identifiable data.

### Data extraction and variable definitions

Using a standard data abstraction sheet, the following data were recorded from articles that met our inclusion criteria: patient selection, type of TB (pulmonary or extrapulmonary), HIV coinfection, treatment outcomes, method of blinding and randomization, results of drug-susceptibility testing, acid-fast bacilli (AFB) smear and culture results, mode of supervision of therapy, type of adherence intervention, and study results for the outcomes of interest (adjusted or unadjusted risk ratios [RRs], risk differences [RDs], raw data). We chose to use unadjusted RRs preferentially if these data were available. When available, data were gathered from both per protocol and intention-to-treat analyses. The quality of the studies was assessed using the Cochrane Risk of Bias tool for RCTs, and the Newcastle-Ottawa Scale was used for observational studies [26,27].

We grouped the adherence interventions identified across studies into the following categories: (1) DOT referred to the act of observing a patient swallow medications, and self-administered therapy (SAT) was defined as the patient taking each medication dose without supervision; (2) education and counseling interventions were those aimed at providing adequate knowledge and ensuring patient understanding of the disease process and risks and benefits associated with treatment adherence; (3) incentives were interventions to promote treatment adherence through a financial or material reward and enablers were interventions that allowed patients to overcome barriers to treatment adherence (cost, distance, availability); (4) reminders included any intervention made prior to the patient taking medications or attending appointments to serve as a reminder and tracers involved contacts made after a patient had failed adherence in order to improve subsequent adherence to treatment; (5) psychological interventions aimed to provide emotional or psychological support aimed at reducing stigma and increasing treatment adherence; (6) digital health interventions included any of the above categories implemented via mobile electronic devices (SMS, VOT, medication monitors); and lastly, (7) mixed interventions included a combination of the aforementioned interventions to address barriers to adherence based on patient-specific needs and values. Thus, the term “patient-centered DOT” or “enhanced DOT” was used to designate any study using adherence interventions spanning the multiple categories mentioned above in conjunction with DOT. With respect to DOT provider type, a lay provider was defined as an untrained volunteer, including family members or other patient-designated person. A trained health worker included any health worker, community member, or volunteer who had received any form of formal training for DOT. For the categories of patient education and counseling, staff education, incentives and enablers, reminders and tracers, and psychological interventions, studies included in the meta-analysis had to have employed the same supervision modality (either DOT or SAT) for both the intervention and control arms.

All estimates of effect for dichotomous outcomes were reported as RRs with 95% confidence intervals. When two or more studies were available on a particular outcome, random effects meta-analysis was performed to obtain a pooled estimate of treatment effect and pooled RR between the intervention and control arms. Heterogeneity was assessed visually using forest plots and statistically using the χ^2^ and I^2^ tests. If more than 10 studies were available for a particular comparison, we used funnel plots to determine publication bias. All analyses were conducted in RevMan5 [28]. All figures were generated using RevMan and GraphPad Prism 7.0 [29]. If a study reported no events in the intervention or control group for an outcome of interest, the RD was calculated.

## Results

**Fig 1:**
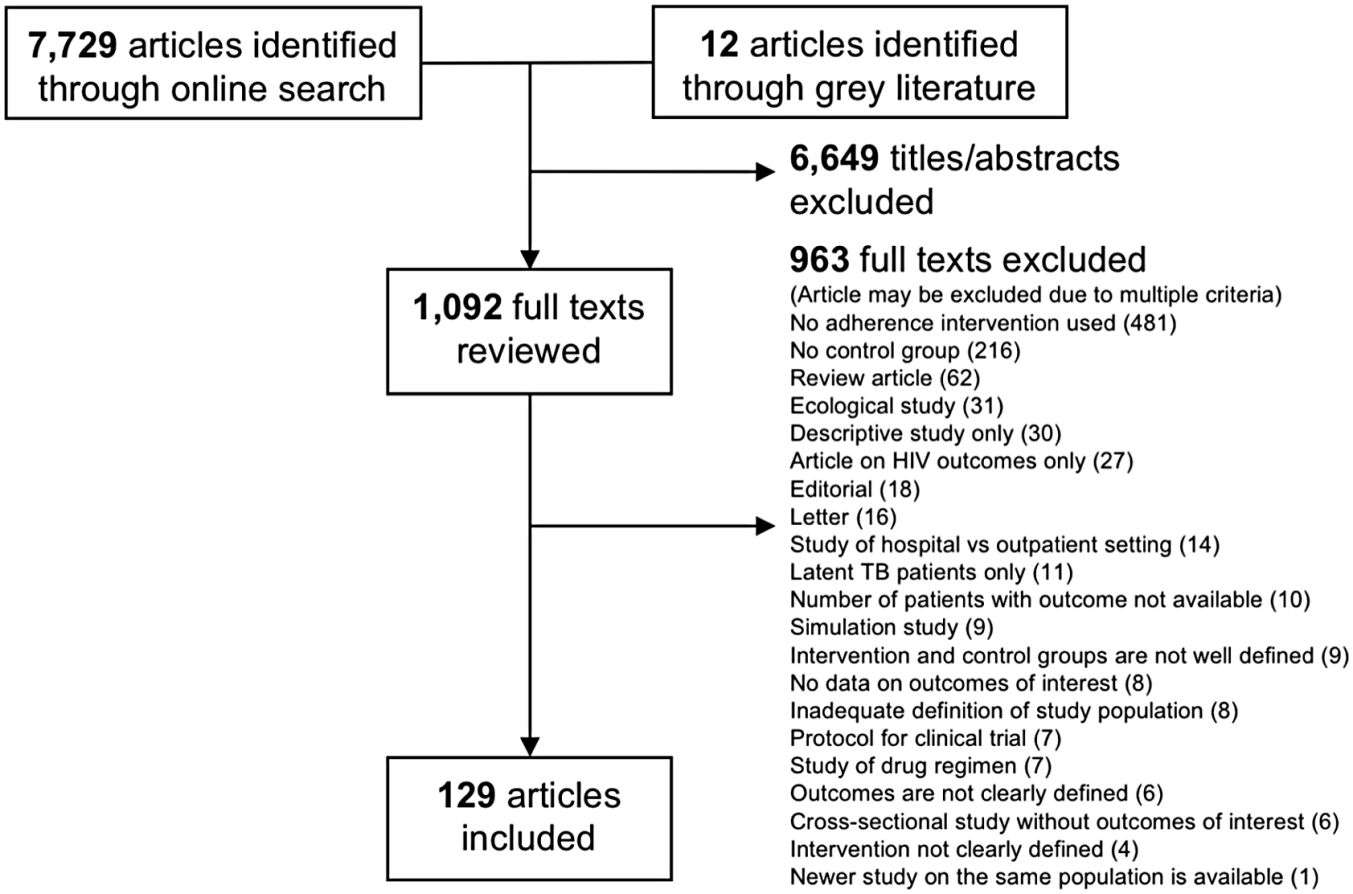
PRISMA summary. TB, tuberculosis.

Title and abstract literature review yielded 7,729 articles, of which 1,092 met the inclusion criteria for full text review (Fig 1). References of 32 systematic reviews found through our online search were also reviewed for relevant articles. A final 129 articles met the inclusion criteria for quantitative analysis. Characteristics of included studies as well as quality assessments are summarized in S3 Table, S1 and S2 Figs. The overall quality of included studies varied significantly amongst CSs. The quality of RCTs was limited predominantly by lack of blinding, given the nature of adherence interventions. Below is a brief summary of findings by each type of adherence intervention (Table 2). When available, data from RCTs and CSs are listed in parentheses. Some of the analyses contained significant heterogeneity, as measured by I^2^, which has been denoted on their corresponding figures.

**Table 2:** Summary of the effects of different types of adherence interventions on TB treatment outcomes.

Outcomes	*SAT versus any DOT* *(No. of studies)*	*SAT versus any DOT-HIV/TB* *(No. of studies)*	*Family/community DOT versus HCW DOT* *(No. of studies)*	*Lay provider DOT versus HCW DOT* *(No. of studies)*	*Home DOT versus clinic DOT* *(No. of studies)*	*Community DOT versus clinic DOT* *(No. of studies)*	*Home DOT versus community DOT* *(No. of studies)*	*Patient education*^2^ *(No. of studies)*	*Incentives/enablers*^2^ *(No. of studies)*	*Reminders/tracers*^2^ *(No. of studies)*	*Patient-centered DOT versus SAT* *(No. of studies)*	*Patient-centered DOT versus DOT* *(No. of studies)*	*Patient-centered SAT versus SAT* *(No. of studies)*	*Psychological interventions*^2^ *(No. of studies)*	*Staff education*^2^ *(No. of studies)*	*Phone reminders*^2^ *(No. of studies)*	*VOT versus DOT* *(No. of studies)*
*Mortality—CSs*	Ø (23)	↑ (4)	Ø (3)	Ø (3)	↑ (8)	Ø (6)	Ø (2)	--	↓ (3)	Ø (3)	Ø (4)	Ø (4)	--	Ø (1)	--	Ø (2)	Ø (1)
*Mortality—RCTs*	Ø (4)	--	--	--	--	Ø (2)	Ø (1)	Ø (2)	Ø (2)	Ø (3)	Ø (1)	↓ (2)	--	--	Ø (2)	Ø (1)	--
*Success—CSs*	↓ (4)	↓ (3)	Ø (3)	Ø (2)	↑ (3)	↑ (9)	↓ (1)	--	↑ (4)	Ø (2)	↑ (2)	↑ (4)	--	--	↑ (1)	--	--
*Success—RCTs*	↓ (5)	--	--	--	--	Ø (1)	Ø (2)	Ø (2)	↑ (3)	↑ (4)	↑ (1)	↑ (2)	--	--	Ø (3)	Ø (3)	--
*Completion—CSs*	Ø (14)	↓ (1)	Ø (2)	Ø (1)	Ø (5)	Ø (3)	Ø (2)	--	Ø (4)	Ø (1)	↑ (2)	Ø (2)	--	↑ (1)	--	Ø (2)	Ø (2)
*Completion—RCTs*	Ø (5)	--	--	--	--	Ø (1)	--	↑ (1)	↑ (2)	Ø (3)	↑ (1)	Ø (2)	--	↑ (1)	Ø (2)	Ø (1)	--
*Cure—CSs*	↓ (18)	↓ (2)	Ø (3)	Ø (1)	Ø (6)	Ø (6)	Ø (2)	--	↑ (4)	↑ (2)	↑ (2)	Ø (2)	--	--	--	↑ (2)	--
*Cure—RCTs*	Ø (4)	--	--	--	--	Ø (2)	Ø (1)	↑ (1)	Ø (1)	Ø (2)	↑ (2)	↑ (2)	--	Ø (1)	Ø (3)	Ø (1)	--
*Failure—CSs*	Ø (15)	Ø (5)	↑ (3)	Ø (2)	Ø (4)	↓ (6)	Ø (2)	--	Ø (2)	Ø (3)	Ø (2)	Ø (2)	--	--	--	Ø (3)	--
*Failure—RCTs*	Ø (2)	--	--	--	--	Ø (1)	Ø (1)	Ø (1)	↓ (1)	Ø (3)	--	Ø (2)	--	--	Ø (2)	Ø (1)	--
*Loss to follow-up—CSs*	Ø (21)	Ø (3)	Ø (3)	Ø (2)	Ø (7)	↓ (6)	Ø (2)	--	↓ (5)	↓ (4)	Ø (4)	Ø (4)	--	↓ (1)	--	Ø (2)	--
*Loss to follow-up—RCTs*	Ø (4)	--	--	--	--	Ø (2)	Ø (1)	Ø (3)	↓ (1)	Ø (4)	↓ (1)	↓ (3)	--	Ø (1)	Ø (2)	Ø (1)	--
*Relapse—CSs*	Ø (6)	Ø (1)	--	--	--	--	--	--	--	--	Ø (1)	--	--	--	--	--	--
*Relapse—RCTs*	Ø (1)	--	--	--	--	--	--	--	--	--	--	--	--	--	--	--	--
*Adherence—CSs*	↓ (1)	--	↓ (1)	--	↓	--	--	↑ (1)	--	--	--	--	--	--	--	--	--
*Adherence—RCTs*	Ø (1)	--	--	--	--	--	--	↑ (1)	--	↑ (1)	--	↑ (1)	Ø (1)	--	--	--	--
*Smear conversion—CSs*	Ø (2)	--	--	--	↑	↑ (2)	--	--	--	--	--	--	--	--	--	↑ (1)	--
*Smear conversion—RCTs*	↓ (1)	--	--	--	--	Ø (1)	--	--	↑ (1)	↑ (3)	↑ (1)	--	--	--	--	Ø (1)	--
*Development of drug resistance—CSs*	↓ (3)	--	--	--	--	--	--	--	--	↓ (1)	Ø (1)	--	--	--	--	--	--
*Development of drug resistance-RCTs*	--	--	--	--	--	--	--	--	Ø (1)	--	--	--	--	--	--	--	--
Unfavorable outcome*—*CSs*	--	--	--	--	--	↓ (1)	--	--	--	--	--	--	--	--	--	--	--
*Poor adherence*^1^—*RCTs*	--	--	--	--	--	--	--	--	--	--	--	--	--	--	--	Ø (1)	--

Ø No evidence of a difference with the use of intervention versus control.

↑ Statistically significant increased risk of outcome associated with the use of intervention versus control.

↓ Statistically significant decreased risk of outcome associated with the use of intervention versus control.

-- No available data for comparison.

*Unfavorable outcome is defined as combined failure, default, death, or transfer out by the study.

^1^Percentage of patient-months during which >20% of doses were missed.

^2^Comparison of adherence intervention in addition to standard of care versus standard of care alone. Standard of care was DOT or SAT, depending on study setting.

Abbreviations: CS, cohort study; DOT, directly observed therapy; HCW, healthcare worker; RCT, randomized controlled trial; SAT, self-administered therapy.

### SAT versus DOT

Forty-six studies were included in the meta-analysis, seven of which were RCTs [4,30–74]. Six CSs included primarily HIV/TB patients [51,53–55,57,72] and two CSs involved primarily MDR-TB patients [56,72]. DOT was offered daily or intermittently at home, clinic, or in the community. DOT providers ranged from family members to trained lay providers and healthcare providers.

**Fig 2:**
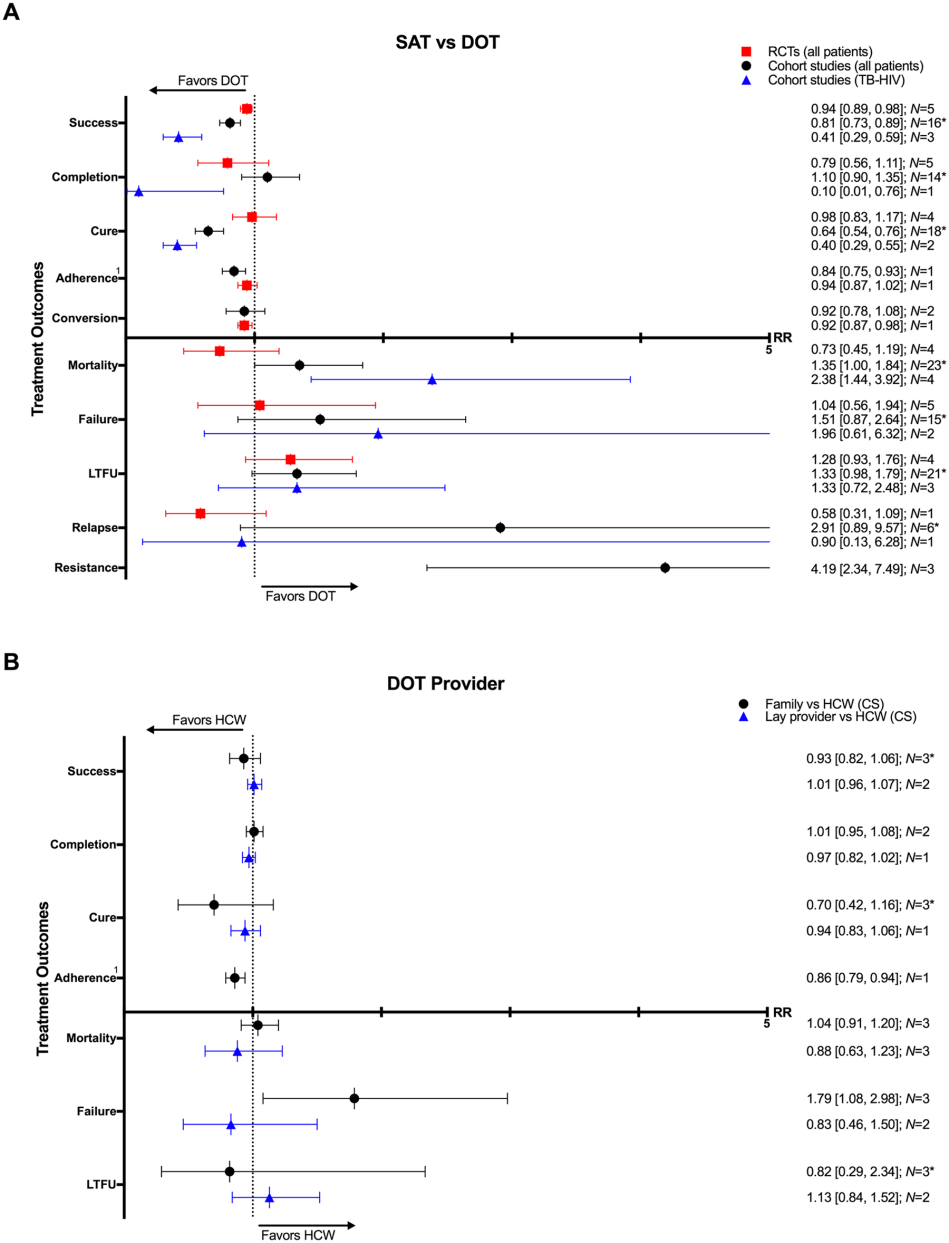
(A) SAT compared with DOT on TB treatment outcomes. (B) Impact of any DOT provided by lay providers, family members, or healthcare workers on TB treatment outcomes. * = significant heterogeneity in the meta-analysis as determined by I^2^ statistic. 1 = depicted is the rate of adherence in one study defined as completing >90% of treatment doses by pill counting in one CS and based on six positive INH urine tests done at random in one RCT. Conversion = sputum conversion to negative at the end of two months (CS) and three months (RCT). *N* = number of studies included within the meta-analysis. Resistance = development of drug resistance. CS, cohort study; DOT, directly observed therapy; HCW, healthcare worker; INH, isoniazid; LTFU, loss to follow-up; RCT, randomized controlled trial; RR, risk ratio; SAT, self-administered therapy; TB, tuberculosis.

**Fig 3:**
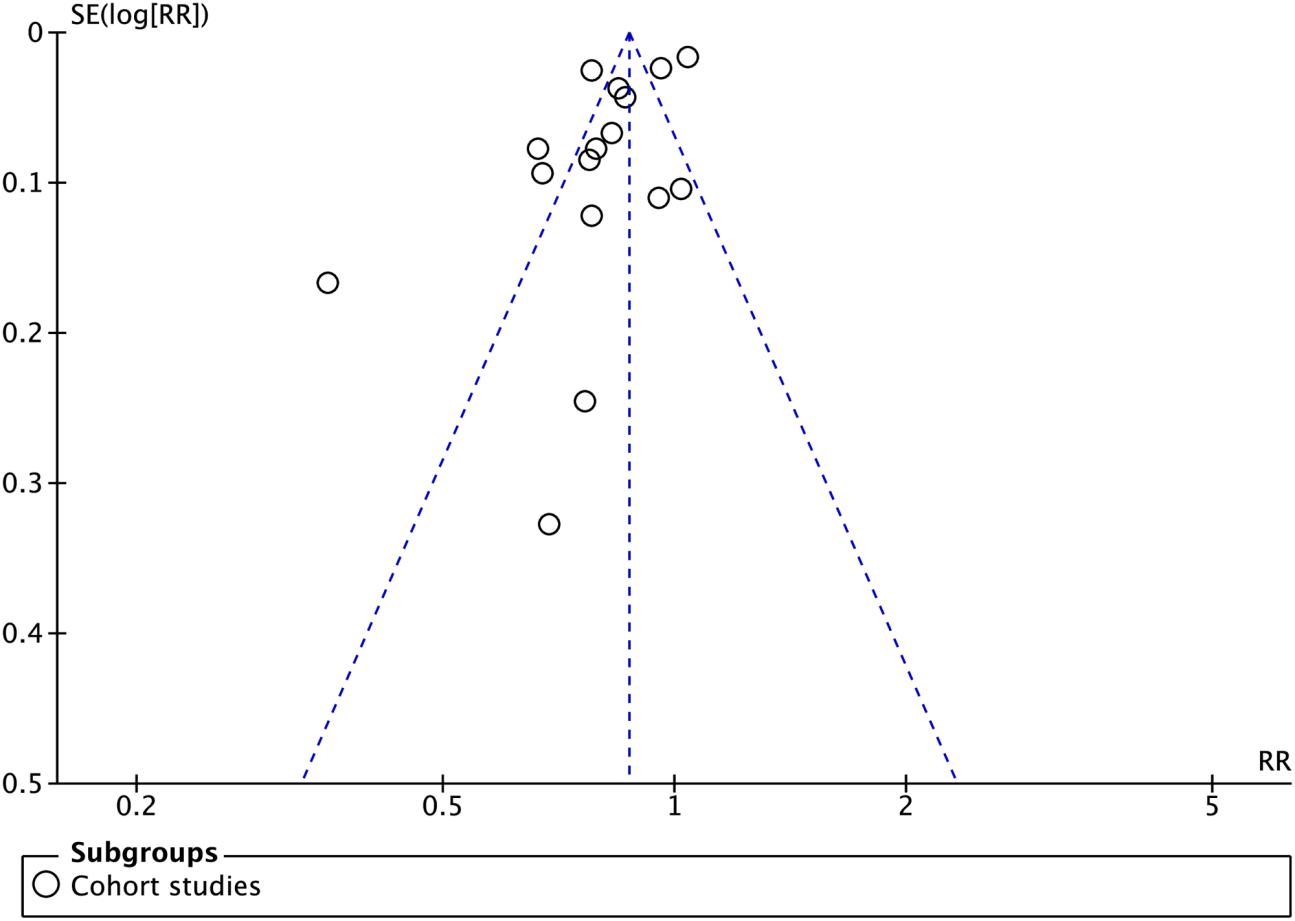
Funnel plot of cohort studies comparing treatment success rates in patients undergoing SAT versus DOT. No funnel plot of RCTs has been included as there were fewer than 10 RCTs. DOT, directly observed therapy; RCT, randomized controlled trial; RR, risk ratio; SAT, self-administered therapy; SE, standard error.

**Fig 4:**
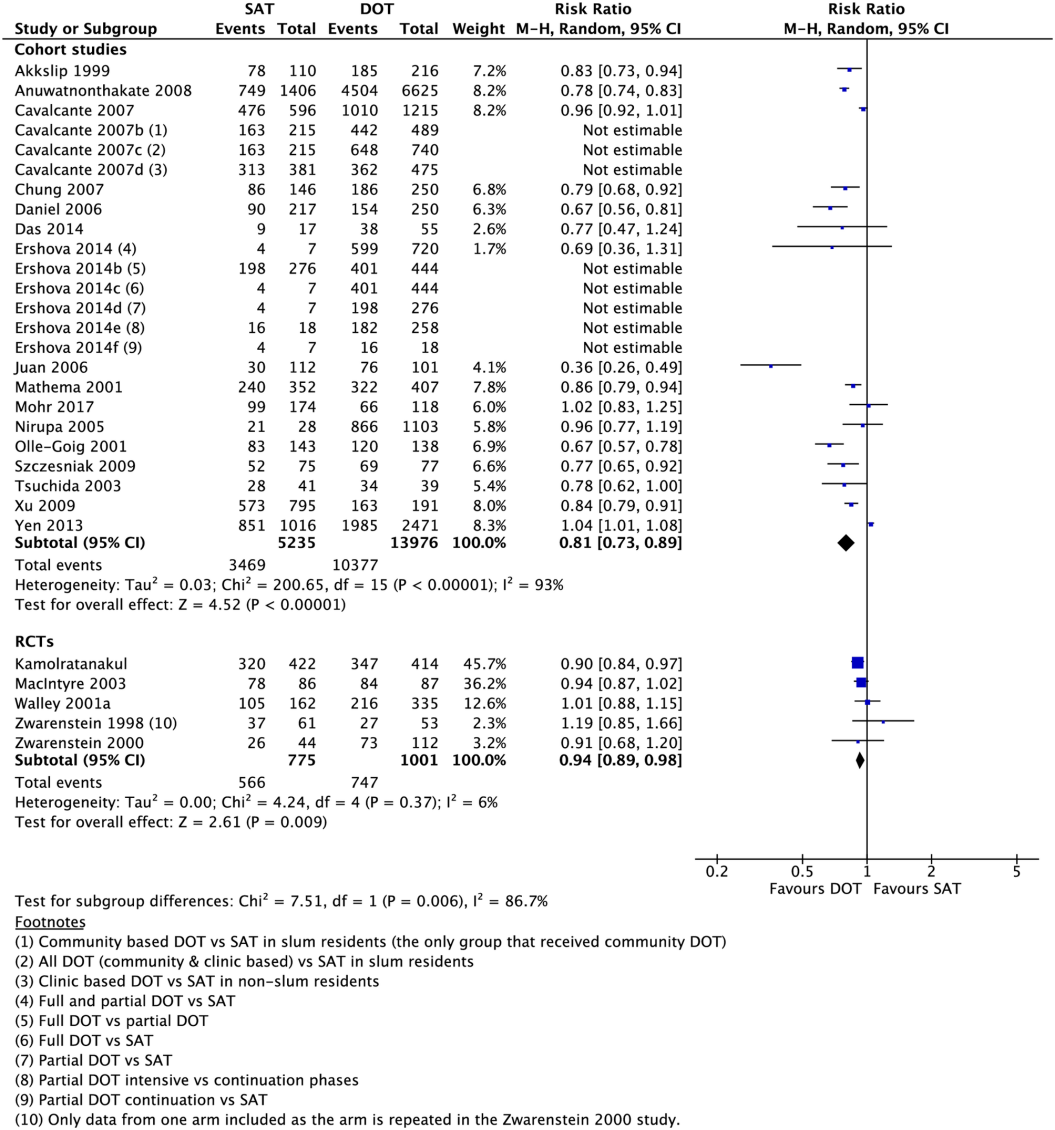
Meta-analysis of treatment success rates in patients undergoing SAT versus DOT. DOT, directly observed therapy; M-H, Mantel-Haenszel; SAT, self-administered therapy.

**Fig 5:**
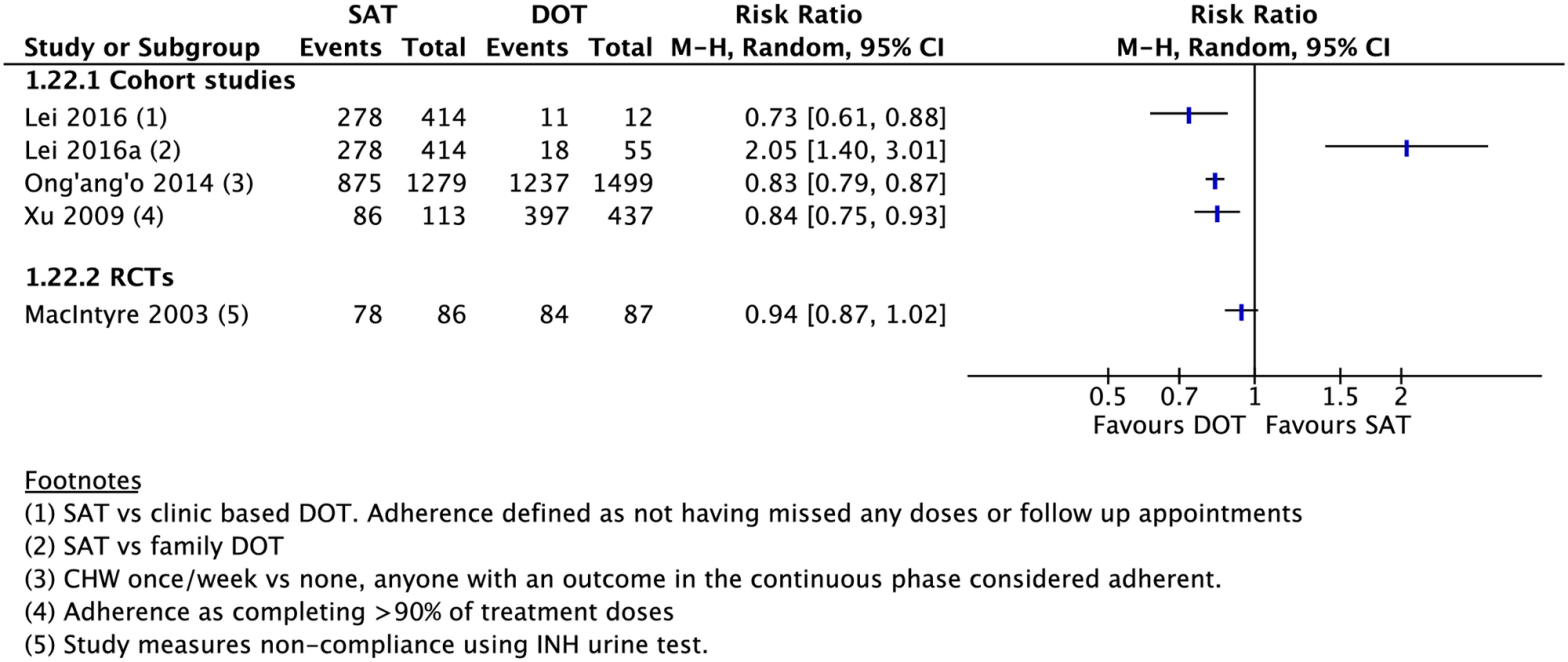
Forest plot of adherence rates in studies comparing patients undergoing SAT versus DOT. CHW, community health worker; DOT, directly observed therapy; INH, isoniazid; M-H, Mantel-Haenszel; SAT, self-administered therapy.

**Fig 6:**
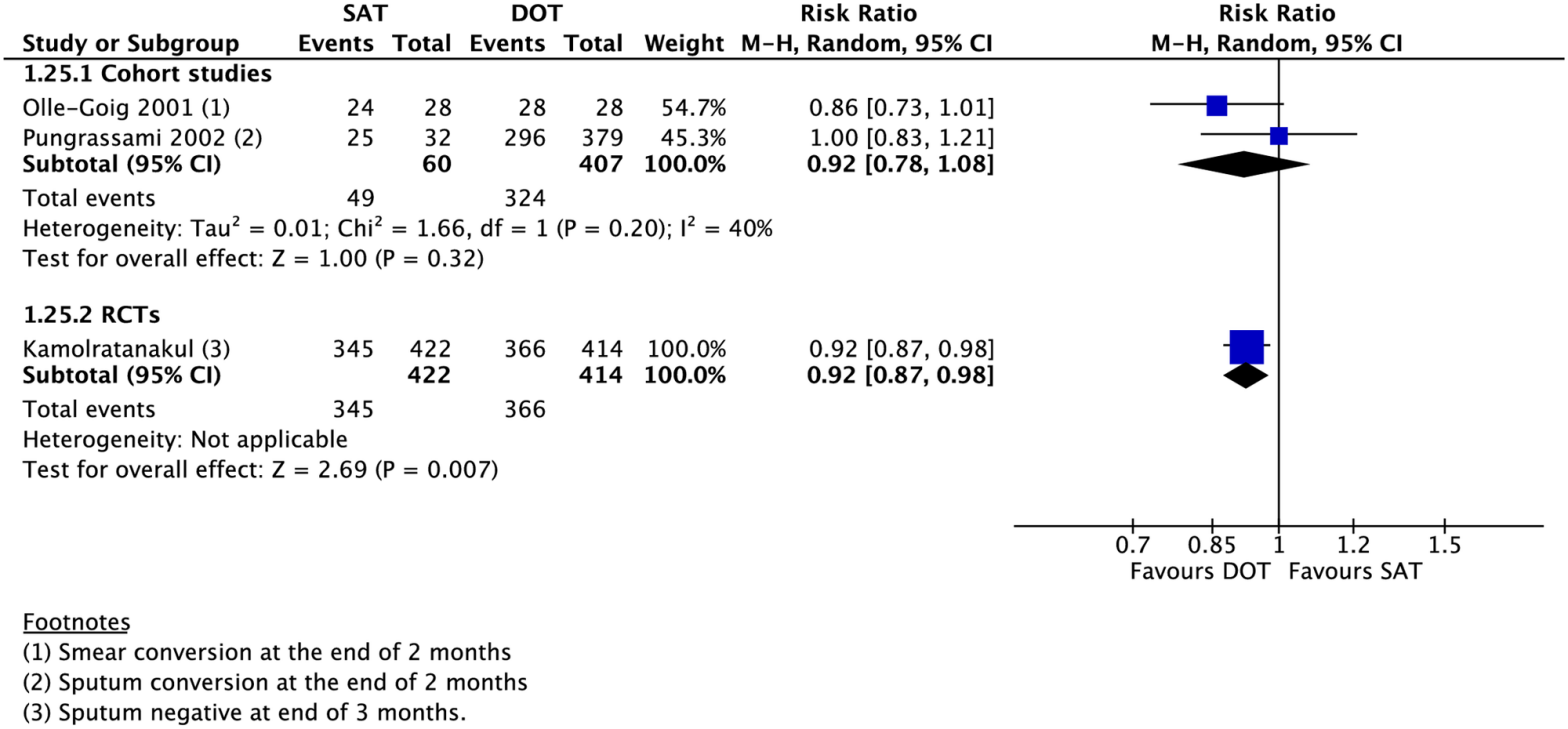
Meta-analysis of rates of sputum conversion in patients undergoing SAT versus DOT. DOT, directly observed therapy; M-H, Mantel-Haenszel; RCT, randomized controlled trial; SAT, self-administered therapy.

Compared to those undergoing any DOT, participants who received SAT had a lower rate of treatment success (16 CSs: RR 0.81, 95% CI 0.73–0.89; 5 RCTs: RR 0.94, 95% CI 0.89–0.98), cure (18 CSs: RR 0.64, 95% CI 0.54–0.76; 4 RCTs: 0.98, 95% CI 0.83–1.17), and adherence (1 CS: RR 0.84, 95% CI 0.75–0.93; 1 RCT: RR 0.94, 95% CI 0.87–1.02) (Figs 2–5). One RCT found a lower rate of smear conversion at the end of two months amongst SAT patients (RR 0.92, 95% CI 0.87–0.98) (Fig 6). There was no significant difference between DOT and SAT amongst all CSs and RCTs for the outcomes of mortality (23 CSs, 4 RCTs), treatment completion (14 CSs, 5 RCTs), treatment failure (15 CSs, 5 RCTs), loss to follow-up (21 CSs, 4 RCTs), relapse (6 CSs, 1 RCT), or development of drug resistance (3 CSs) (S3–S14 Figs).

An assessment of the publication bias (S3, S5, S7, S9 and S11 Figs) showed asymmetry for the outcomes of mortality, treatment success, and loss to follow-up. Notably, a few smaller studies found worse outcomes with SAT, as compared with DOT. The plots reflect the significant heterogeneity between the studies and may also be suggestive of publication bias (i.e., reporting bias in CSs).

**Fig 7:**
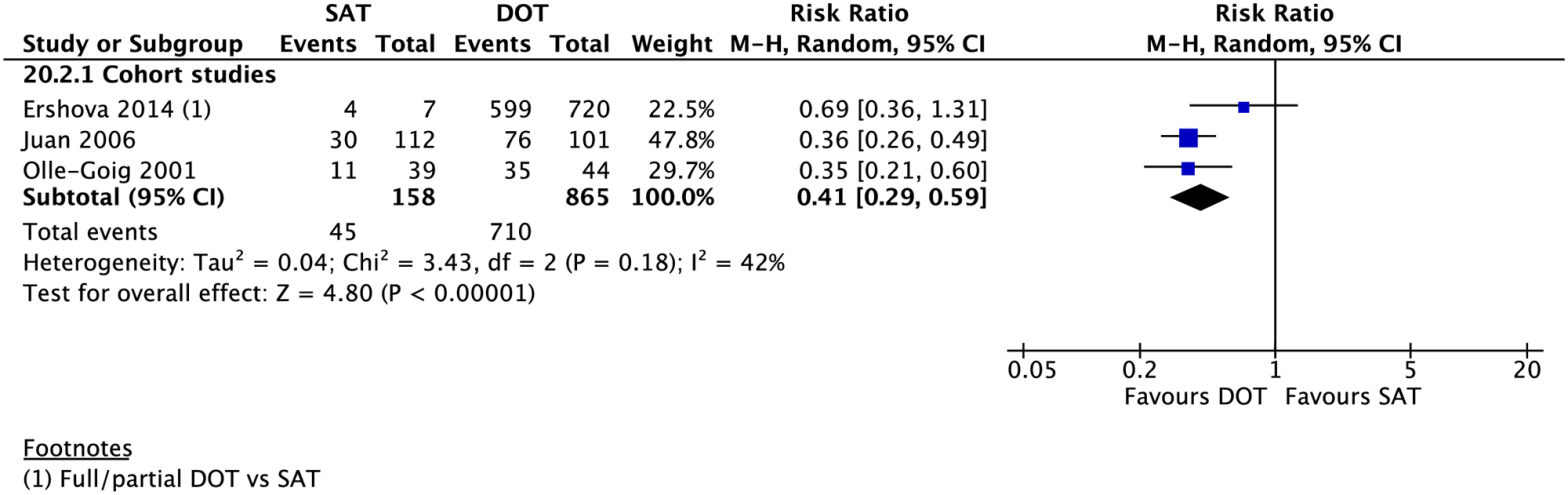
Meta-analysis of treatment success rates in HIV/TB patients undergoing SAT versus DOT. DOT, directly observed therapy; M-H, Mantel-Haenszel; SAT, self-administered therapy; TB, tuberculosis.

In patients with HIV/TB, SAT was associated with lower rates of treatment success (3 CSs: RR 0.41, 95% CI 0.29–0.59), completion (1 CS: RR 0.10, 95% CI 0.01–0.76), and cure (2 CSs: RR 0.40, 95% CI 0.29–0.55) as well as a higher rate of mortality (4 CSs: RR 2.38, 95% CI 1.44–3.92). The difference in the rates of loss to follow-up (3 CSs: RR 1.33, 95% CI 0.72–2.48), treatment failure (2 CSs: RR 1.96, 95% CI 0.61–6.32), and relapse (1 CS: RR 0.90, 95% CI 0.13–6.28) between the two groups (Figs 2, 7 and S16–S20 Figs) was not significant. Although several studies in our meta-analysis included patients with MDR-TB in their cohort, all but two reported aggregate data on treatment outcomes without stratifying by drug-resistant status. Of the 28 MDR-TB patients in one retrospective CS in New York City from 1987–1997 [56], 11 underwent SAT and 17 received DOT. The mortality, treatment completion, and nonadherence rates were not significantly different between the two groups (S21–S24 Figs). A prospective CS in South Africa of rifampicin-resistant patients found no evidence of a difference in rates of mortality, treatment failure, and loss to follow-up between those undergoing DOT for the entire duration of TB treatment and those who only received DOT during the first six months [72].

### DOT provider type

**Fig 8:**
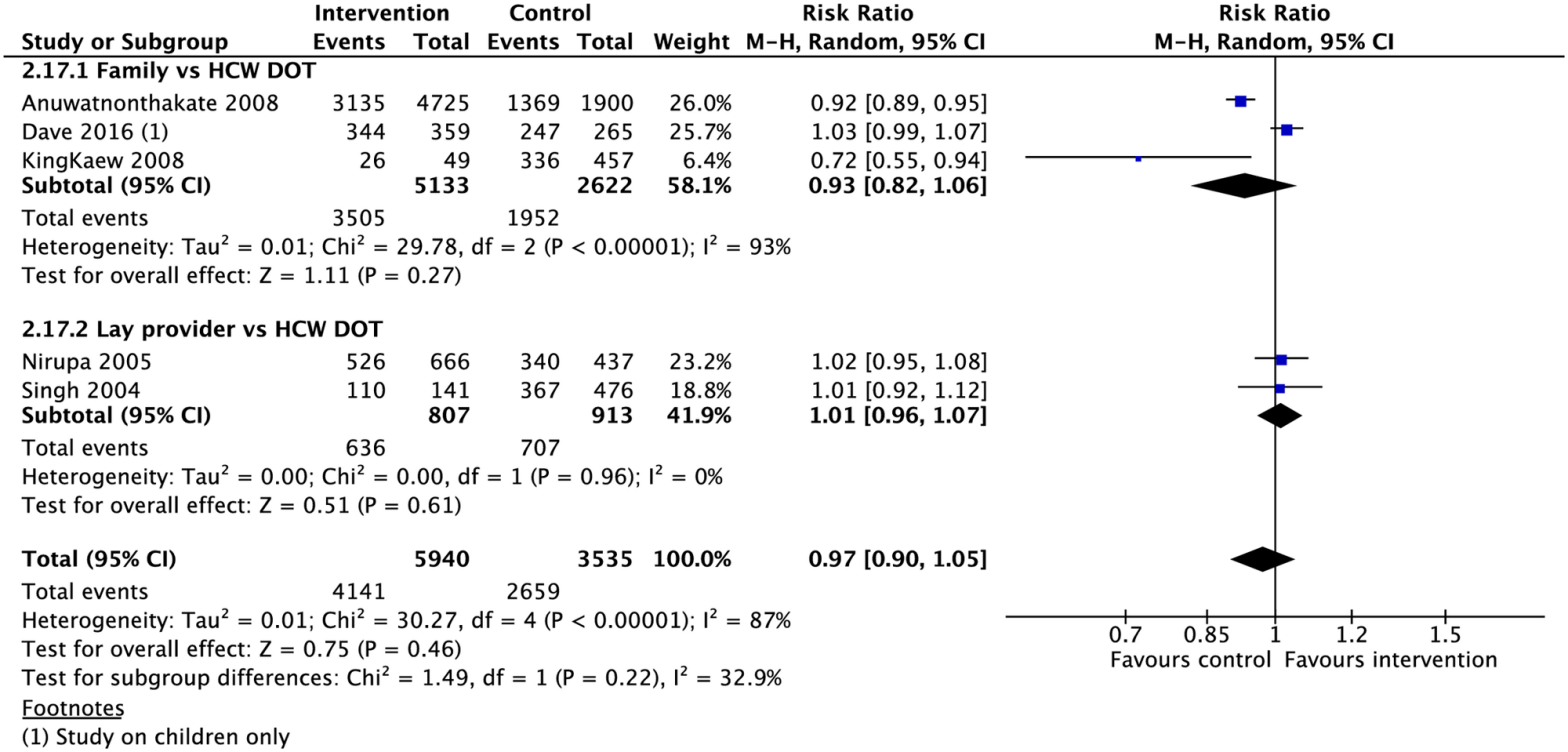
Meta-analysis of treatment success rates in patients receiving DOT by different types of providers. DOT, directly observed therapy; HCW, healthcare worker; M-H, Mantel-Haenszel.

**Fig 9:**
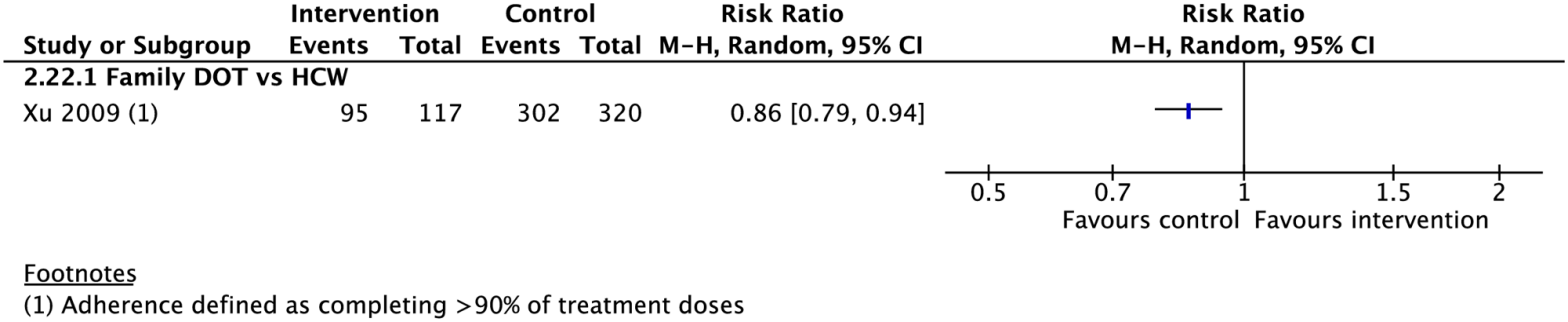
Comparison of adherence rates in patients receiving DOT by a family member versus HCW. DOT, directly observed therapy; HCW, healthcare worker; M-H, Mantel-Haenszel.

Seven CSs compared treatment outcomes in patients who received DOT by family/lay providers or healthcare workers [42,49,63,75–78]. Family member DOT was associated with a lower rate of adherence (1 CS: RR 0.86, 95% CI 0.79–0.94) and higher rate of treatment failure (3 CSs: RR 1.79, 95% CI 1.08–2.98) compared with trained health worker DOT (Fig 2). There was no evidence of a difference in rates of treatment success, completion, cure, mortality, and loss to follow-up between the two groups (Figs 2, 8, 9 and S25–S29 Figs). DOT by lay providers showed no significant difference in outcomes when compared with healthcare worker (HCW) DOT.

### DOT locations

**Fig 10:**
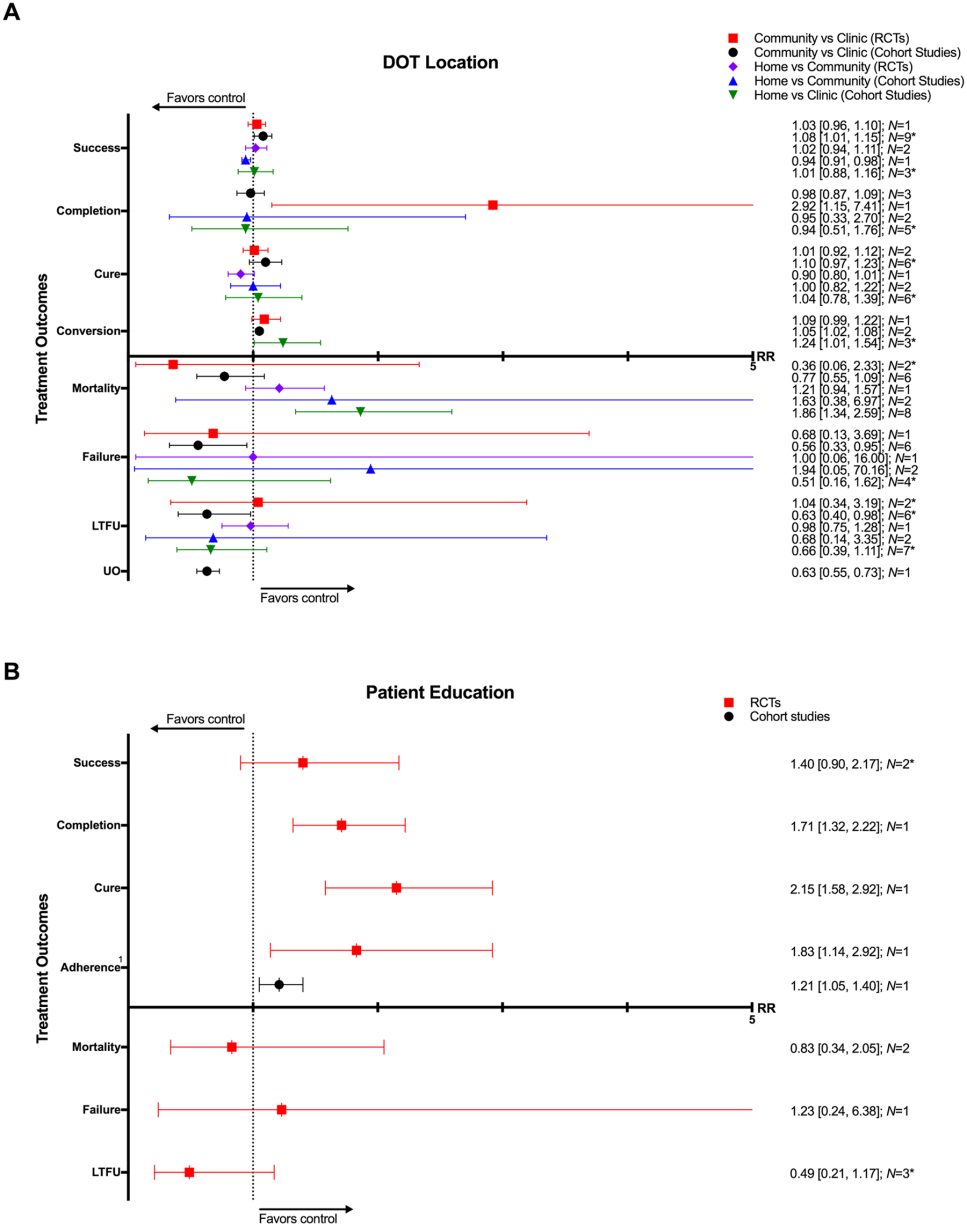
(A) Impact of DOT provided at home, in the community, or in clinic on TB treatment outcomes. (B) Impact of patient education and counseling interventions on TB treatment outcomes. * = significant heterogeneity in the meta-analysis, as determined by I^2^ statistic. 1 = adherence defined as the proportion of patients that took >75% of prescribed doses in one CS and the proportion of patients attending all appointments in one RCT. Conversion = sputum conversion to negative at the end of two months. *N* = number of studies included within the meta-analysis. Composite outcome reported by one study defined as combined failure, default, death, or transfer out. CS, cohort study; DOT, directly observed therapy; LTFU, loss to follow-up; RCT, randomized controlled trial; RR, risk ratio; TB, tuberculosis; UO, unfavorable outcome.

**Fig 11:**
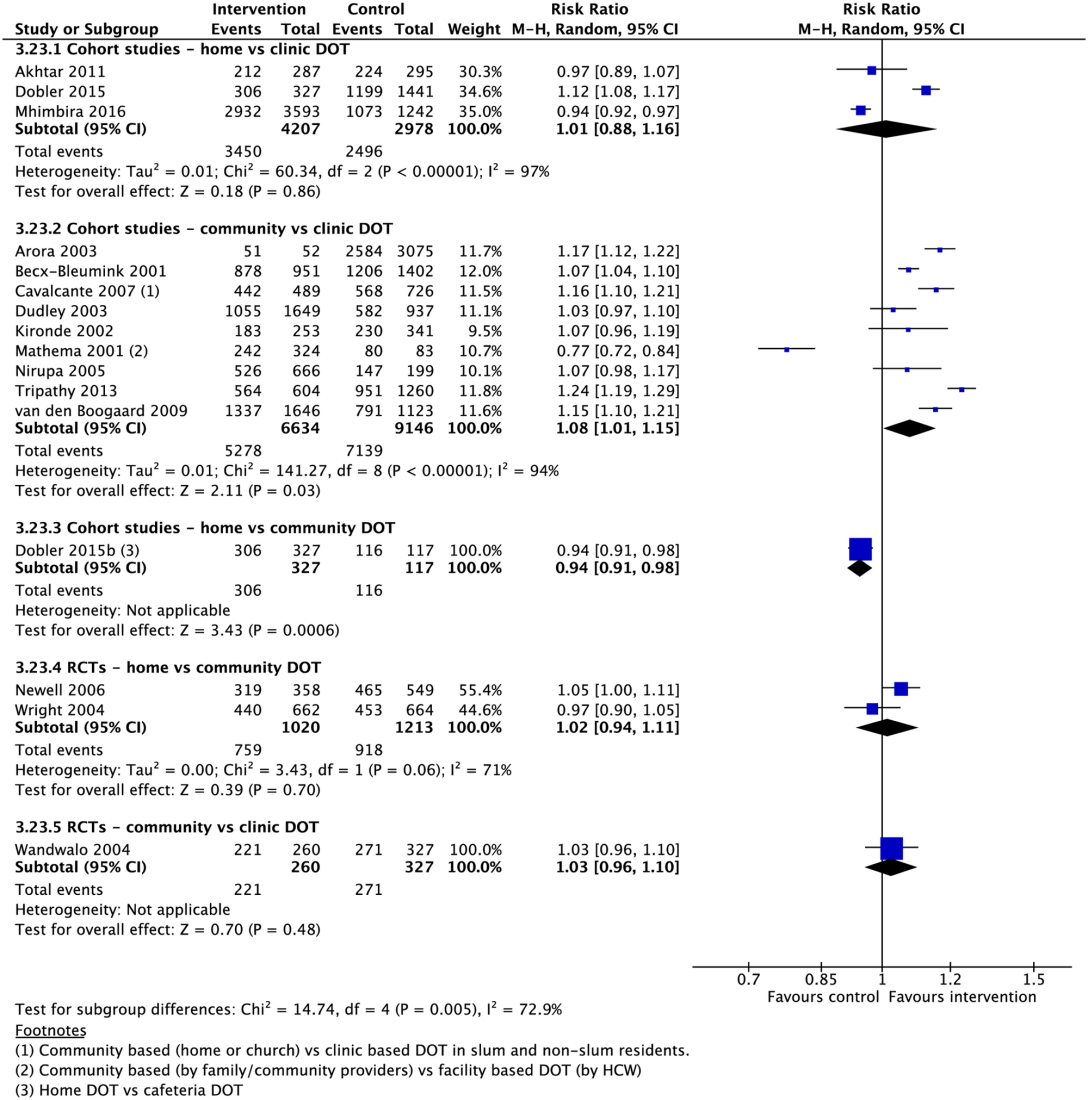
Meta-analysis of treatment success rates in patients receiving DOT in various locations. DOT, directly observed therapy; HCW, healthcare worker; M-H, Mantel-Haenszel; RCT, randomized controlled trial.

**Fig 12:**
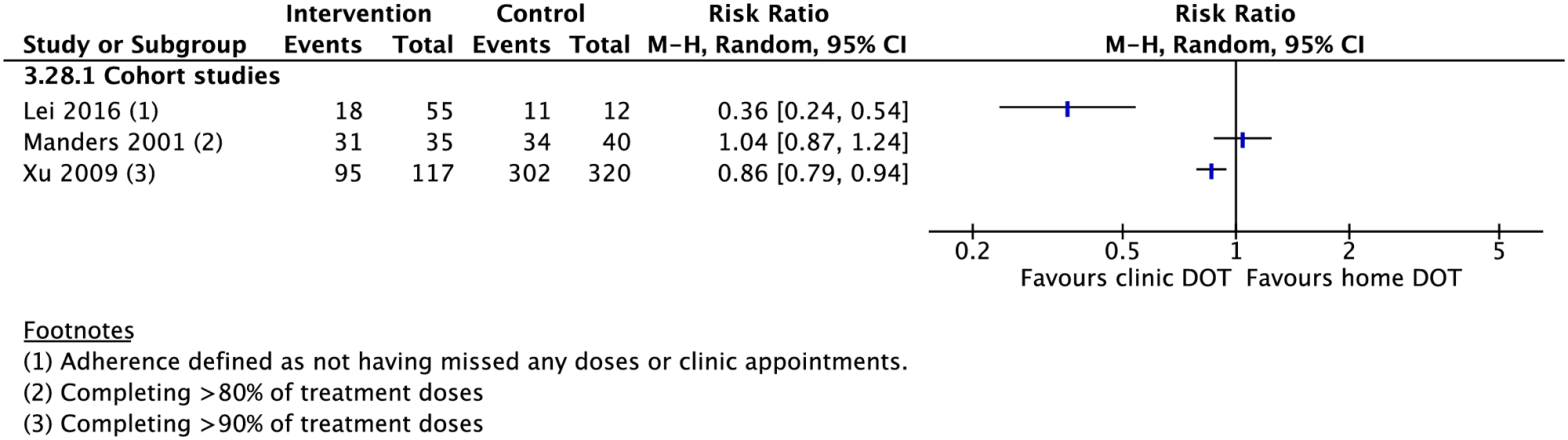
Adherence rates in patients receiving DOT at various locations. DOT, directly observed therapy; M-H, Mantel-Haenszel.

**Fig 13:**
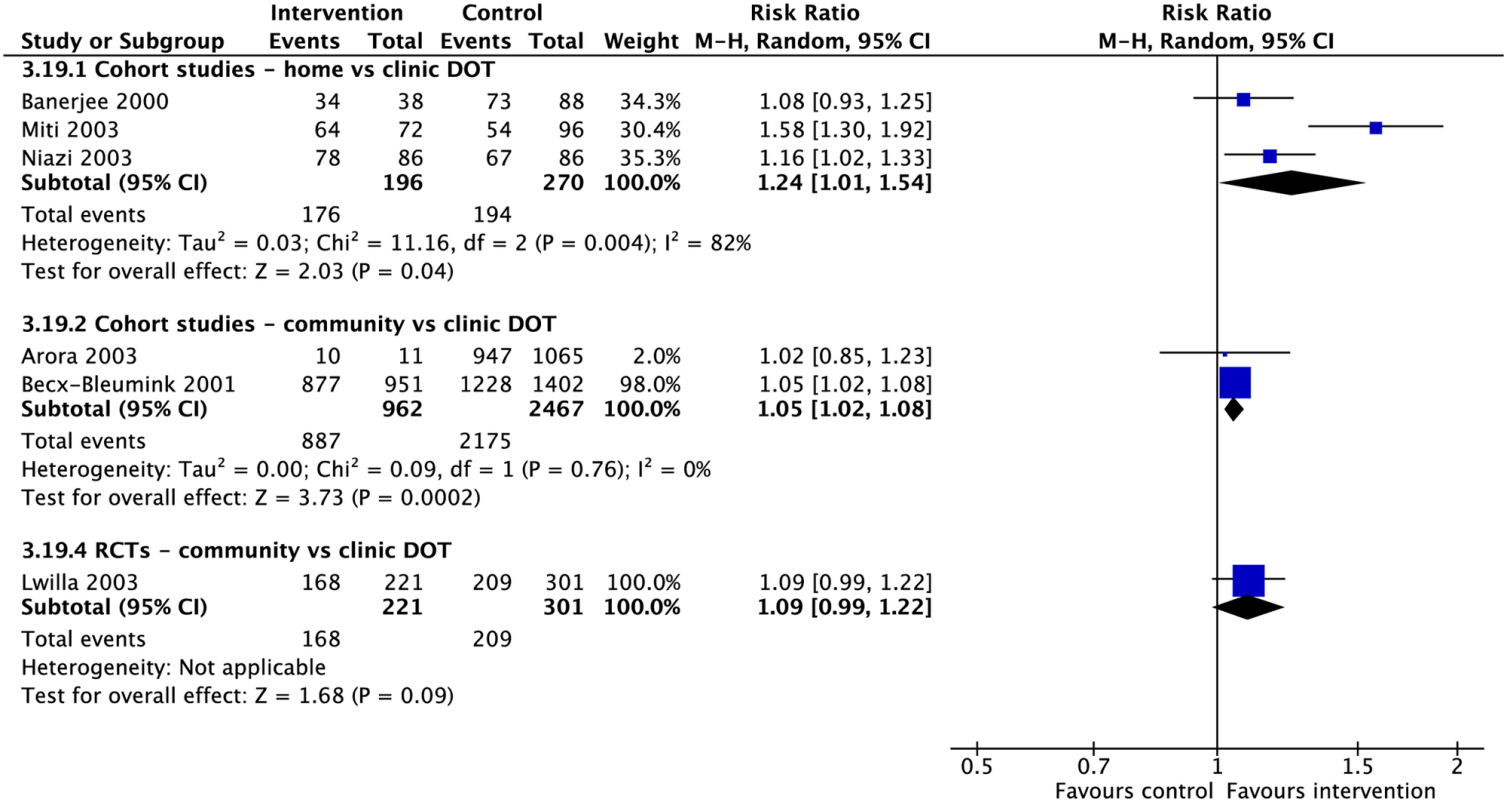
Meta-analysis of sputum conversion rates at two months in patients receiving DOT at various locations. DOT, directly observed therapy; M-H, Mantel-Haenszel.

Four RCTs and 21 CSs compared DOT offered at different locations, including the home, community, and clinic [37,39,42,49,61,73,79–97]. In comparison with clinic-based DOT, treatment in the community was associated with higher rates of treatment completion (1 RCT: RR 2.92, 95% CI 1.15–7.41; 3 CSs: RR 0.87, 95% CI 0.87–1.09) and sputum conversion at the end of two months (2 CSs: RR 1.05, 95% CI 1.02–1.08; 1 RCT: RR 1.09, 95% CI 0.99–1.22) as well as lower rates of loss to follow-up (6 CSs: RR 0.63, 95% CI 0.40–0.98; 2 RCTs: RR 1.04, 95% CI 0.34–3.19), treatment failure (6 CSs: RR 0.56, 95% CI 0.33–0.95; 1 RCT: RR 0.68, 95% CI 0.13–3.69), and unfavorable outcome (1 CS: RR 0.63, 95% CI 0.55–0.73) (Figs 10–13 and S30–S35 Figs). No significant differences were noted for the outcomes of mortality or cure. Home-based DOT was associated with a lower rate of treatment adherence when compared with clinic-based DOT in one CS, in which patients were observed by family members or lay providers (1 CS: RR 0.86, 95% CI 0.79–0.94), while another found no significant difference in adherence rates when comparing home and clinic based DOT. Home-based DOT was associated with a higher mortality rate (8 CSs: RR 1.86, 95% CI 1.34–2.59). This effect is nullified with the removal of the study by Mhimbira and colleagues, who defined home-based DOT as DOT delivered at home by a treatment supporter of patients’ choosing. When compared with a particular type of community-based DOT that included offering a free lunch to patients, home-based DOT had a lower success rate in one CS (1 CS: RR 0.94, 95% CI 0.92–0.98; 2 RCTs: RR 1.02, 95% CI 0.94–1.11) but no significant difference in rates of treatment completion, cure, mortality, failure, and loss to follow-up in other studies. Home-based DOT was associated with marginally higher rates of sputum conversion at two months, when compared with clinic-based DOT (3 CSs: RR 1.24, 95% CI 1.01–1.54).

### Patient education and counseling

**Fig 14:**
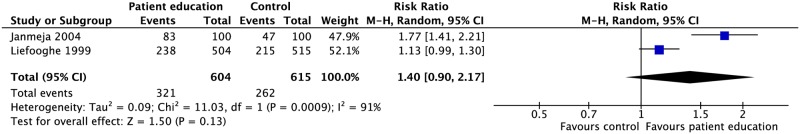
Meta-analysis of treatment success rates in patients receiving patient education and counseling interventions in addition to standard care versus standard care alone. M-H, Mantel-Haenszel.

**Fig 15:**
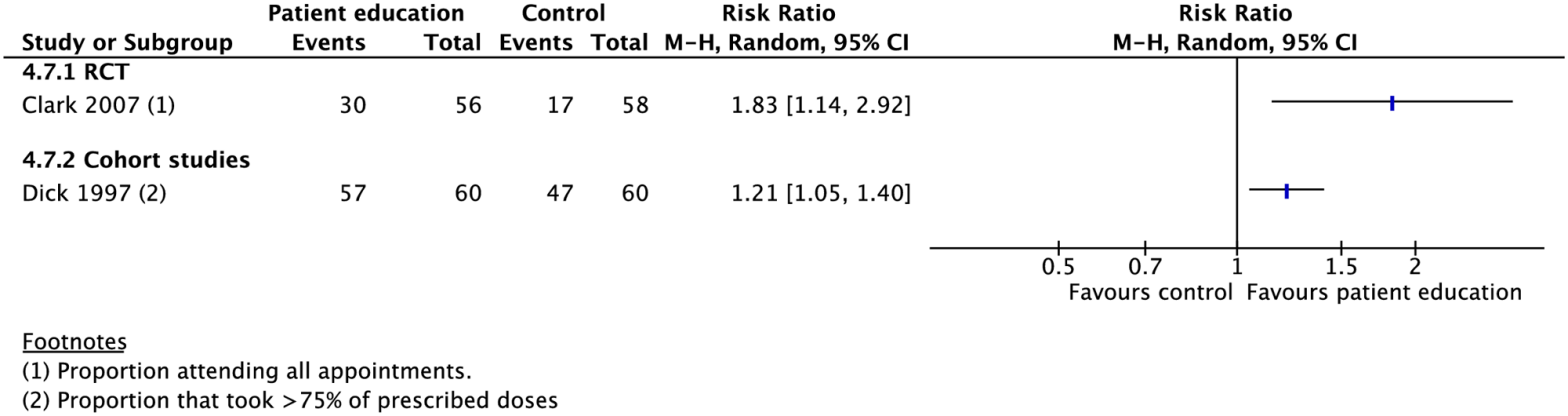
Adherence rates in patients receiving patient education and counseling interventions in addition to standard care versus standard care alone. M-H, Mantel-Haenszel; RCT, randomized controlled trial.

Four RCTs and one CS evaluated the effect of oral and written educational material as well as counseling on TB treatment outcomes [7–11]. Education and counseling was associated with a higher rate of treatment completion (1 RCT: RR 1.71, 95% CI 1.32–2.22), cure (1 RCT: RR 2.15, 95% CI 1.58–2.92), and adherence (1 RCT: RR 1.83, 95% CI 1.14–2.92; 1 CS: RR 1.21, 95% CI 1.05–1.40) (Figs 10, 14, 15 and S36–S40 Figs). These interventions had no meaningful impact on rates of mortality, treatment success, failure, or loss to follow-up.

### Incentives and enablers

**Fig 16:**
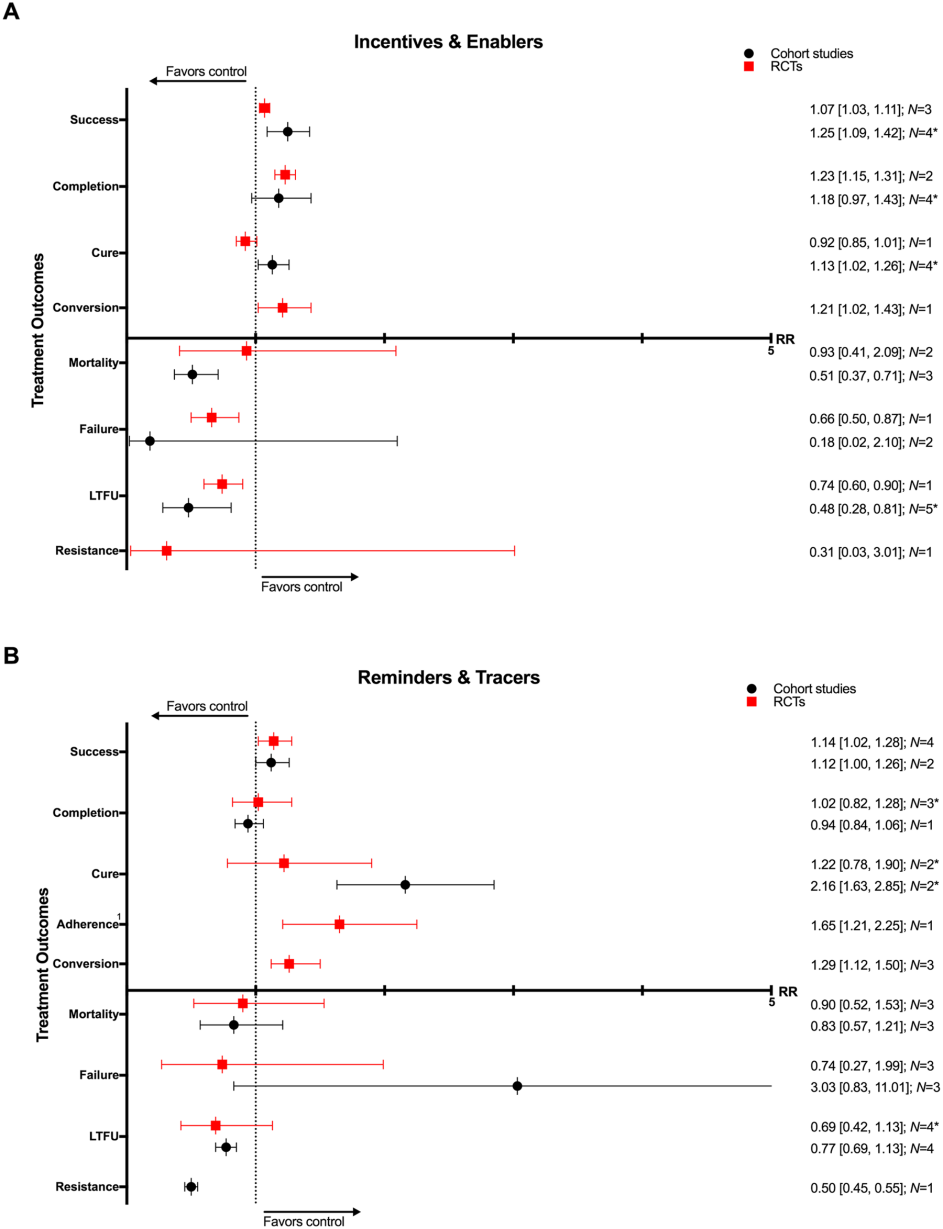
(A) Impact of incentives and enablers on TB treatment outcomes. (B) Impact of reminders and tracers on TB treatment outcomes. * = significant heterogeneity in the meta-analysis as determined by I^2^ statistic. 1 = adherence defined as the proportion of patients who presented for all drug collections in the first six months of treatment. Conversion = sputum conversion at the end of two months. Resistance = development of drug resistance. *N* = number of studies included within the meta-analysis. LTFU, loss to follow-up; RCT, randomized controlled trial; RR, risk ratio; TB, tuberculosis.

**Fig 17:**
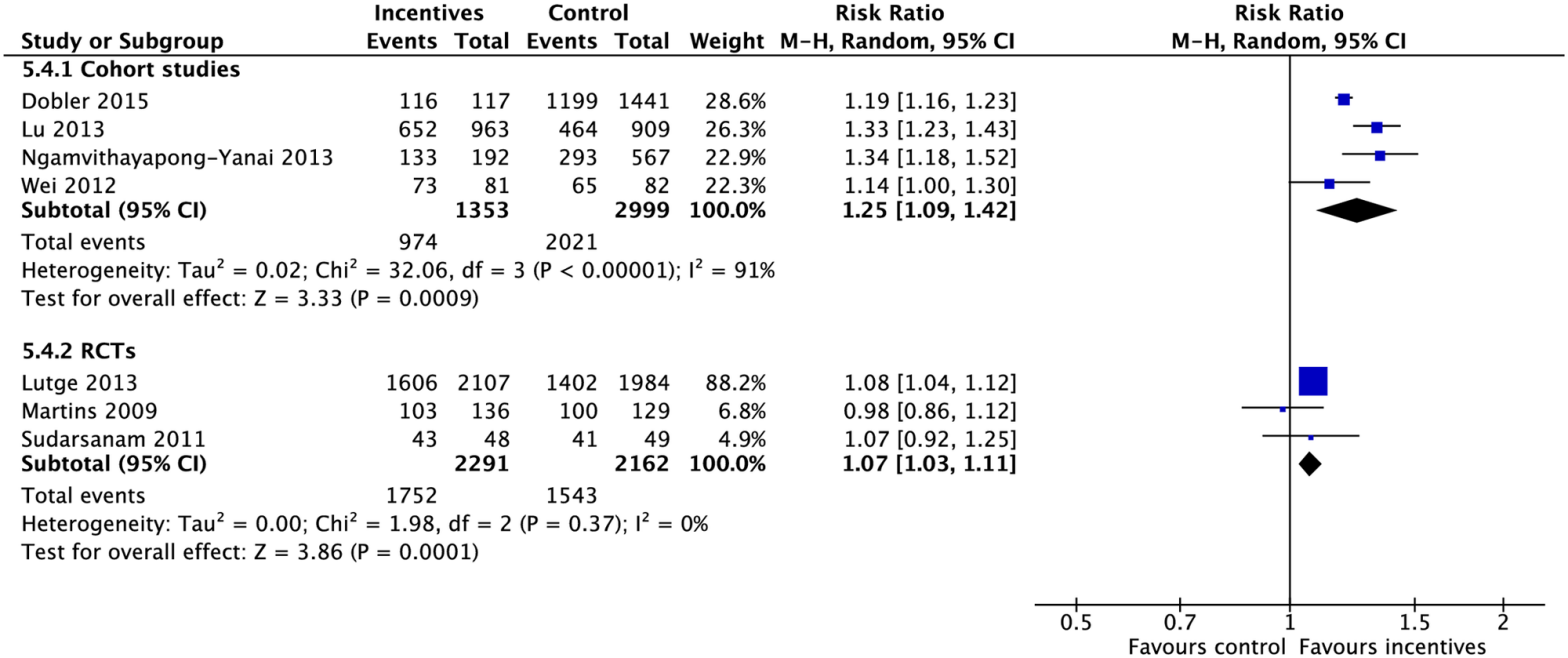
Meta-analysis of treatment success rates in patients receiving incentives and enablers in addition to standard care versus standard care alone. M-H, Mantel-Haenszel; RCT, randomized controlled trial.

Incentives and enablers in four RCTs and eleven CSs were associated with lower rates of mortality (3 CSs: RR 0.51, 95% CI 0.37–0.71; 2 RCTs: RR 0.93, 95% CI 0.41–2.09), treatment failure (1 RCT: RR 0.66, 95% CI 0.50–0.87; 2 CSs: RR 0.18, 95% CI 0.02–2.10), and loss to follow-up (1 RCT: RR 0.74, 95% CI 0.60–0.90; 5 CSs: RR 0.48, 95% CI 0.28–0.81) as well as higher rates of treatment success (3 RCTs: RR 1.07, 95% CI 1.03–1.11; 4 CSs: RR 1.25, 95% CI 1.09–1.42), completion (2 RCTs: RR 1.23, 95% CI 1.15–1.31; 4 CSs: RR 1.18, 95% CI 0.97–1.43), cure (4 CSs: 1.13, 95% CI 1.02–1.26; 1 RCT: RR 0.92, 95% CI 0.85–1.01), and sputum conversion at two months (1 RCT: RR 1.21, 95% CI 1.02–1.43). (Figs 16, 17 and S41–S47 Figs) [83,98–111].

### Reminders and tracers

**Fig 18:**
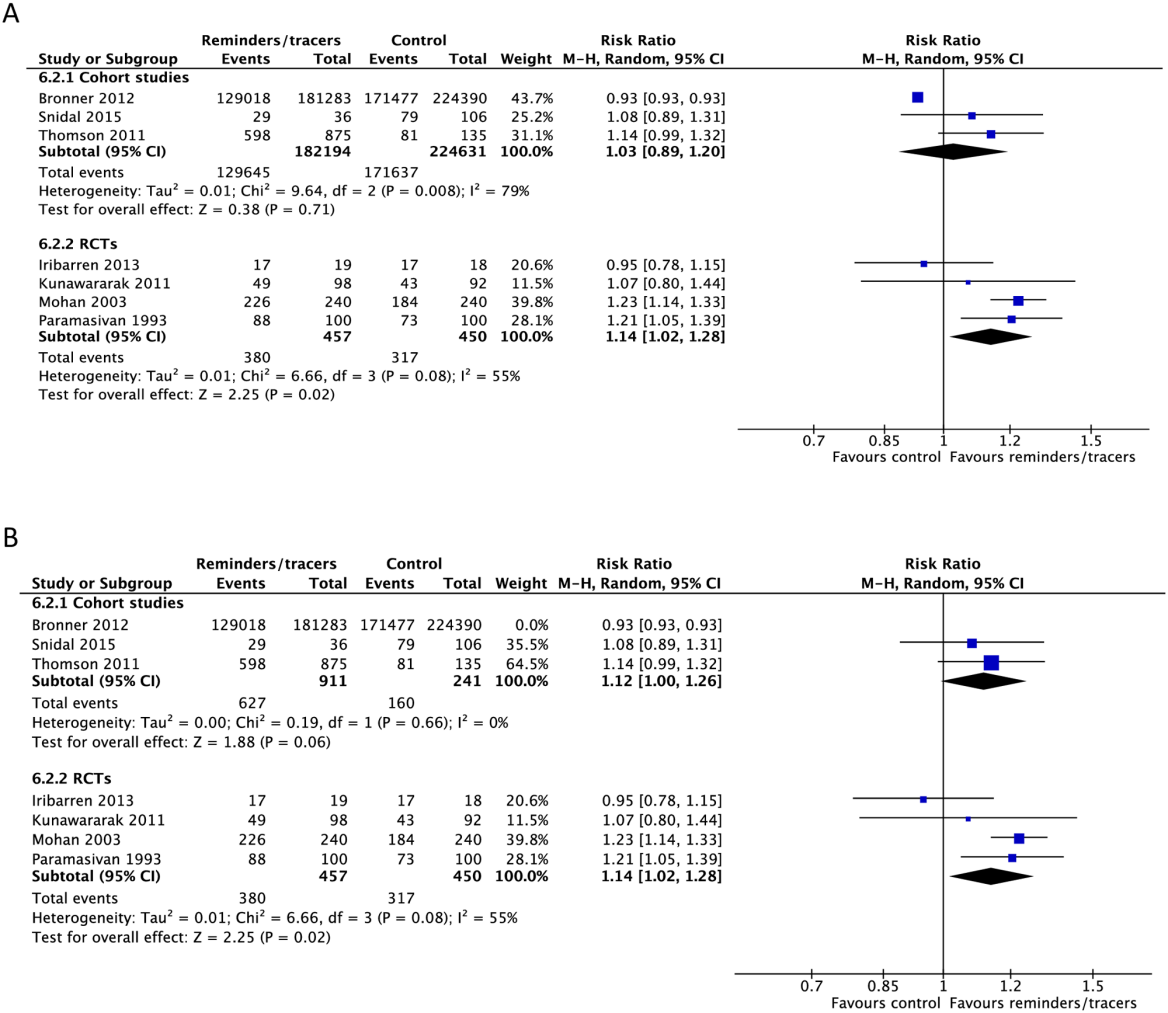
(A) Meta-analysis of treatment success rates in patients receiving reminders/tracers in addition to standard care versus standard care alone. (B) Sensitivity analysis: removing the heaviest weighted study (Bronner 2012) in which control and intervention cohorts had different pre-intervention success rates. M-H, Mantel-Haenszel; RCT, randomized controlled trial.

**Fig 19:**
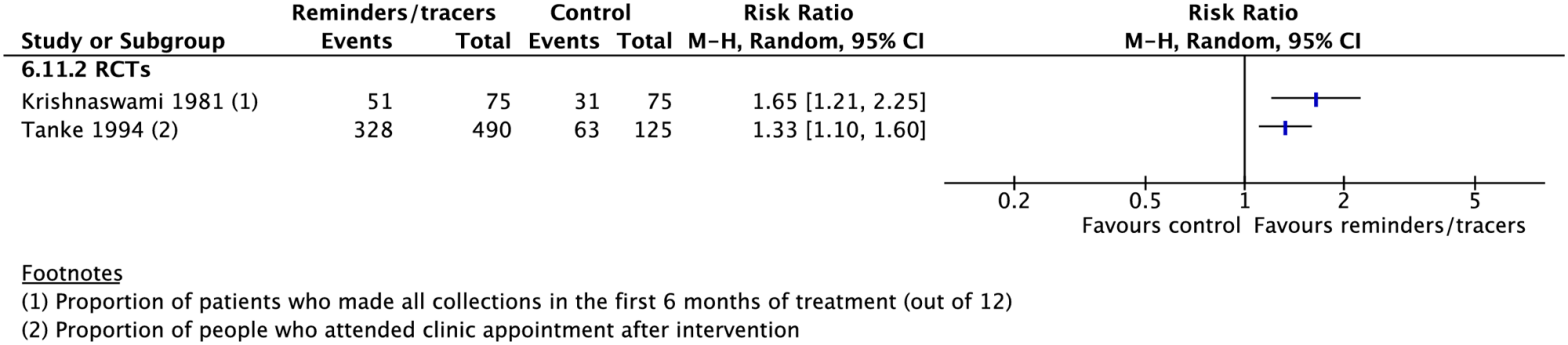
Adherence rates in patients receiving reminders/tracers in addition to standard care versus standard care alone. M-H, Mantel-Haenszel; RCT, randomized controlled trial.

**Fig 20:**
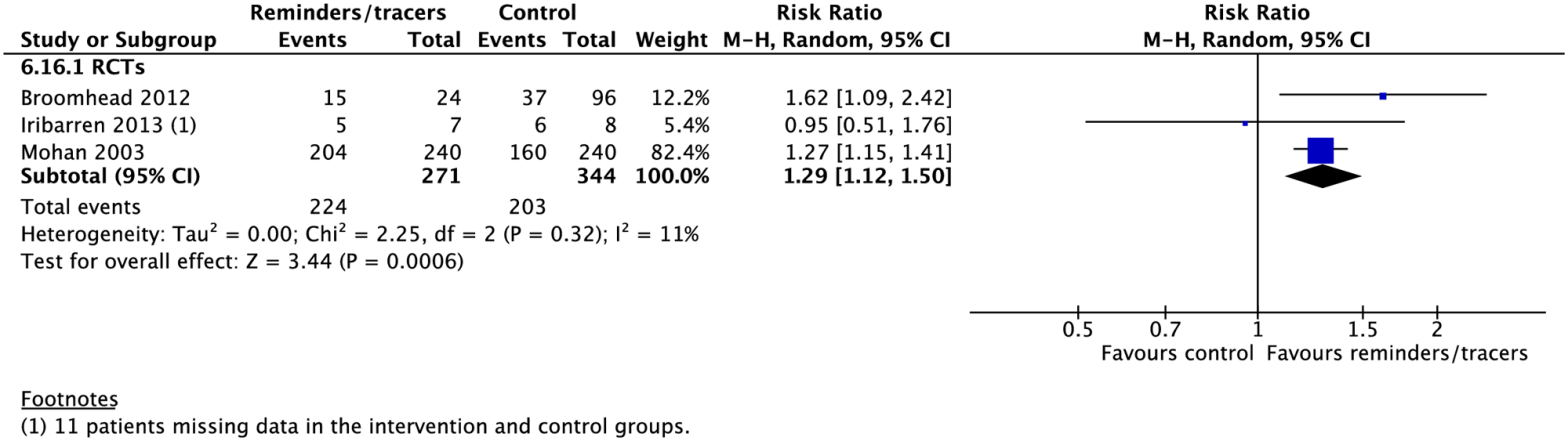
Meta-analysis of rates of sputum conversion at two months in patients receiving reminders/tracers in addition to standard care versus standard care alone. M-H, Mantel-Haenszel; RCT, randomized controlled trial.

Nine RCTs and six CSs found higher rates of treatment success (4 RCTs: RR 1.14, 95% CI 1.02–1.28; 2 CSs: RR 1.12, 95% CI 1.00–1.26), cure (2 CSs: RR 2.16, 95% CI 1.63–2.85; 2 RCTs: RR 1.22, 95% CI 0.78–1.90), adherence (1 RCT: RR 1.65, 95% CI 1.21–2.25), and sputum conversion at two months (3 RCTs: RR 1.29, 95% CI 1.12–1.50) and lower rates of development of drug resistance (1 CS: RR 0.50, 95% CI 0.45–0.55) and loss to follow-up (4 CSs: RR 0.77, 95% CI 0.69–1.13; 4 RCTs: RR 0.69, 95% CI 0.42–1.13) with the use of reminders and tracers (Figs 16, 18–20 and S48–S55 Figs) [112–126]. One study by Bronner and colleagues was removed in sensitivity analyses due to lack of controlling for baseline differences in treatment outcomes prior to intervention in the intervention and control districts (Fig 18).

### Staff education

**Fig 21:**
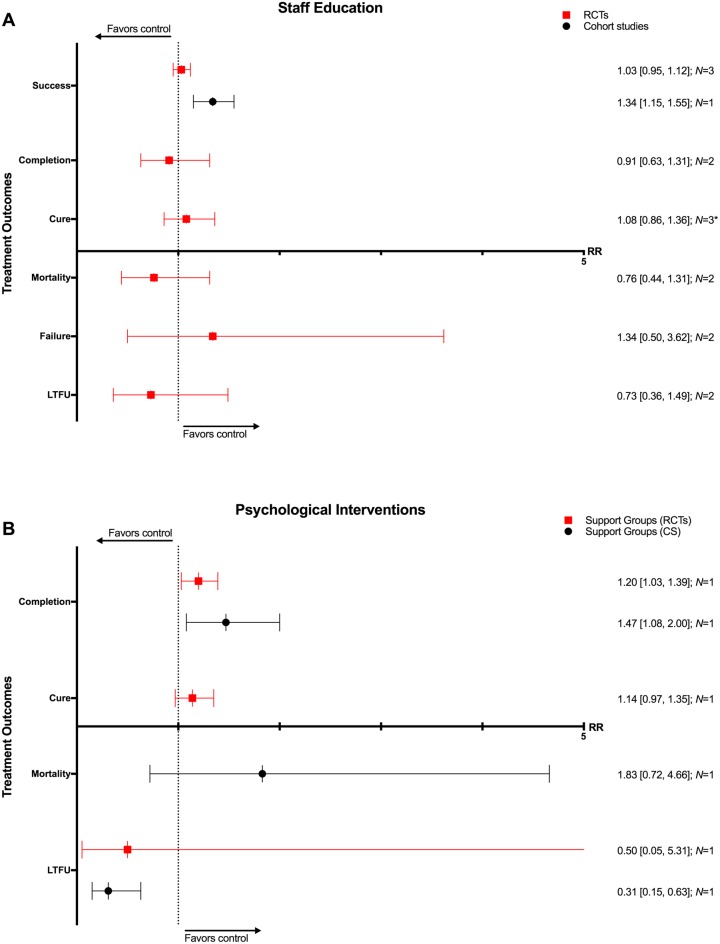
(A) Impact of staff education on TB treatment outcomes. (B) Impact of using psychological interventions, such as mental health counseling and support groups, on TB treatment outcomes. * = significant heterogeneity in the meta-analysis, as determined by I^2^ statistic. *N* = number of studies included within the meta-analysis. CS, cohort study; LTFU, loss to follow-up; RCT, randomized controlled trial; RR, risk ratio; TB, tuberculosis.

**Fig 22:**
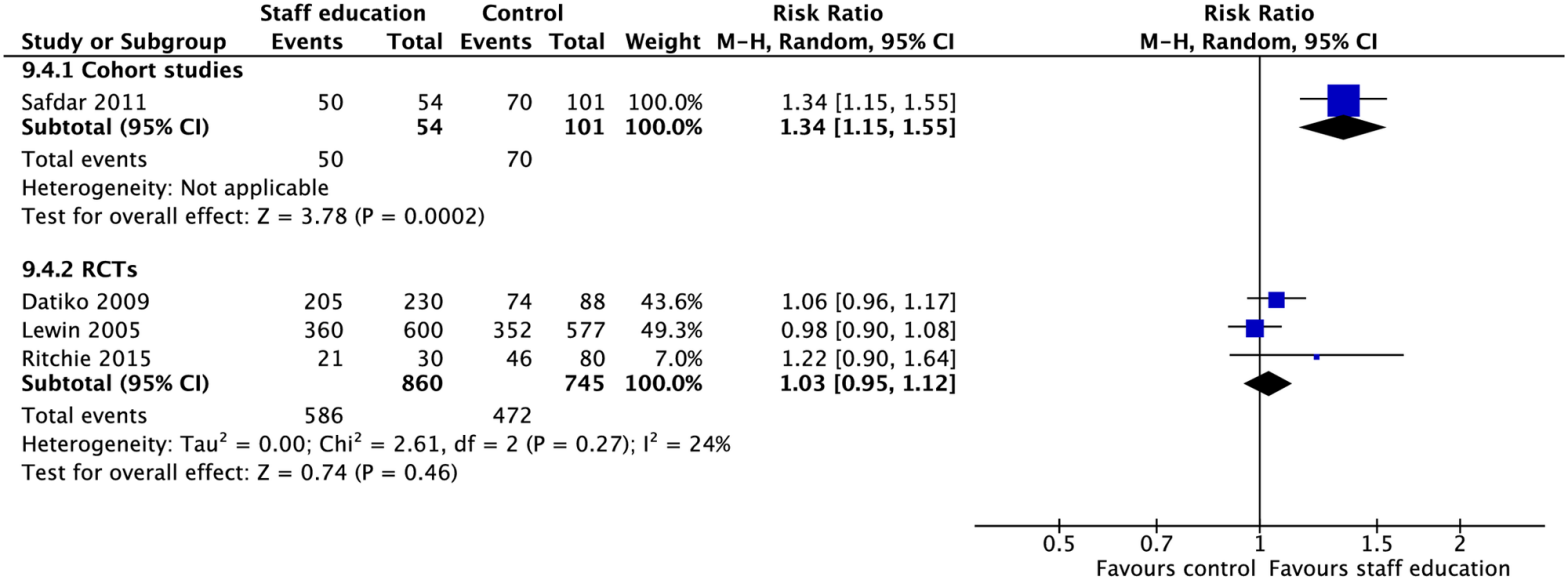
Meta-analysis of the impact of staff education on treatment success rates. M-H, Mantel-Haenszel; RCT, randomized controlled trial.

Three RCTs and one CS involved interventions such as adherence education for staff, peer training for lay health workers, reminders to initiate adherence discussions, and educational tools and aids for decision-making [127–130]. These interventions were associated with higher rates of treatment success (1 CS: RR 1.34, 95% CI 1.15–1.55) and lower rates of loss to follow-up (1 CS: RD −0.18, 95% CI −0.26–−0.10) in the one CS. The three RCTs found no significant difference in rates of TB treatment outcomes with the use of such interventions (Figs 21, 22 and S56–S61 Figs).

### Psychological interventions

One RCT focused on brief counseling interventions for alcohol cessation and another offered self-help groups [131,132]. One prospective CS evaluated the impact of TB clubs as a support network [133]. Support groups were associated with higher rates of treatment completion (1 CS: RR 1.47, 95% CI 1.08–2.00; 1 RCT: RR 1.20, 95% CI 1.03–1.39) and lower rates of treatment failure (1 RCT: RD −0.12, 95%CI −0.22–−0.01; 1 CS: RD −0.02, 95% CI −0.06–0.03) and loss to follow-up (1 CS: RR 0.31, 95% CI 0.15–0.63; 1 RCT: RR 0.50, 95% CI 0.05–5.31) (Fig 21 and S62–S67 Figs).

### Digital health

**Fig 23:**
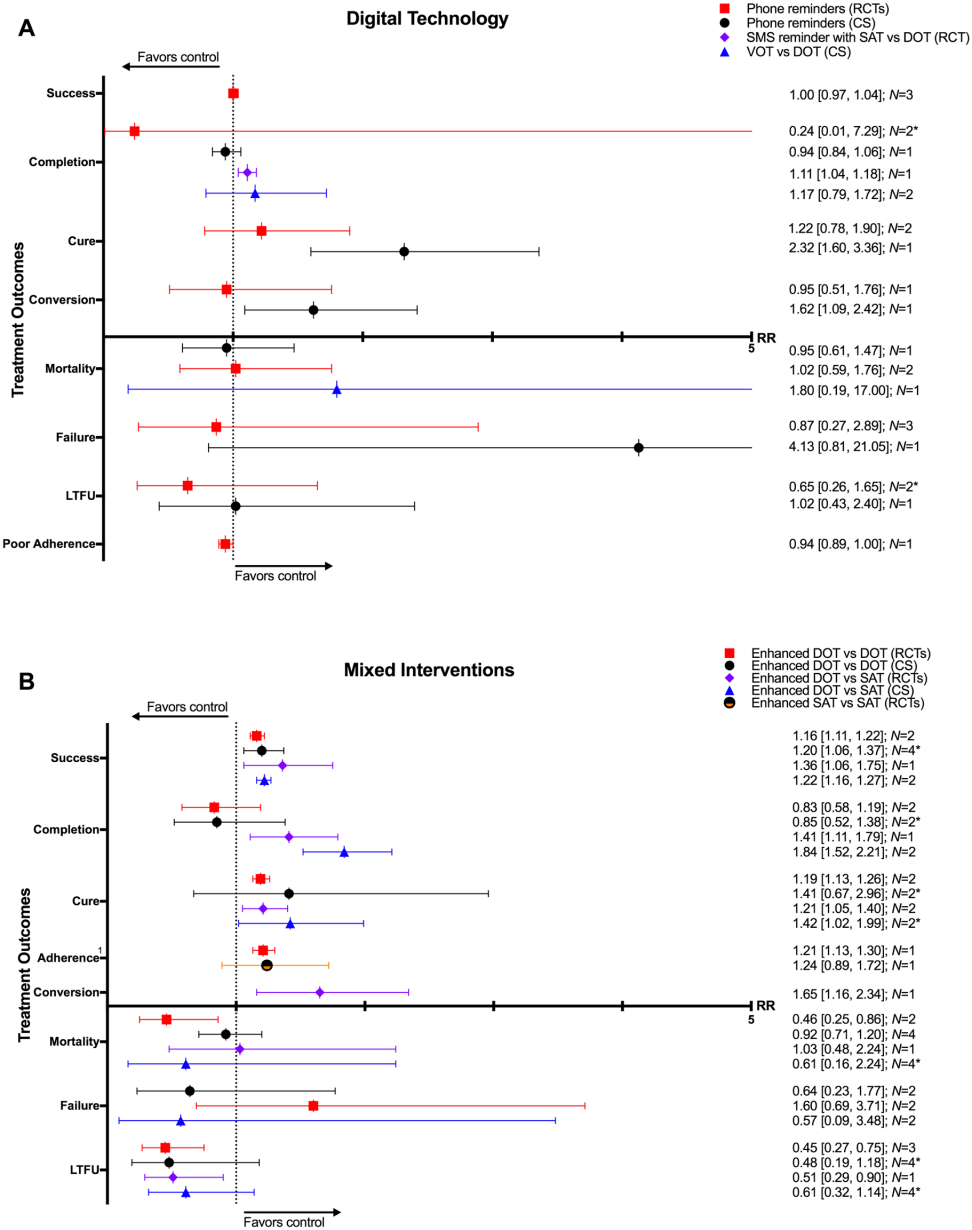
(A) Impact of digital technologies on TB treatment outcomes. (B) Impact of combining different types of adherence interventions on TB treatment outcomes. * = significant heterogeneity in the meta-analysis, as determined by I^2^ statistic. 1 = defined as proportion of patients taking >90% of pills. Conversion = sputum conversion at the end of two months. *N* = number of studies included within the meta-analysis. Poor adherence = percentage of patient-months in which >20% of doses were missed. CS, cohort study; DOT, directly observed therapy; LTFU, loss to follow-up; RCT, randomized controlled trial; RR, risk ratio; TB, tuberculosis; VOT, video-observed therapy.

**Fig 24:**
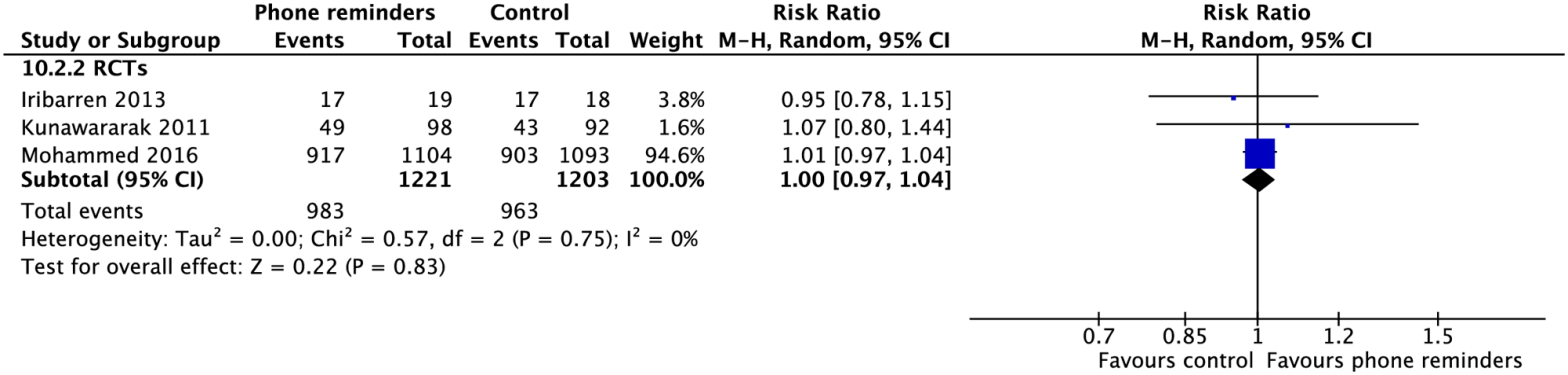
Meta-analysis of treatment success rates in patients receiving phone reminders in addition to standard care versus standard care alone. M-H, Mantel-Haenszel; RCT, randomized controlled trial.

**Fig 25:**
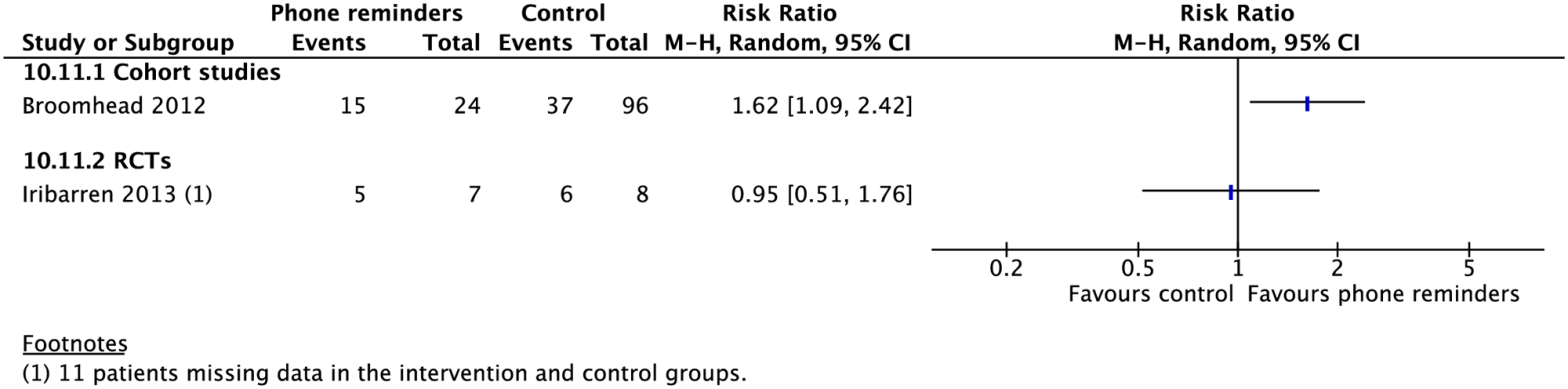
Rates of sputum conversion at two months in patients receiving phone reminders in addition to standard care versus standard care alone. M-H, Mantel-Haenszel; RCT, randomized controlled trial.

Five RCTs and two CSs looked at daily reminder texts or phone call reminders to take medications for patients undergoing SAT or family DOT [114,117,119,120,125,126,134]. Two CSs evaluated the impact of VOT [135,136]. In one RCT comparing SMS reminders in patients undergoing SAT versus those undergoing DOT, SAT patients had a higher treatment completion rate (1 RCT: RR 1.11, 95% CI 1.04–1.18). One CS on patients using a combination wireless pill box/SMS reminder system found higher rates of cure (RR 2.32, 95% CI 1.60–3.36) and sputum conversion at two months (RR 1.62, 95% CI 1.09–2.42) compared to those without this system. Other studies found no difference in rates of mortality, treatment success, failure, or loss to follow-up (Figs 23–25 and S68–S74 Figs).

Electronic medication monitor boxes were associated with lower rates of loss to follow-up (1 CS: RR 0.59, 95% CI 0.43–0.80), poor outcome (1 CS: RR 0.63, 95% CI 0.47–0.83), and poor adherence (1 CS: RR 0.57, 95% CI 0.53–0.61). Phone reminders and medication monitor boxes combined were associated with lower rates of poor adherence (1 CS: RR 0.56, 95% CI 0.52–0.60). Compared with DOT, VOT rates of treatment completion (2 CSs: RR 1.17, 95% CI 0.79–1.72) and mortality (1 CS: RR 1.80, 95% CI 0.19–17) were not significantly different [135,136].

### Mixed interventions

**Fig 26:**
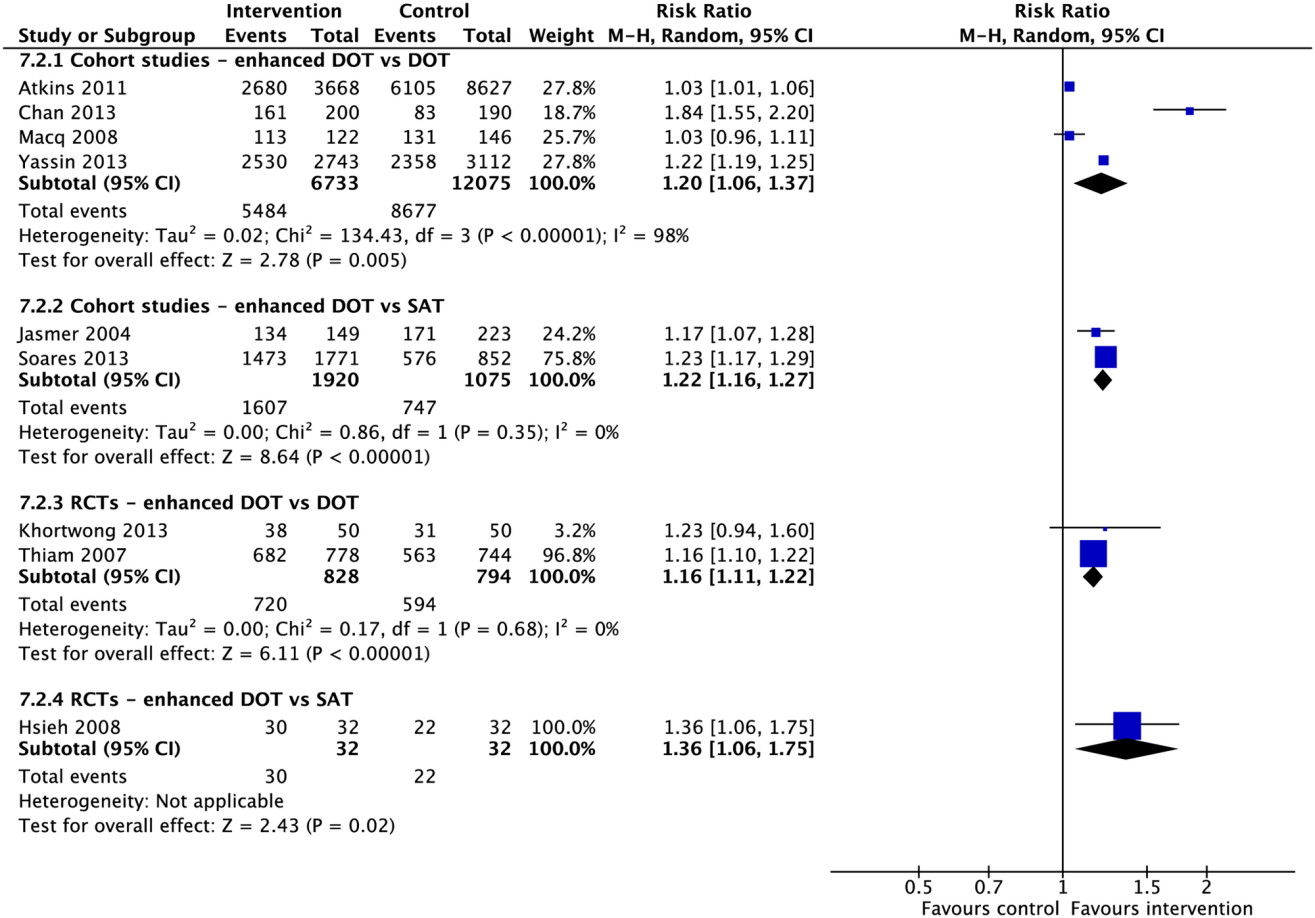
Meta-analysis of treatment success rates in patients receiving combination adherence interventions (enhanced DOT) in addition to standard care versus standard care alone. DOT, directly observed therapy; M-H, Mantel-Haenszel; RCT, randomized controlled trial; SAT, self-administered therapy.

**Fig 27:**
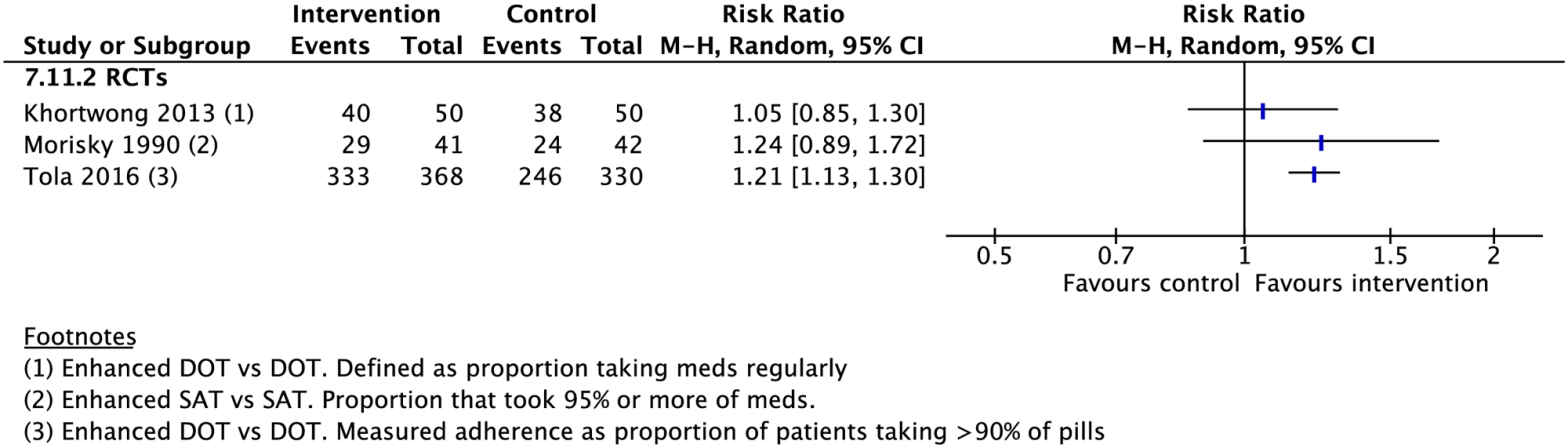
Adherence rates in patients receiving combination adherence interventions (enhanced DOT or enhanced SAT) in addition to standard care versus standard care alone. DOT, directly observed therapy; M-H, Mantel-Haenszel; RCT, randomized controlled trial; SAT, self-administered therapy.

Seven RCTs and eleven CSs combined multiple adherence interventions from the aforementioned categories to DOT or SAT [10,59,137–152]. Patient-centered DOT (enhanced DOT) was associated with lower rates of loss to follow-up (4 RCTs: RR 0.45, 95% CI 0.32–0.65) and higher rates of treatment success (3 CSs: RR 1.27, 95% CI 1.09–1.49; 2 RCTs: RR 1.16, 95% CI 1.11–1.22) and cure (3 RCTs: RR 1.19, 95% CI 1.13–1.25) when compared with DOT alone (Figs 23, 26, 27 and S75–S82 Figs). When compared with SAT, patient-centered DOT was associated with higher rates of treatment success (2 CSs: RR 1.22, 95% CI 1.16–1.27; 1 RCT: RR 1.36, 95% CI 1.06–1.75), treatment completion (2 CSs: RR 1.84, 95% CI 1.52–2.21; 1 RCT: RR 1.41, 95% CI 1.11–1.79), cure (2 CSs: RR 1.42, 95% CI 1.02–1.99; 1 RCT: RR 1.36, 95% CI 1.06–1.75), and sputum conversion at two months (1 RCT: RR 1.65, 95% CI 1.16–2.34). We did not find any studies that compared patient-centered DOT to patient-centered SAT.

## Discussion

To our knowledge, this is the first comprehensive systematic review of prospective and retrospective CSs as well as RCTs studying any interventions used to increase adherence to treatment of either drug-susceptible or -resistant TB. The studies included represent high- and low-resource settings and therefore are broadly representative across a variety of TB program settings. Our meta-analysis identified several adherence interventions that consistently showed associations with improved adherence and TB treatment outcomes. Of note, DOT was associated with better treatment outcomes compared to SAT, especially for TB patients living with HIV. The addition of other adherence interventions to DOT, such as education (for staff or patients), material or psychological support, or reminder systems (including SMS technology and phone reminders), correlated with reduced rates of mortality and loss to follow-up and higher rates of treatment success and cure. Combining other types of adherence interventions with DOT or SAT was associated with improved outcomes compared to either treatment modality alone. Lastly, VOT had no evidence of a difference in rates of treatment completion and mortality when compared to DOT.

Our conclusions differ from prior reviews. Karumbi and colleagues focused on data from RCTs and concluded that DOT did not improve TB treatment completion and cure when compared to self-administered therapy [153]. When our meta-analysis was limited to datasets from RCTs, we found similar results on these outcomes. Whereas RCTs are a superior study design because of their strong internal validity, they can lack external validity [154–156]. For example, RCTs provide rigorous oversight of participants that does not correspond to routine practice in program settings. Furthermore, without allocation concealment, alterations of behavior because of the awareness of being observed can further hamper the ability of RCTs to assess the value of adherence interventions. Another review by Pasipanodya and colleagues included RCTs as well as prospective CSs and concluded that DOT was not significantly better than SAT in preventing microbiologic failure, relapse, or development of drug resistance [157]. Our review found similar results with respect to these particular outcomes of interest. However, because our inclusion criteria were broader than these three outcomes, we included a larger number of CSs and RCTs in our review, resulting in a larger dataset for analysis.

Important findings in our analysis are that a variety of adherence interventions are effective in improving TB outcomes, certain subpopulations are more likely to benefit from particular types of interventions (i.e., DOT for TB patients with HIV), and certain modalities of DOT are more effective than others. Furthermore, treatment supervision alone in the form of DOT is not likely to guarantee improved TB treatment outcomes in all TB patients across all settings. As such, a patient-centered care approach to TB adherence using a package of interventions tailored to a patient’s needs and values is more likely to improve TB outcomes. It must be noted that most of the literature on adherence interventions included in our meta-analysis did not evaluate the impact of adherence interventions on relapse and development of drug resistance, which are critical outcomes of interest for population-level TB management [2]. Only three CSs compared the impact of DOT on the development of drug resistance compared with SAT, one of which found no significant difference [4,59,70]. One CS found reminders correlated with reduced rates of drug resistance [113], and another CS on incentives found no benefit [109]. With regard to relapse rates, one RCT [158] and four CSs [40,54,59,67] found no significant differences between DOT and SAT, while two CSs saw a decline in relapse rates with DOT [4,38]. Lastly, relapse rates were similar between patient-centered DOT and SAT in one CS [59]. Given the paucity of data on such critical outcomes, the effectiveness of adherence interventions based on our analyses should be assessed cautiously.

DOT has several limitations, including the cost imposed on patients and the health system [33–35,49,159,160]. Furthermore, WHO’s guidance on TB ethics argues that DOT is only ethically justifiable in the context of a patient-centered approach and provides an ethical framework for its implementation [161,162]. It must be noted that many of the adherence interventions in these studies involve significant resource utilization, which makes it necessary to mobilize the resources needed to facilitate their implementation. Comparing the cost-effectiveness of different adherence interventions would be crucial to strengthen and expand patient-centered approaches for adherence to TB treatment. Data from studies on digital technology and VOT are promising and have the potential to reduce costs to the patient and the health system [163]. The key challenges to address in implementing SMS and VOT adherence interventions are ensuring access to smartphones, coverage of data transmission costs, data encryption, and patient privacy.

Our meta-analysis has limitations. First, most of the studies on DOT in TB patients living with HIV are from the pre-ART era or were conducted in patients at highest risk of loss to follow-up. Contemporary, more integrated approaches to HIV/TB care were not assessed in this review. Second, the studies in our review were heterogeneous in their methodology. It is difficult to standardize methodology in these types of studies, given the numerous disparate interventions possible, evolving technologies, and the complexities of behavior change. We grouped similar interventions within categories of adherence interventions to draw on their similarities for practice recommendations. As the way in which such interventions are implemented largely determines their success, inter-study variability impacts our meta-analytic findings. Third, our literature review was restricted to English language articles in Medline. The absence of potentially relevant studies published in non-English language journals or via other electronic databases is a limitation. However, we reviewed the references of 32 systematic reviews on this topic with search strategies that spanned European (Embase), Latin American (LILACS), Cochrane Central Register of Controlled Trials (CENTRAL), and metaRegister of Controlled Trials (mRCT) databases. Additionally, we consulted experts in studies of adherence interventions to ascertain that all relevant studies were included. Lastly, the results from meta-analyses of CSs should be interpreted with caution given uncontrolled confounding inherent to such studies, as noted by our quality assessment.

In this review, we sought to evaluate the impact of adherence interventions in the treatment of drug-resistant TB, and whereas some of the studies included in our review had cohorts of patients with drug resistance, no studies focused only on MDR-TB met our inclusion criteria. A systematic review in 2009 that was limited to case series without an internal or historic control group found that rates of treatment success were higher in patients with MDR-TB who received DOT throughout treatment than those who did not [164]. Another meta-analysis also limited to case series found lower rates of loss to follow-up with DOT delivery at home, use of community health workers for DOT (as opposed to nurses or healthcare providers), a standardized treatment regimen, provision of DOT throughout treatment, and patient education [165]. Given the increased length and duration of treatment necessary for patients with MDR-TB, more studies on adherence interventions tailored to this population are needed.

### Conclusion

We have found that TB treatment outcomes improve with the use of adherence interventions such as patient education and counseling, material support, psychological support interventions, reminders and tracers, and digital health technologies. DOT provided by trained health workers in the community is associated with better treatment outcomes than DOT provided by family members or untrained lay workers. DOT provided in the community is associated with better treatment outcomes than clinic-based DOT. TB patients living with HIV have significantly better outcomes when treated with DOT as opposed to SAT. VOT may be an appropriate alternative to in-person DOT if the resources for its use are available. More importantly, a patient-centered approach to TB treatment using a package of adherence interventions tailored to patients’ needs and values leads to improved TB treatment outcomes. The optimal package of adherence interventions to implement may vary by setting, resources, and the local epidemiology of TB (e.g., prevalence of comorbidities, including HIV coinfection), among other factors. The WHO TB treatment guidelines update, for which this review was conducted, provides additional information on selecting patient-centered approaches for enhancing adherence [166]. Based on our review, studies on adherence interventions in patients with MDR-TB as well as cost-effectiveness analyses will be helpful in identifying the optimal interventions to implement in various settings.

## Supporting information

S1 Text PRISMA checklist. (DOC)

S2 Text Systematic review protocol. (DOC)

S1 Table Search protocol for Pubmed/Medline for adherence interventions in TB treatment. TB, tuberculosis.
(DOCX)

S2 Table Search protocol for SMS/video interventions in TB treatment. SMS, short message service; TB, tuberculosis.
(DOCX)

S3 Table Characteristics of included studies. 1 = Newcastle-Ottawa Score provided for cohort studies. Quality of RCTs are presented separately. 2 = Study includes >50% HIV/TB patients. 3 = Study includes >50% MDR-TB patients. RCT, randomized controlled trial; TB, tuberculosis.
(DOCX)

S1 Fig Risk of bias graph: Review authors' judgements about each risk of bias item presented as percentages across all included studies. (TIF)

S2 Fig Risk of bias summary: Review authors' judgements about each risk of bias item for each included study. (TIF)

S3 Fig Funnel plot of studies comparing mortality rates in patients undergoing SAT versus DOT. DOT, directly observed therapy; SAT, self-administered therapy.
(TIF)

S4 Fig Meta-analysis of mortality rates in patients undergoing SAT versus DOT. “Not estimable” denotes a subgroup within a study not included in the meta-analysis. DOT, directly observed therapy; SAT, self-administered therapy.
(TIF)

S5 Fig Funnel plot of studies on treatment completion rates in patients undergoing SAT versus DOT. DOT, directly observed therapy; SAT, self-administered therapy.
(TIF)

S6 Fig Meta-analysis of treatment completion rates in patients undergoing SAT versus DOT. “Not estimable” denotes a subgroup within a study not included in the meta-analysis. DOT, directly observed therapy; SAT, self-administered therapy.
(TIF)

S7 Fig Funnel plot of studies on cure rates in patients undergoing SAT versus DOT. DOT, directly observed therapy; SAT, self-administered therapy.
(TIF)

S8 Fig Meta-analysis of cure rates in patients undergoing SAT versus DOT. “Not estimable” denotes a subgroup within a study not included in the meta-analysis. DOT, directly observed therapy; SAT, self-administered therapy.
(TIF)

S9 Fig Funnel plot of studies on treatment failure rates in patients undergoing SAT versus DOT. DOT, directly observed therapy; SAT, self-administered therapy.
(TIF)

S10 Fig Meta-analysis of treatment failure rates in patients undergoing SAT versus DOT. “Not estimable” denotes a subgroup within a study not included in the meta-analysis. DOT, directly observed therapy; SAT, self-administered therapy.
(TIF)

S11 Fig Funnel plot of studies on rates of loss to follow-up in patients undergoing SAT versus DOT. DOT, directly observed therapy; SAT, self-administered therapy.
(TIF)

S12 Fig Meta-analysis of rates of loss to follow-up in patients undergoing SAT versus DOT. “Not estimable” denotes a subgroup within a study not included in the meta-analysis. DOT, directly observed therapy; SAT, self-administered therapy.
(TIF)

S13 Fig Meta-analysis of relapse rates in patients undergoing SAT versus DOT. DOT, directly observed therapy; SAT, self-administered therapy.
(TIF)

S14 Fig Meta-analysis of rates of development of drug resistance in patients undergoing SAT versus DOT. DOT, directly observed therapy; SAT, self-administered therapy.
(TIF)

S15 Fig Meta-analysis of mortality rates in HIV/TB patients undergoing SAT versus DOT. DOT, directly observed therapy; SAT, self-administered therapy; TB, tuberculosis.
(TIF)

S16 Fig Treatment completion rates in HIV/TB patients undergoing SAT versus DOT. DOT, directly observed therapy; SAT, self-administered therapy; TB, tuberculosis.
(TIF)

S17 Fig Meta-analysis of cure rates in HIV/TB patients undergoing SAT versus DOT. DOT, directly observed therapy; SAT, self-administered therapy; TB, tuberculosis.
(TIF)

S18 Fig Meta-analysis of treatment failure rates in HIV/TB patients undergoing SAT versus DOT. DOT, directly observed therapy; SAT, self-administered therapy; TB, tuberculosis.
(TIF)

S19 Fig Meta-analysis of rates of loss to follow-up in HIV/TB patients undergoing SAT versus DOT. DOT, directly observed therapy; SAT, self-administered therapy; TB, tuberculosis.
(TIF)

S20 Fig Relapse rates in HIV/TB patients undergoing SAT versus DOT. DOT, directly observed therapy; SAT, self-administered therapy; TB, tuberculosis.
(TIF)

S21 Fig Mortality rates in MDR-TB patients undergoing SAT versus DOT. DOT, directly observed therapy; MDR-TB, multidrug-resistant tuberculosis; SAT, self-administered therapy.
(TIF)

S22 Fig Treatment completion rate in MDR-TB patients undergoing SAT versus DOT. DOT, directly observed therapy; MDR-TB, multidrug-resistant tuberculosis; SAT, self-administered therapy.
(TIF)

S23 Fig Treatment failure rate in MDR-TB patients undergoing SAT versus DOT. DOT, directly observed therapy; MDR-TB, multidrug-resistant tuberculosis; SAT, self-administered therapy.
(TIF)

S24 Fig Rate of loss to follow-up in MDR-TB patients undergoing SAT versus DOT. DOT, directly observed therapy; MDR-TB, multidrug-resistant tuberculosis; SAT, self-administered therapy.
(TIF)

S25 Fig Meta-analysis of mortality rates in patients receiving DOT by different types of providers. DOT, directly observed therapy.
(TIF)

S26 Fig Meta-analysis of treatment completion rates in patients receiving DOT by different types of providers. DOT, directly observed therapy.
(TIF)

S27 Fig Meta-analysis of cure rates in patients receiving DOT by different types of providers. DOT, directly observed therapy.
(TIF)

S28 Fig Meta-analysis of treatment failure rates in patients receiving DOT by different types of providers. DOT, directly observed therapy.
(TIF)

S29 Fig Meta-analysis of rates of loss to follow-up in patients receiving DOT by different types of providers. DOT, directly observed therapy.
(TIF)

S30 Fig Meta-analysis of mortality rates in patients receiving DOT in different locations. DOT, directly observed therapy.
(TIF)

S31 Fig Meta-analysis of treatment completion rates in patients receiving DOT in different locations. DOT, directly observed therapy.
(TIF)

S32 Fig Meta-analysis of cure rates in patients receiving DOT in different locations. DOT, directly observed therapy.
(TIF)

S33 Fig Meta-analysis of treatment failure rates in patients receiving DOT in different locations. DOT, directly observed therapy.
(TIF)

S34 Fig Meta-analysis of rates of loss to follow-up in patients receiving DOT in different locations. DOT, directly observed therapy.
(TIF)

S35 Fig Rates of unfavorable outcome in patients receiving DOT in different locations. DOT, directly observed therapy.
(TIF)

S36 Fig Meta-analysis of mortality rates in patients receiving educational interventions in addition to standard care versus standard care alone. (TIF)

S37 Fig Treatment completion rates in patients receiving educational interventions in addition to standard care versus standard care alone. (TIF)

S38 Fig Cure rates in patients receiving educational interventions in addition to standard care versus standard care alone. (TIF)

S39 Fig Treatment failure rates in patients receiving educational interventions in addition to standard care versus standard care alone. (TIF)

S40 Fig Meta-analysis of rates of loss to follow-up in patients receiving educational interventions in addition to standard care versus standard care alone. (TIF)

S41 Fig Meta-analysis of mortality rates in patients receiving incentives/enablers in addition to standard care versus standard care alone. (TIF)

S42 Fig Meta-analysis of treatment completion rates in patients receiving incentives/enablers in addition to standard care versus standard care alone. (TIF)

S43 Fig Meta-analysis of cure rates in patients receiving incentives/enablers in addition to standard care versus standard care alone. (TIF)

S44 Fig Meta-analysis of treatment failure rates in patients receiving incentives/enablers in addition to standard care versus standard care alone. (TIF)

S45 Fig Meta-analysis of rates of loss to follow-up in patients receiving incentives/enablers in addition to standard care versus standard care alone. (TIF)

S46 Fig Rates of development of drug resistance in patients receiving incentives/enablers in addition to standard care versus standard care alone. (TIF)

S47 Fig Rates of sputum conversion at two months in patients receiving incentives/enablers in addition to standard care versus standard care alone. (TIF)

S48 Fig (A) Meta-analysis of mortality rates in patients receiving reminders/tracers in addition to standard care versus standard care alone. (B) Sensitivity analysis: removing the heaviest weighted study (Bronner 2012) in which control and intervention cohorts had significantly different pre-intervention mortality rates. “Not estimable” denotes a subgroup within a study not included in the meta-analysis.
(TIF)

S49 Fig Meta-analysis of completion rates in patients receiving reminders/tracers in addition to standard care versus standard care alone. (TIF)

S50 Fig (A) Meta-analysis of cure rates in patients receiving reminders/tracers in addition to standard care versus standard care alone. (B) Sensitivity analysis: removing the heaviest weighted study (Bronner 2012) in which control and intervention cohorts had significantly different pre-intervention cure rates.
(TIF)

S51 Fig (A) Meta-analysis of treatment failure rates in patients receiving reminders/tracers in addition to standard care versus standard care alone—cohort studies. (B) Sensitivity analysis: removing the heaviest weighted study (Bronner 2012) in which control and intervention cohorts had significantly different pre-intervention treatment failure rates.
(TIF)

S52 Fig Meta-analysis of treatment failure rates in patients receiving reminders/tracers in addition to standard care versus standard care alone—RCTs. RCT, randomized controlled trial.
(TIF)

S53 Fig (A) Meta-analysis of rates of loss to follow-up in patients receiving reminders/tracers in addition to standard care versus standard care alone. (B) Sensitivity analysis: removing the heaviest weighted study (Bronner 2012) in which control and intervention cohorts had significantly different pre-intervention loss to follow-up rates.
(TIF)

S54 Fig Rates of development of drug resistance in patients receiving reminders/tracers in addition to standard care versus standard care alone. (TIF)

S55 Fig Rates of poor adherence in patients receiving reminders/tracers in addition to standard care versus standard care alone. Poor adherence is defined as the percentage of patient-months in which at least 20% of doses were missed.
(TIF)

S56 Fig Meta-analysis of mortality rates after using staff educational interventions in addition to standard care versus standard care alone. (TIF)

S57 Fig Meta-analysis of treatment completion rates after using staff educational interventions in addition to standard care versus standard care alone. (TIF)

S58 Fig Meta-analysis of cure rates after using staff educational interventions in addition to standard care versus standard care alone. (TIF)

S59 Fig Meta-analysis of treatment failure rates after using staff educational interventions in addition to standard care versus standard care alone. (TIF)

S60 Fig Meta-analysis of rates of loss to follow-up after using staff educational interventions in addition to standard care versus standard care alone—RCTs. RCT, randomized controlled trial.
(TIF)

S61 Fig Rates of loss to follow-up after using staff educational interventions in addition to standard care versus standard care alone—Cohort studies. (TIF)

S62 Fig Mortality rates in patients receiving psychological interventions in addition to standard care versus standard care alone. (TIF)

S63 Fig Treatment success rates in patients receiving psychological interventions in addition to standard care versus standard care alone. (TIF)

S64 Fig Treatment completion rates in patients receiving psychological interventions in addition to standard care versus standard care alone. (TIF)

S65 Fig Cure rates in patients receiving psychological interventions in addition to standard care versus standard care alone. (TIF)

S66 Fig Treatment failure rates in patients receiving psychological interventions in addition to standard care versus standard care alone. (TIF)

S67 Fig Rates of loss to follow-up in patients receiving psychological interventions in addition to standard care versus standard care alone. (TIF)

S68 Fig Meta-analysis of mortality rates in patients receiving VOT or SMS reminders in addition to standard care versus standard care alone. SMS, short message service; VOT, video-observed therapy.
(TIF)

S69 Fig Meta-analysis of treatment completion rates in patients receiving VOT or SMS/phone reminders in addition to standard care versus standard care alone—Cohort studies. SMS, short message service; VOT, video-observed therapy.
(TIF)

S70 Fig Meta-analysis of treatment completion rates in patients receiving SMS/phone reminders in addition to standard care versus standard care alone—RCTs. RCT, randomized controlled trial; SMS, short message service.
(TIF)

S71 Fig Meta-analysis of cure rates in patients receiving SMS/phone reminders in addition to standard care versus standard care alone. SMS, short message service.
(TIF)

S72 Fig Meta-analysis of cure rates in patients receiving SMS/phone reminders in addition to standard care versus standard care alone. SMS, short message service.
(TIF)

S73 Fig Rates of loss to follow-up in patients receiving SMS/phone reminders in addition to standard care versus standard care alone. SMS, short message service.
(TIF)

S74 Fig Rates of poor adherence in patients receiving SMS/phone reminders in addition to standard care versus standard care alone. Poor adherence is defined as the percentage of patient-months in which at least 20% of doses were missed. SMS, short message service.
(TIF)

S75 Fig Meta-analysis of mortality rates in patients receiving combinations of adherence interventions in addition to standard care versus standard care alone. (TIF)

S76 Fig Meta-analysis of treatment completion rates in patients receiving combinations of adherence interventions in addition to standard care versus standard care alone. (TIF)

S77 Fig Meta-analysis of cure rates in patients receiving combinations of adherence interventions in addition to standard care versus standard care alone. (TIF)

S78 Fig Meta-analysis of treatment failure rates in patients receiving combinations of adherence interventions in addition to standard care versus standard care alone. (TIF)

S79 Fig Meta-analysis of rates of loss to follow-up in patients receiving combinations of adherence interventions in addition to standard care versus standard care alone. (TIF)

S80 Fig Relapse rates in patients receiving combinations of adherence interventions in addition to standard care versus standard care alone. (TIF)

S81 Fig Rates of sputum conversion at two months in patients receiving combinations of adherence interventions in addition to standard care versus standard care alone. (TIF)

S82 Fig Rates of development of drug resistance in patients receiving combinations of adherence interventions in addition to standard care versus standard care alone. (TIF)

## Abbreviations

AFB: acid-fast bacilli
CS: cohort study
DOT: directly observed therapy
GRADE: Grading of Recommendations Assessment, Development, and Evaluation
HCW: healthcare worker
INH: isoniazid
M-H: Mantel-Haenszel
PICO: population, intervention, comparison, outcome
PRISMA: Preferred Reporting Items for Systematic Reviews and Meta-Analysis
RCT: randomized controlled trial
RD: risk difference
RR: risk ratio
SAT: self-administered therapy
SMS: short message service
TB: tuberculosis
VOT: video-observed therapy
WHO: World Health Organization
