# Supplementary material for: Adherence interventions and outcomes of tuberculosis treatment: A systematic review and meta-analysis of trials and observational studies
Source: PLoS Med. 2018 Jul 3;15(7):e1002595. doi: 10.1371/journal.pmed.1002595 (PMC6029765; doi:10.1371/journal.pmed.1002595)
Supplement: S2 Text — (DOC) [file pmed.1002595.s002.doc]

**Adherence Interventions in TB Treatment: A Systematic Review and Meta-Analysis**

**Study Protocol**

**OBJECTIVES**

The purpose of this study is to investigate one of the primary questions posed by the WHO expert panel of the TB Treatment Guidelines Development Committee regarding adherence interventions in TB treatment.

**Primary objective**

To determine whether in patients with active TB, any interventions to promote adherence to TB treatment are more or less likely to lead to the outcomes listed below:

- - Adherence to treatment (or treatment interruption due to non-adherence)
  - Conventional TB treatment outcomes: cured/completed, failure, relapse, survival/death, acquisition of drug resistance

**Secondary objectives**

The following sub-group analyses will be performed (if data available) to examine the impact of the following characteristics on study outcomes, both within and then across studies:

- - Disease: pulmonary vs extrapulmonary disease
  - Patients: Age (children vs adults), comorbidities such as diabetes and HIV
  - Microbes: resistance to any of the anti-TB treatment drugs
  - Treatment delivery: video assisted monitoring, medication monitors, SMS or phone call reminders, psychological/educational material

**METHODS**

**Study selection**

Inclusion criteria:

- Study design: All randomized controlled trials, case-control studies, and prospective and retrospective cohort studies
- Population of interest: patients with active tuberculosis regardless of age, drug-resistance, pulmonary or extrapulmonary disease, or HIV seropositivity. Studies on latent tuberculosis will be excluded
- Interventions: any intervention to promote treatment adherence including but not limited to
  - Supervision of treatment (DOT, VOT, etc.)
  - Reminders (medication monitors and/or SMS/phone call reminders
  - Incentives (bus tokens, food, etc)
  - Social support (education, psychological interventions)
  - Combinations of above interventions
- Comparator: studies must include an internal historical or contemporary control group. At minimum, control group should undergo routine practice: regular TB drugs pick-up and consultations with physicians or other healthcare workers are available when necessary, TB treatment is free of charge, and essential information/health education in relation to TB treatment is provided.
- Outcomes:
  - Adherence to TB treatment: Defined as completing >90% of study drugs
  - Cure: last two cultures before the end of treatment are negative
  - Failure: At least one positive culture after 6 months of treatment, or last culture positive
  - Completion: Planned treatment completed without meeting definition of cure or failure
  - Default/loss to follow up: patient stopped therapy for at least two months, more than one month before planned end of treatment, and did not meet definition of failure. This will not include patients who transferred to another health facility/program without further outcome information, or those who were not evaluated, for any reason.
  - Death: due to any cause at any time during the study period
  - Relapse: have previously been treated for TB, were declared cured or treatment completed at the end of their most recent course of treatment, and are now diagnosed with a recurrent episode of TB (either a true relapse or a new episode of TB caused by reinfection)
  - Acquired drug resistance: patients undergoing TB treatment who acquire resistance to one or more TB drugs while undergoing TB treatment

Exclusion criteria:

- Studies lacking components mentioned above
- To avoid duplicating efforts, any study of hospital vs outpatient TB treatment as the primary focus will be excluded as this question will be evaluated by a concurrent systematic review commissioned by the GDG at the time of our review.

**Data abstraction**

The following information will be collected where available:

- Name of trial/author/journal
- Year of publication
- Study setting (country)
- Study design
- Randomization process, if any
- Follow-up
- Sample size
- Characteristics of study participants (percentage of children, TB-HIV, MDR-TB, XDR-TB, EPTB)
- Type of adherence intervention
- Control group mode of TB treatment
- Study results (relative risk or risk difference) based on the outcomes of interest

**Search strategy**

Literature search will involve Medline and grey literature via searching references of published systematic reviews and other review articles as well as consulting with the international panel put together by the WHO Guidelines Development Committee. Search will be limited to English language articles with the exception of data previously abstracted from foreign language articles.

**Analysis plan**

- When two or more studies of an adherence intervention are available that report on the same outcome of interest, the results will be pooled.
- Random effects meta-analysis will be used for all estimates of effect
- All analyses will be conducted in RevMan
- The following subgroup analyses will be conducted (if data is available):
  - Children (<15 years)
  - HIV seropositive
  - Extrapulmonary TB
  - Multi-drug resistant TB
- Heterogeneity will be assessed using Chi-squared test available in RevMan with p<0.05 used to determine statistical significance.
- If >10 studies are available for an adherence intervention, funnel plots will be used to assess publication bias.
- Quality assessment
  - The Cochrane Risk of Bias tool will be used to assess the quality of all randomized controlled trials
  - The Newcastle Ottawa Scale will be used to assess the quality of cohort studies
  - The quality of evidence for each outcome of interest will be assessed using GRADE methodology

**Data management**

The initial data analysis will address only the objectives specified above. Preliminary findings from these analyses will be circulated among contributing investigators for their comments and suggestions about further analysis. Any additional analysis will be proposed to all investigators, although as long as related to the objectives above we will not seek unanimous approval of any such additional analyses. However objections to the new analysis will be addressed and resolved before proceeding. (In other words, if a collaborator does not respond, that is taken as implicit approval). If we think of analyses to address entirely different and novel objectives that are not considered or foreseen here, we will seek approval from ALL investigators before embarking on any such analyses.

Results of all analyses will be shared with all investigators at 4 stages: (1) Preliminary report to WHO of findings (March 2016); (2) GRADE profiles presented to WHO (June 2016) (3) Final report presentation to the GDG in July 2016 (4) Final analyses prior to preparation of guidelines (September 2016). These reports will be considered confidential by all involved. As such they will not be presented in any public forum, nor disseminated through any media.

All proposed publications will be reviewed and approved by all investigators before public presentation or submission for publication. The authorship will include all responsible investigators contributing data.
