# Supplementary material for: Adherence interventions and outcomes of tuberculosis treatment: A systematic review and meta-analysis of trials and observational studies
Source: PLoS Med. 2018 Jul 3;15(7):e1002595. doi: 10.1371/journal.pmed.1002595 (PMC6029765; doi:10.1371/journal.pmed.1002595)
Supplement: S1 Table — TB, tuberculosis. (DOCX) [file pmed.1002595.s003.docx]

| **Step** | **Search Terms** |
| --- | --- |
| 1 | TB |
| 2 | tuberculosis |
| 3 | 1 OR 2 |
| 4 | “directly observed therapy” |
| 5 | “directly observed treatment” |
| 6 | “supervised therapy” |
| 7 | “supervised treatment |
| 8 | DOT* |
| 9 | VOT |
| 10 | “video observed” |
| 11 | SMS |
| 12 | Text messag* |
| 13 | phone |
| 14 | telephone |
| 15 | Patient adherence |
| 16 | video |
| 17 | Patient participation |
| 18 | motivation |
| 19 | Decision support techniques |
| 20 | Default* |
| 21 | Adheren* |
| 22 | Supervis* |
| 23 | 4-22/OR |
| 24 | 3 AND 23 |
| Date conducted | 12/12/2015 |
| Results | 6394 |
| Date search repeated | 2/3/2018 |
| Final results | 7,200 |
