# Supplementary material for: Adherence interventions and outcomes of tuberculosis treatment: A systematic review and meta-analysis of trials and observational studies
Source: PLoS Med. 2018 Jul 3;15(7):e1002595. doi: 10.1371/journal.pmed.1002595 (PMC6029765; doi:10.1371/journal.pmed.1002595)
Supplement: S2 Table — SMS, short message service; TB, tuberculosis. (DOCX) [file pmed.1002595.s004.docx]

| **Step** | **Search Terms** |
| --- | --- |
| 1 | TB |
| 2 | tuberculosis |
| 3 | 1 OR 2 |
| 4 | Text message |
| 5 | SMS |
| 6 | Cell phone |
| 7 | Video |
| 8 | 4-7/OR |
| 9 | 3 AND 8 |
| Date conducted | 6/28/2016 |
| Results | 425 |
| Date search repeated | 2/3/2018 |
| Final results | 529 |
