# Supplementary material for: Adherence interventions and outcomes of tuberculosis treatment: A systematic review and meta-analysis of trials and observational studies
Source: PLoS Med. 2018 Jul 3;15(7):e1002595. doi: 10.1371/journal.pmed.1002595 (PMC6029765; doi:10.1371/journal.pmed.1002595)
Supplement: S3 Table — 1 = Newcastle-Ottawa Score provided for cohort studies. Quality of RCTs are presented separately. 2 = Study includes >50% HIV/TB patients. 3 = Study includes >50% MDR-TB patients. RCT, randomized controlled trial; TB, tuberculosis. (DOCX) [file pmed.1002595.s005.docx]

| **Reference** | **Author** | **Year** | **Study design** | **Country** | **N** | **Intervention type** | | | | | | | | | | **Quality Assessment^1^** | | |
| --- | --- | --- | --- | --- | --- | --- | --- | --- | --- | --- | --- | --- | --- | --- | --- | --- | --- | --- |
|  |  |  |  |  |  | **SAT vs. DOT** | **DOT provider type** | **DOT location** | **Patient education and counseling** | **Incentives and enablers** | **Reminders and tracers** | **Staff Education** | **Psychosocial interventions** | **Digital health interventions** | **Mixed interventions** | **Selection (4)** | **Comparability (2)** | **Outcome (3)** |
| 44 | Abassi | 2007 | Prospective | Iran | 260 | ✖ |  |  |  |  |  |  |  |  |  | 3 | 0 | 3 |
| 49 | Abuaku | 2010 | Retrospective | China | 68430 | ✖ |  |  |  |  |  |  |  |  |  | 3 | 0 | 3 |
| 78 | Akhtar | 2011 | Prospective | Pakistan | 582 |  |  | ✖ |  |  |  |  |  |  |  | 4 | 1 | 3 |
| 36 | Akkslip | 1999 | Prospective | Thailand | 779 | ✖ |  | ✖ |  |  |  |  |  |  |  | 2 | 0 | 1 |
| 111 | Al-Hajjaj | 2000 | Retrospective | Saudi Arabia | 628 |  |  |  |  |  | ✖ |  |  |  |  | 2 | 0 | 1 |
| 131 | Alvarez | 2003 | RCT | Mexico | 87 |  |  |  |  |  |  |  | ✖ |  |  |  | | |
| 52 | Alvarez-Uria^2^ | 2014 | Retrospective | India | 1460 | ✖ |  |  |  |  |  |  |  |  |  | 3 | 1 | 2 |
| 50 | Alwood^2^ | 1994 | Retrospective | USA | 78 | ✖ |  |  |  |  |  |  |  |  |  | 3 | 1 | 2 |
| 62 | Anuwatnonthakate | 2008 | Prospective | Thailand | 8031 | ✖ | ✖ |  |  |  |  |  |  |  |  | 4 | 0 | 3 |
| 79 | Arora | 2003 | Prospective | India | 2573 |  |  | ✖ |  |  |  |  |  |  |  | 4 | 0 | 2 |
| 136 | Atkins | 2011 | Prospective | South Africa | 5833 |  |  |  |  |  |  |  |  |  | ✖ | 4 | 1 | 3 |
| 37 | Balasubramanian | 2000 | Retrospective | India | 200 | ✖ |  |  |  |  |  |  |  |  |  | 3 | 0 | 3 |
| 80 | Banerjee | 2000 | Prospective | Malawi | 600 |  |  | ✖ |  |  |  |  |  |  |  | 4 | 0 | 3 |
| 10 | Baral | 2014 | RCT | Nepal | 156 |  |  |  | ✖ |  |  |  |  |  | ✖ |  | | |
| 55 | Bashar^3^ | 2001 | Retrospective | USA | 28 | ✖ |  |  |  |  |  |  |  |  |  | 4 | 1 | 3 |
| 81 | Becx-Bleumink | 2001 | Prospective | Indonesia | 2353 |  |  | ✖ |  |  |  |  |  |  |  | 4 | 0 | 3 |
| 97 | Bock | 2001 | Retrospective | USA | 107 |  |  |  |  | ✖ |  |  |  |  |  | 3 | 0 | 2 |
| 112 | Bronner | 2012 | Retrospective | South Africa | 405673 |  |  |  |  |  | ✖ |  |  |  |  | 3 | 0 | 3 |
| 124 | Broomhead | 2012 | Retrospective | South Africa | 120 |  |  |  |  |  |  |  |  | ✖ |  | 4 | 1 | 3 |
| 98 | Cantalice | 2009 | Retrospective | Brazil | 142 |  |  |  |  | ✖ |  |  |  |  |  | 4 | 0 | 3 |
| 60 | Cavalcante | 2007 | Retrospective | Brazil | 1811 | ✖ |  | ✖ |  |  |  |  |  |  |  | 3 | 0 | 3 |
| 59 | Cayla | 2004 | Prospective | Spain | 1515 | ✖ |  |  |  |  |  |  |  |  |  | 4 | 1 | 3 |
| 46 | Cayla | 2009 | Prospective | Spain | 1490 | ✖ |  |  |  |  |  |  |  |  |  | 4 | 2 | 3 |
| 137 | Chan | 2013 | Retrospective | Taiwan | 390 |  |  |  |  |  |  |  |  |  | ✖ | 4 | 2 | 3 |
| 31 | Chennai | 1997 | RCT | India | 825 | ✖ |  |  |  |  |  |  |  |  |  |  | | |
| 69 | Chien | 2013 | Retrospective | Taiwan | 2160 | ✖ |  |  |  |  |  |  |  |  |  | 4 | 0 | 3 |
| 99 | Chua | 2015 | Retrospective | Singapore | 883 |  |  |  |  | ✖ |  |  |  |  |  | 4 | 1 | 3 |
| 134 | Chuck | 2016 | Prospective | USA | 390 |  |  |  |  |  |  |  |  | ✖ |  | 4 | 0 | 3 |
| 67 | Chung | 2007 | Retrospective | Taiwan | 399 | ✖ |  |  |  |  |  |  |  |  |  | 2 | 2 | 3 |
| 7 | Clark | 2007 | RCT | Turkey | 114 |  |  |  | ✖ |  |  |  |  |  |  |  | | |
| 42 | Daniel | 2006 | Retrospective | Nigeria | 467 | ✖ |  |  |  |  |  |  |  |  |  | 3 | 0 | 3 |
| 51 | Das | 2014 | Retrospective | India | 89 | ✖ |  |  |  |  |  |  |  |  |  | 3 | 0 | 3 |
| 128 | Datiko | 2009 | RCT | Ethiopia | 318 |  |  |  |  |  |  | ✖ |  |  |  |  | | |
| 74 | Dave | 2016 | RCT | India | 624 |  | ✖ |  |  |  |  |  |  |  |  |  |  |  |
| 138 | Davidson | 1998 | Retrospective | USA | 319 |  |  |  |  |  |  |  |  |  | ✖ | 4 | 1 | 3 |
| 132 | Demissie | 2003 | Prospective | Ethiopia | 128 |  |  |  |  |  |  |  | ✖ |  |  | 3 | 0 | 3 |
| 11 | Dick | 1997 | Prospective | South Africa | 120 |  |  |  | ✖ |  |  |  |  |  |  | 3 | 0 | 2 |
| 82 | Dobler | 2015 | Retrospective | Mongolia | 2181 |  |  | ✖ |  | ✖ |  |  |  |  |  | 3 | 0 | 3 |
| 143 | Drabo | 2009 | RCT | Burkina Faso | 333 |  |  |  |  |  |  |  |  |  | ✖ |  | | |
| 148 | Duarte | 2011 | Retrospective | Portugal | 141 |  |  |  |  |  |  |  |  |  | ✖ | 4 | 0 | 2 |
| 83 | Dudley | 2003 | Prospective | South Africa | 2873 |  |  | ✖ |  |  |  |  |  |  |  | 4 | 1 | 3 |
| 54 | Ershova^2^ | 2014 | Retrospective | South Africa | 741 | ✖ |  |  |  |  |  |  |  |  |  | 3 | 0 | 3 |
| 133 | Fang | 2017 | RCT | China | 350 |  |  |  |  |  |  |  |  | ✖ |  |  | | |
| 139 | Farmer | 1991 | Prospective | Haiti | 60 |  |  |  |  |  |  |  |  |  | ✖ | 2 | 0 | 3 |
| 140 | Garden | 2012 | Prospective | Russia | 518 |  |  |  |  |  |  |  |  |  | ✖ | 1 | 0 | 3 |
| 113 | Hermans | 2017 | Quasi-RCT | Uganda | 183 |  |  |  |  |  | ✖ |  |  | ✖ |  | 4 | 1 | 3 |
| 144 | Hsieh | 2008 | RCT | Taiwan | 96 |  |  |  |  |  |  |  |  |  | ✖ |  | | |
| 116 | Iribarren | 2013 | RCT | Argentina | 37 |  |  |  |  |  | ✖ |  |  | ✖ |  |  |  |  |
| 107 | Jahnavi | 2010 | RCT | India | 100 |  |  |  |  | ✖ |  |  |  |  |  |  |  |  |
| 8 | Janmeja | 2004 | RCT | India | 200 |  |  |  | ✖ |  |  |  |  |  |  |  |  |  |
| 58 | Jasmer | 2004 | Retrospective | USA | 372 | ✖ |  |  |  |  |  |  |  |  | ✖ | 3 | 1 | 2 |
| 53 | Juan^2^ | 2006 | Mixed | Spain | 213 | ✖ |  |  |  |  |  |  |  |  |  | 4 | 1 | 2 |
| 29 | Kamolratanakul | 1999 | RCT | Thailand | 836 | ✖ |  |  |  |  |  |  |  |  |  |  | | |
| 63 | Kapella | 2009 | Retrospective | Thailand | 791 | ✖ |  |  |  |  |  |  |  |  |  | 4 | 0 | 1 |
| 145 | Khortwong | 2013 | Quasi-RCT | Thailand | 100 |  |  |  |  |  |  |  |  |  | ✖ |  | | |
| 75 | Kingkaew | 2008 | Prospective | Thailand | 506 |  | ✖ |  |  |  |  |  |  |  |  | 4 | 1 | 2 |
| 84 | Kironde | 2002 | Prospective | South Africa | 505 |  |  | ✖ |  |  |  |  |  |  |  | 4 | 0 | 3 |
| 117 | Krishnaswami | 1981 | RCT | South India | 150 |  |  |  |  |  | ✖ |  |  |  |  |  | | |
| 118 | Kunawarak | 2011 | RCT | Thailand | 61 |  |  |  |  |  | ✖ |  |  | ✖ |  |  |  |  |
| 72 | Lei | 2016 | Prospective | China | 481 | ✖ |  | ✖ |  |  |  |  |  |  |  | 4 | 1 | 3 |
| 126 | Lewin | 2005 | RCT | South Africa | 1177 |  |  |  |  |  |  | ✖ |  |  |  |  | | |
| 9 | Liefooghe | 1999 | RCT | Pakistan | 1019 |  |  |  | ✖ |  |  |  |  |  |  |  |  |  |
| 17 | Liu | 2015 | RCT | China | 4173 |  |  |  |  |  |  |  |  | ✖ |  |  |  |  |
| 100 | Lu | 2013 | Prospective | China | 2006 |  |  |  |  | ✖ |  |  |  |  |  | 3 | 1 | 2 |
| 108 | Lutge | 2013 | RCT | South Africa | 4,091 |  |  |  |  | ✖ |  |  |  |  |  |  | | |
| 93 | Lwilla | 2003 | RCT | Tanzania | 522 |  |  | ✖ |  |  |  |  |  |  |  |  |  |  |
| 66 | Mac | 1999 | Retrospective | USA | 50 | ✖ |  |  |  |  |  |  |  |  |  | 4 | 2 | 3 |
| 85 | Maciel | 2010 | Prospective | Brazil | 171 |  |  | ✖ |  |  |  |  |  |  |  | 4 | 0 | 3 |
| 30 | Macintyre | 2003 | Quasi-RCT | Australia | 173 | ✖ |  |  |  |  |  |  |  |  |  |  | | |
| 149 | Macq | 2008 | Prospective | Nicaragua | 268 |  |  |  |  |  |  |  |  |  | ✖ | 4 | 1 | 3 |
| 86 | Manders | 2001 | Prospective | Malawi | 75 |  |  | ✖ |  |  |  |  |  |  |  | 4 | 0 | 3 |
| 109 | Martins | 2009 | RCT | East Timor | 270 |  |  |  |  | ✖ |  |  |  |  |  |  | | |
| 38 | Mathema | 2001 | Prospective | Nepal | 759 | ✖ |  | ✖ |  |  |  |  |  |  |  | 4 | 0 | 2 |
| 87 | Mhimbira | 2016 | Retrospective | Tanzania | 4835 |  |  | X |  |  |  |  |  |  |  | 4 | 1 | 3 |
| 88 | Miti | 2003 | Prospective | Zambia | 168 |  |  | ✖ |  |  |  |  |  |  |  | 3 | 0 | 2 |
| 89 | Moalosi | 2003 | Retrospective | Botswana | 633 |  |  | ✖ |  |  |  |  |  |  |  | 0 | 0 | 2 |
| 119 | Mohammed | 2016 | RCT | Pakistan | 2207 |  |  |  |  |  | ✖ |  |  | ✖ |  |  | | |
| 120 | Mohan | 2003 | RCT | Iraq | 480 |  |  |  |  |  | ✖ |  |  |  |  |  |  |  |
| 71 | Mohr^2,3^ | 2017 | Prospective | South Africa | 404 | ✖ |  |  |  |  |  |  |  |  |  | 4 | 1 | 3 |
| 146 | Morisky | 1990 | RCT | USA | 88 |  |  |  |  |  |  |  |  |  | ✖ |  | | |
| 121 | Moulding | 2002 | RCT | Haiti | 2002 |  |  |  |  |  | ✖ |  |  |  |  |  |  |  |
| 94 | Newell | 2006 | RCT | Nepal | 907 |  |  | ✖ |  |  |  |  |  |  |  |  |  |  |
| 101 | Ngamvithayapong-Yanai | 2013 | Retrospective | Thailand | 759 |  |  |  |  | ✖ |  |  |  |  |  | 4 | 0 | 2 |
| 90 | Niazi | 2003 | Prospective | Iraq | 172 |  |  | ✖ |  |  |  |  |  |  |  | 4 | 1 | 2 |
| 41 | Nirupa | 2005 | Retrospective | India | 865 | ✖ | ✖ | ✖ |  |  |  |  |  |  |  | 4 | 0 | 3 |
| 43 | Okanurak | 2007 | Prospective | Thailand | 931 | ✖ |  |  |  |  |  |  |  |  |  | 4 | 1 | 3 |
| 56 | Olle-Goig^2^ | 2001 | Retrospective | Haiti | 281 | ✖ |  |  |  |  |  |  |  |  |  | 4 | 0 | 3 |
| 65 | Ong’ang’o | 2014 | Retrospective | Kenya | 2778 | ✖ |  |  |  |  |  |  |  |  |  | 3 | 0 | 3 |
| 5 | Ormerod | 2002 | Mixed | UK | 205 | ✖ |  |  |  |  |  |  |  |  |  | 2 | 0 | 2 |
| 122 | Paramasivan | 1993 | RCT | India | 200 |  |  |  |  |  | ✖ |  |  |  |  |  | | |
| 127 | Puchalski Ritchie | 2015 | RCT | Malawi | 178 |  |  |  |  |  |  | ✖ |  |  |  |  |  |  |
| 57 | Pungrassami | 2002 | Prospective | Thailand | 411 | ✖ |  |  |  |  |  |  |  |  |  | 4 | 1 | 3 |
| 61 | Radilla-Chavez | 2007 | Retrospective | Mexico | 629 | ✖ |  |  |  |  |  |  |  |  |  | 4 | 0 | 2 |
| 129 | Safdar | 2011 | Prospective | Pakistan | 194 |  |  |  |  |  |  | ✖ |  |  |  | 3 | 0 | 2 |
| 130 | Shin | 2013 | RCT | Russia | 196 |  |  |  |  |  |  |  | ✖ |  |  |  | | |
| 76 | Singh | 2004 | Retrospective | India | 617 |  | ✖ |  |  |  |  |  |  |  |  | 4 | 0 | 3 |
| 114 | Snidal | 2015 | Prospective | Uganda | 142 |  |  |  |  |  | ✖ |  |  |  |  | 4 | 1 | 3 |
| 73 | Snyder | 2016 | Retrospective | Brazil | 6601 | ✖ |  |  |  |  |  |  |  |  |  | 4 | 1 | 3 |
| 141 | Soares | 2013 | Prospective | Brazil | 2623 |  |  |  |  |  |  |  |  |  | ✖ | 3 | 0 | 2 |
| 102 | Sripad | 2014 | Mixed | Ecuador | 191 |  |  |  |  | ✖ |  |  |  |  |  | 3 | 0 | 1 |
| 110 | Sudarsanam | 2011 | RCT | India | 97 |  |  |  |  | ✖ |  |  |  |  |  |  | | |
| 45 | Szczesniak | 2009 | Retrospective | Poland | 100 | ✖ |  |  |  |  |  |  |  |  |  | 4 | 1 | 3 |
| 35 | Tandon | 2002 | RCT | India | 400 | ✖ |  |  |  |  |  |  |  |  |  |  | | |
| 123 | Tanke | 1994 | Quasi-RCT | USA | 2008 |  |  |  |  |  | ✖ |  |  |  |  |  |  |  |
| 147 | Thiam | 2007 | RCT | Senegal | 1522 |  |  |  |  |  |  |  |  |  | ✖ |  |  |  |
| 115 | Thomson | 2011 | Retrospective | Kenya | 1369 |  |  |  |  |  | ✖ |  |  |  |  | 4 | 1 | 2 |
| 151 | Tola | 2016 | RCT | Ethiopia | 698 |  |  |  |  |  |  |  |  |  | ✖ |  | | |
| 103 | Torrens | 2016 | Retrospective | Brazil | 7255 |  |  |  |  | ✖ |  |  |  |  |  | 4 | 1 | 3 |
| 91 | Tripathy | 2013 | Retrospective | India | 1769 |  |  | ✖ |  |  |  |  |  |  |  | 4 | 0 | 2 |
| 104 | Tsai | 2010 | Retrospective | Taiwan | 17061 |  |  |  |  | ✖ |  |  |  |  |  | 2 | 2 | 3 |
| 40 | Tsuchida | 2003 | Retrospective | Japan | 80 | ✖ |  |  |  |  |  |  |  |  |  | 4 | 2 | 3 |
| 92 | Van den Boogaard | 2009 | Retrospective | Tanzania | 2769 |  |  | ✖ |  |  |  |  |  |  |  | 1 | 2 | 3 |
| 64 | Vieira | 2011 | Retrospective | Brazil | 218 | ✖ |  |  |  |  |  |  |  |  |  | 4 | 1 | 3 |
| 135 | Wade | 2012 | Retrospective | Australia | 128 |  |  |  |  |  |  |  |  | ✖ |  | 3 | 0 | 3 |
| 150 | Wai | 2017 | Retrospective | Myanmar | 261 |  |  |  |  |  |  |  |  |  | ✖ | 4 | 1 | 2 |
| 32 | Walley | 2001 | RCT | Pakistan | 497 | ✖ |  |  |  |  |  |  |  |  |  |  | | |
| 95 | Wandwalo | 2004 | RCT | Tanzania | 587 |  |  | ✖ |  |  |  |  |  |  |  |  |  |  |
| 105 | Wei | 2012 | Prospective | China | 183 |  |  |  |  | ✖ |  |  |  |  |  | 1 | 0 | 3 |
| 4 | Weis | 1994 | Retrospective | USA | 988 | ✖ |  |  |  |  |  |  |  |  |  | 4 | 0 | 3 |
| 77 | Wilkinson | 1997 | Retrospective | South Africa | 1890 |  | ✖ |  |  |  |  |  |  |  |  | 2 | 0 | 2 |
| 96 | Wright | 2004 | RCT | Swaziland | 1353 |  |  | ✖ |  |  |  |  |  |  |  |  | | |
| 48 | Xu | 2009 | Prospective | China | 670 | ✖ | ✖ | ✖ |  |  |  |  |  |  |  | 4 | 2 | 3 |
| 142 | Yassin | 2013 | Prospective | Ethiopia | 5090 |  |  |  |  |  |  |  |  |  | ✖ | 4 | 2 | 3 |
| 68 | Yen | 2013 | Retrospective | Taiwan | 3487 | ✖ |  |  |  |  |  |  |  |  |  | 4 | 2 | 3 |
| 70 | Yen | 2017 | Retrospective | Taiwan | 5011 | ✖ |  |  |  |  |  |  |  |  |  | 4 | 1 | 3 |
| 106 | Zou | 2013 | Prospective | China | 787 |  |  |  |  | ✖ |  |  |  |  |  | 3 | 0 | 3 |
| 47 | Zvavamwe | 2009 | Prospective | Namibia | 332 | ✖ |  |  |  |  |  |  |  |  |  | 0 | 0 | 3 |
| 33 | Zwarenstein | 1998 | RCT | South Africa | 216 | ✖ |  |  |  |  |  |  |  |  |  |  | | |
| 34 | Zwarenstein | 2000 | RCT | South Africa | 156 | ✖ |  |  |  |  |  |  |  |  |  |  |  |  |
