# Supplementary figures and images for: Adherence interventions and outcomes of tuberculosis treatment: A systematic review and meta-analysis of trials and observational studies
Source: PLoS Med. 2018 Jul 3;15(7):e1002595. doi: 10.1371/journal.pmed.1002595 (PMC6029765; doi:10.1371/journal.pmed.1002595)

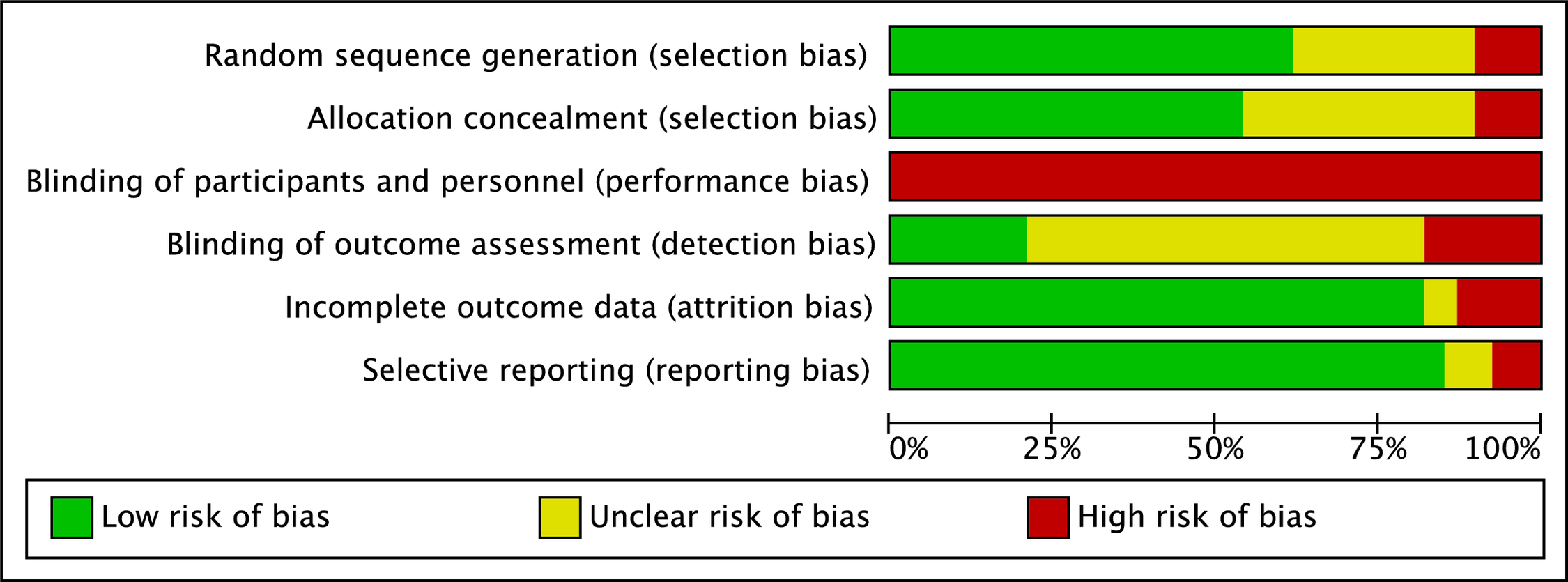

Supplement: S1 Fig — (TIF) [file pmed.1002595.s006.tif]

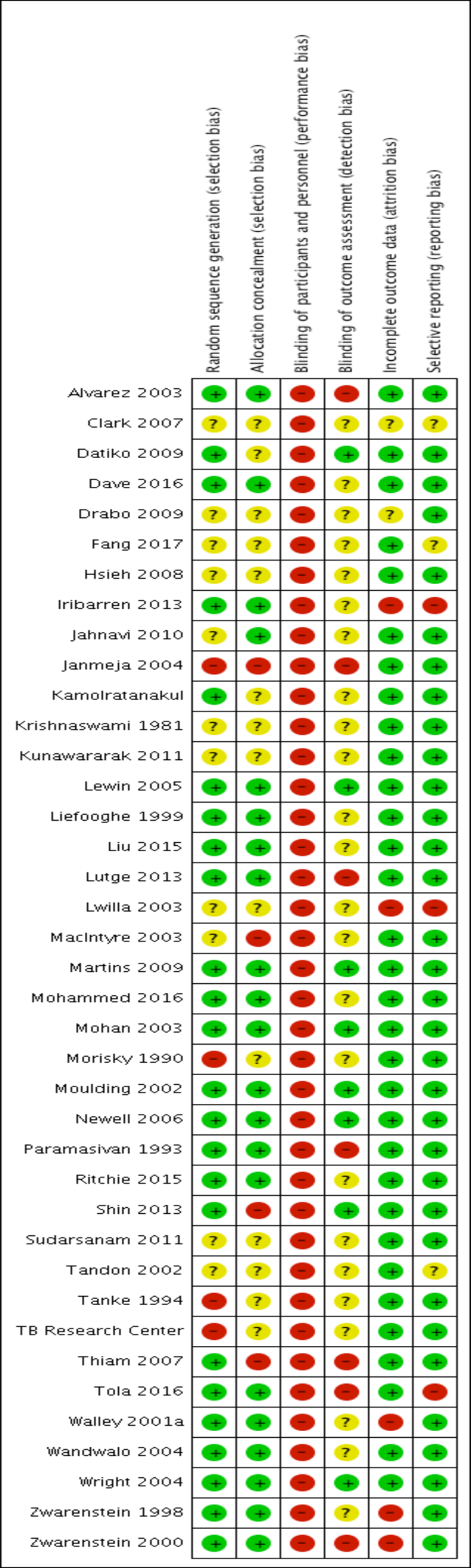

Supplement: S2 Fig — (TIF) [file pmed.1002595.s007.tif]

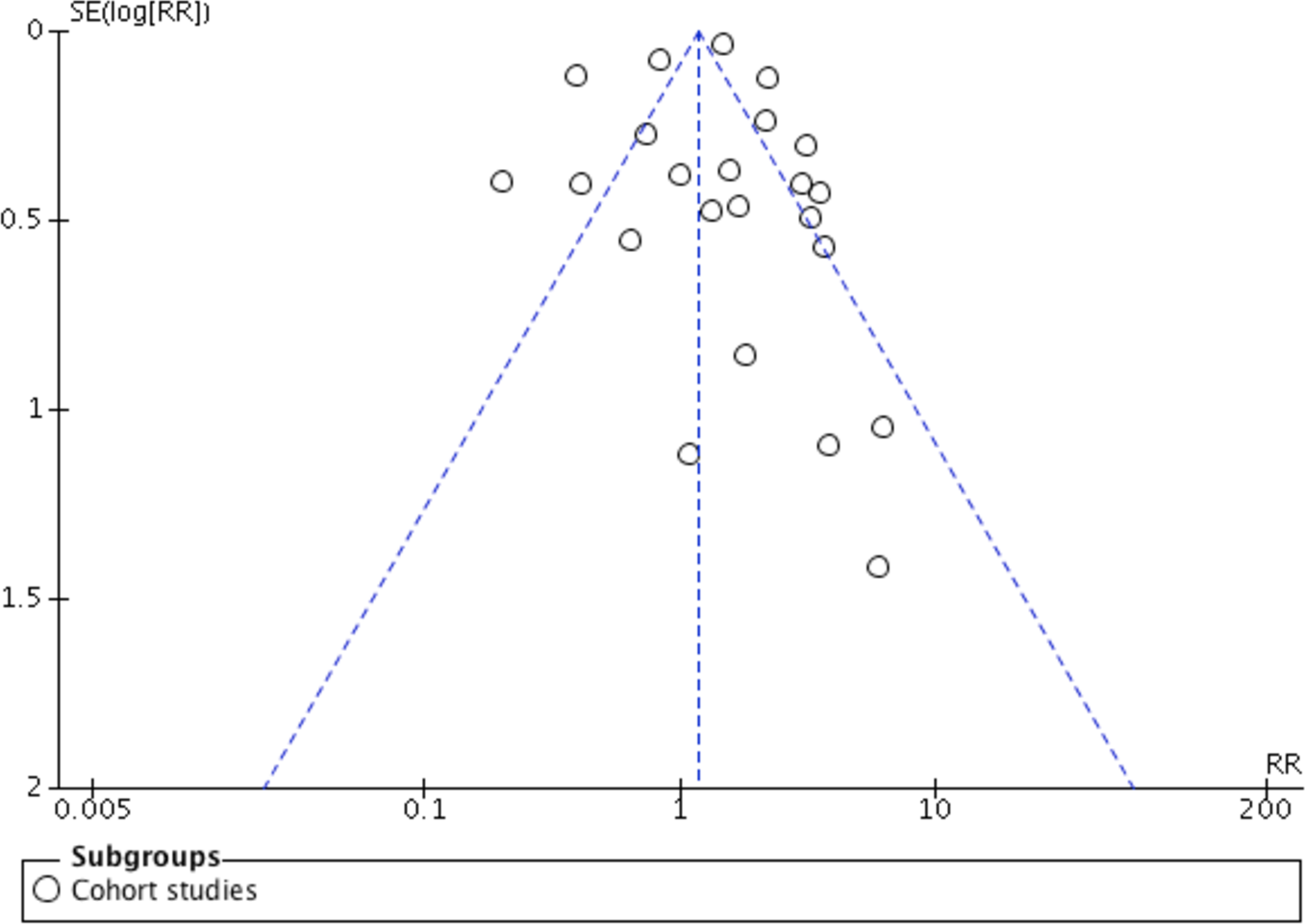

Supplement: S3 Fig — DOT, directly observed therapy; SAT, self-administered therapy. (TIF) [file pmed.1002595.s008.tif]

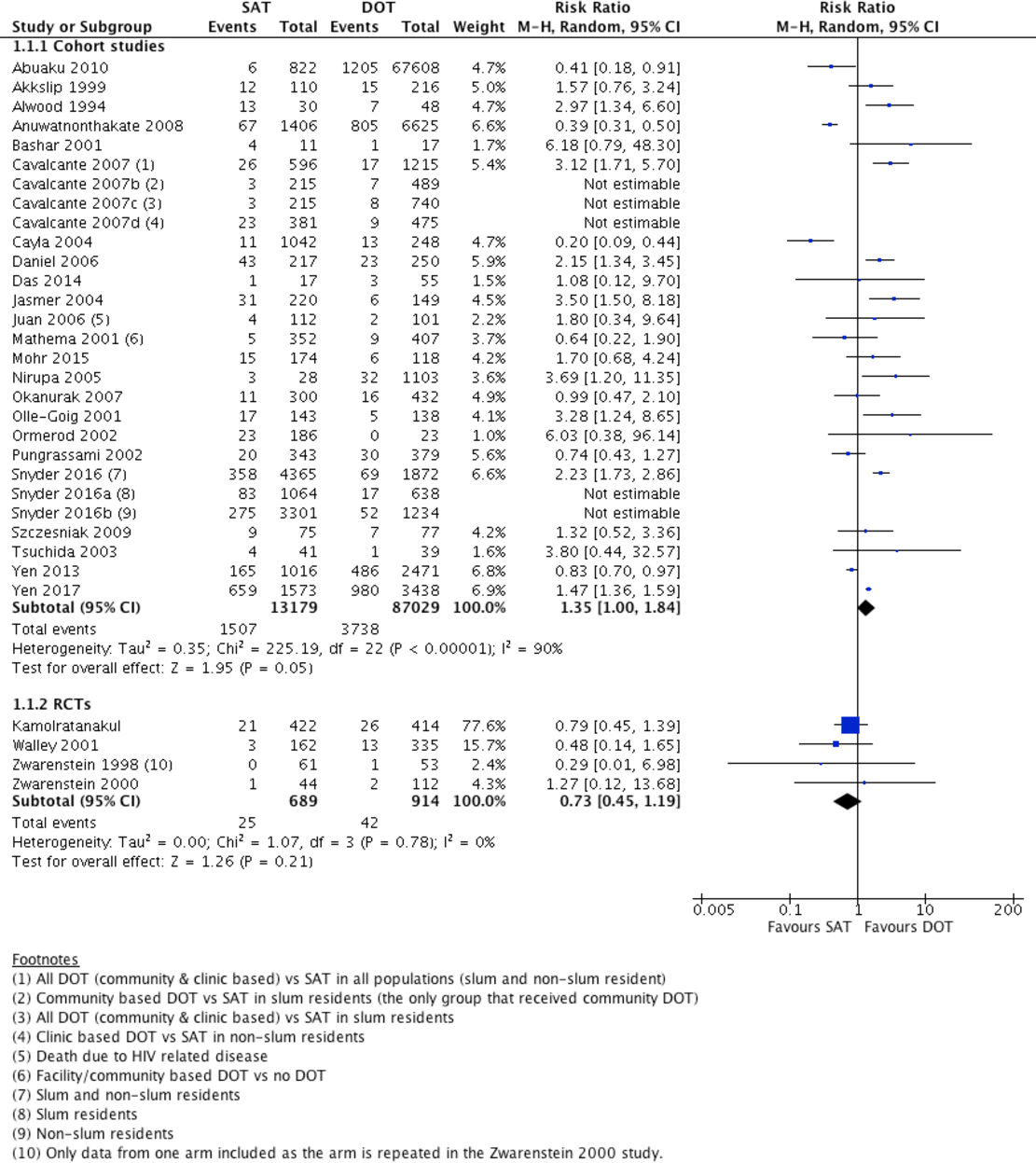

Supplement: S4 Fig — “Not estimable” denotes a subgroup within a study not included in the meta-analysis. DOT, directly observed therapy; SAT, self-administered therapy. (TIF) [file pmed.1002595.s009.tif]

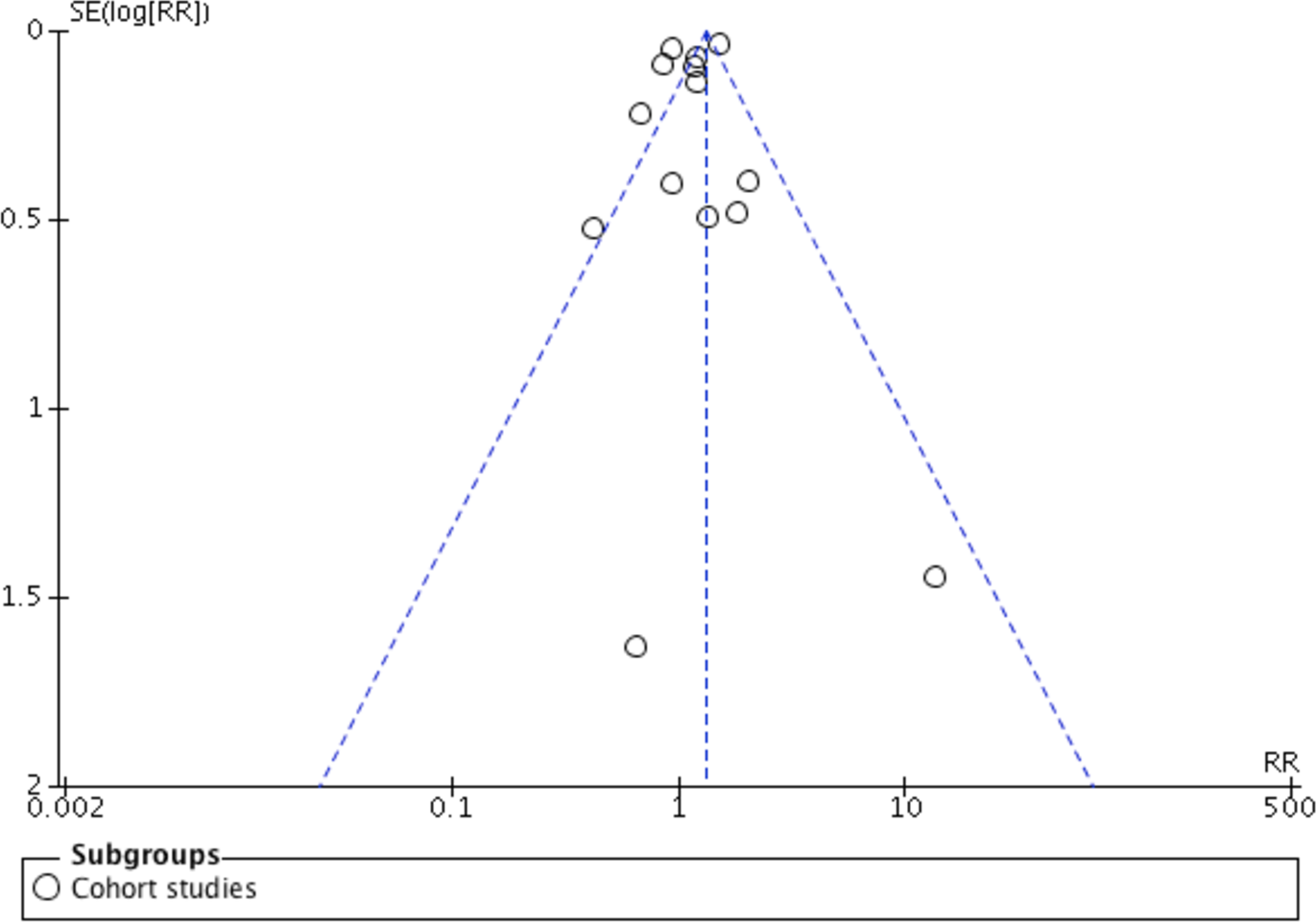

Supplement: S5 Fig — DOT, directly observed therapy; SAT, self-administered therapy. (TIF) [file pmed.1002595.s010.tif]

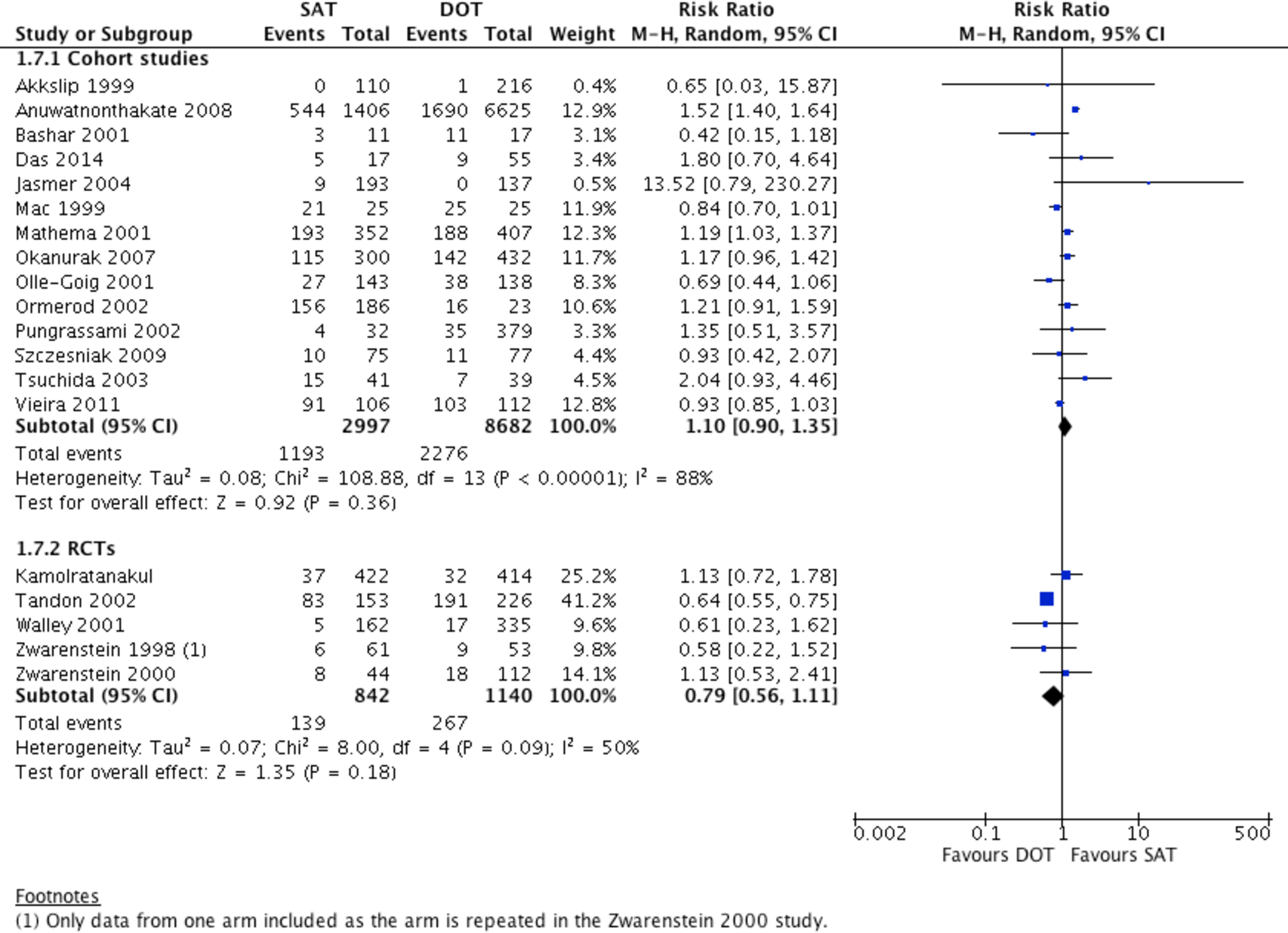

Supplement: S6 Fig — “Not estimable” denotes a subgroup within a study not included in the meta-analysis. DOT, directly observed therapy; SAT, self-administered therapy. (TIF) [file pmed.1002595.s011.tif]

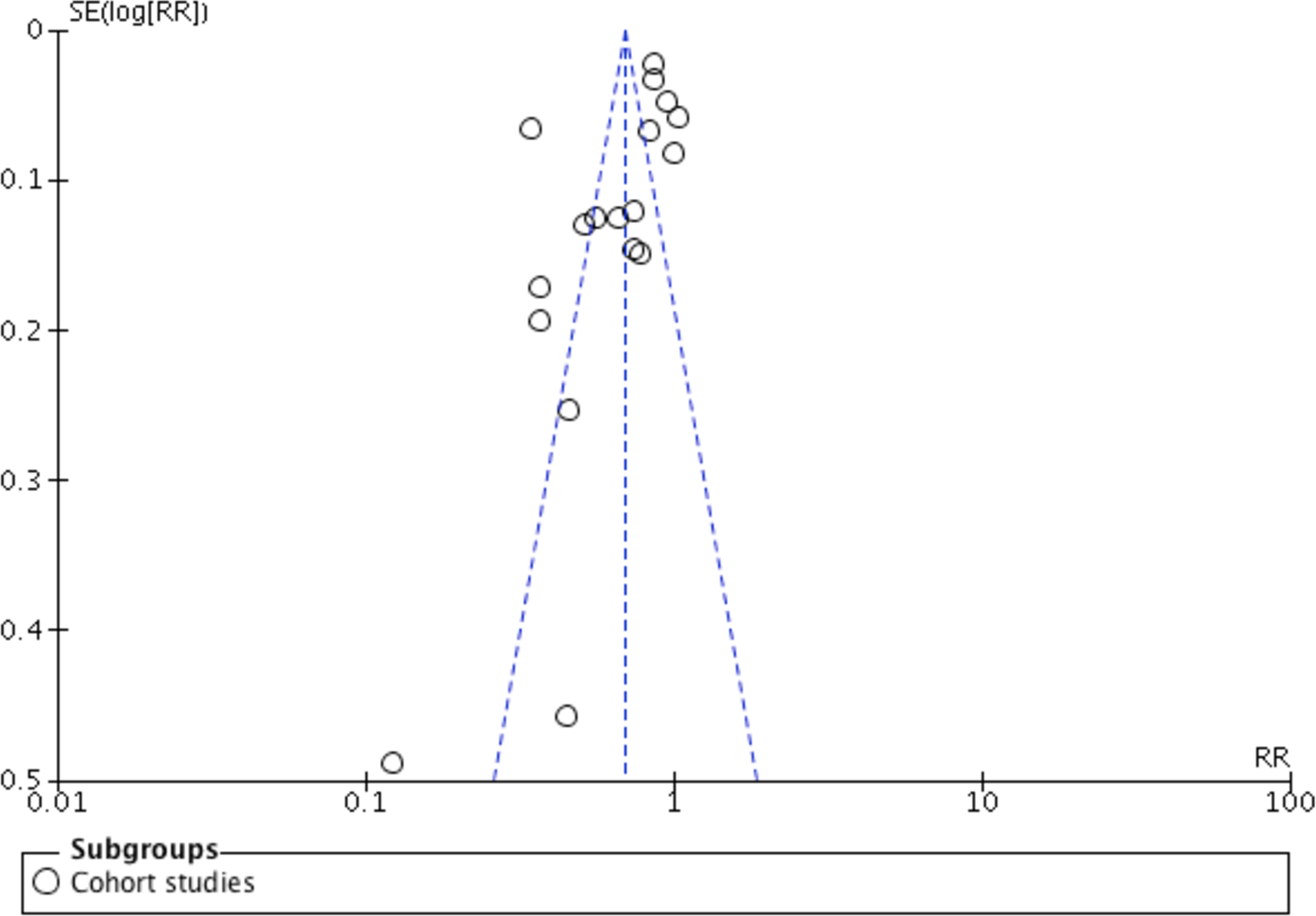

Supplement: S7 Fig — DOT, directly observed therapy; SAT, self-administered therapy. (TIF) [file pmed.1002595.s012.tif]

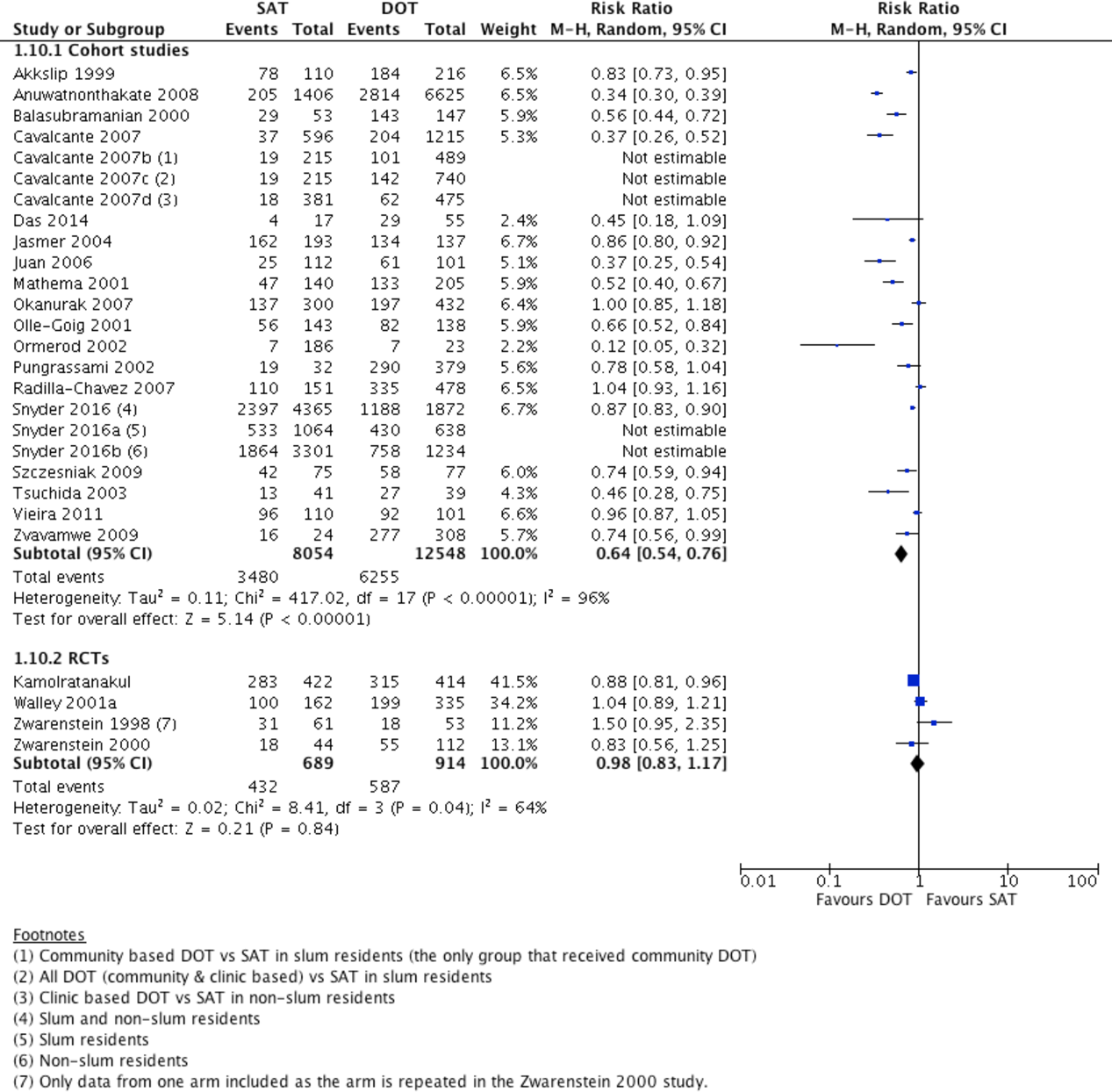

Supplement: S8 Fig — “Not estimable” denotes a subgroup within a study not included in the meta-analysis. DOT, directly observed therapy; SAT, self-administered therapy. (TIF) [file pmed.1002595.s013.tif]

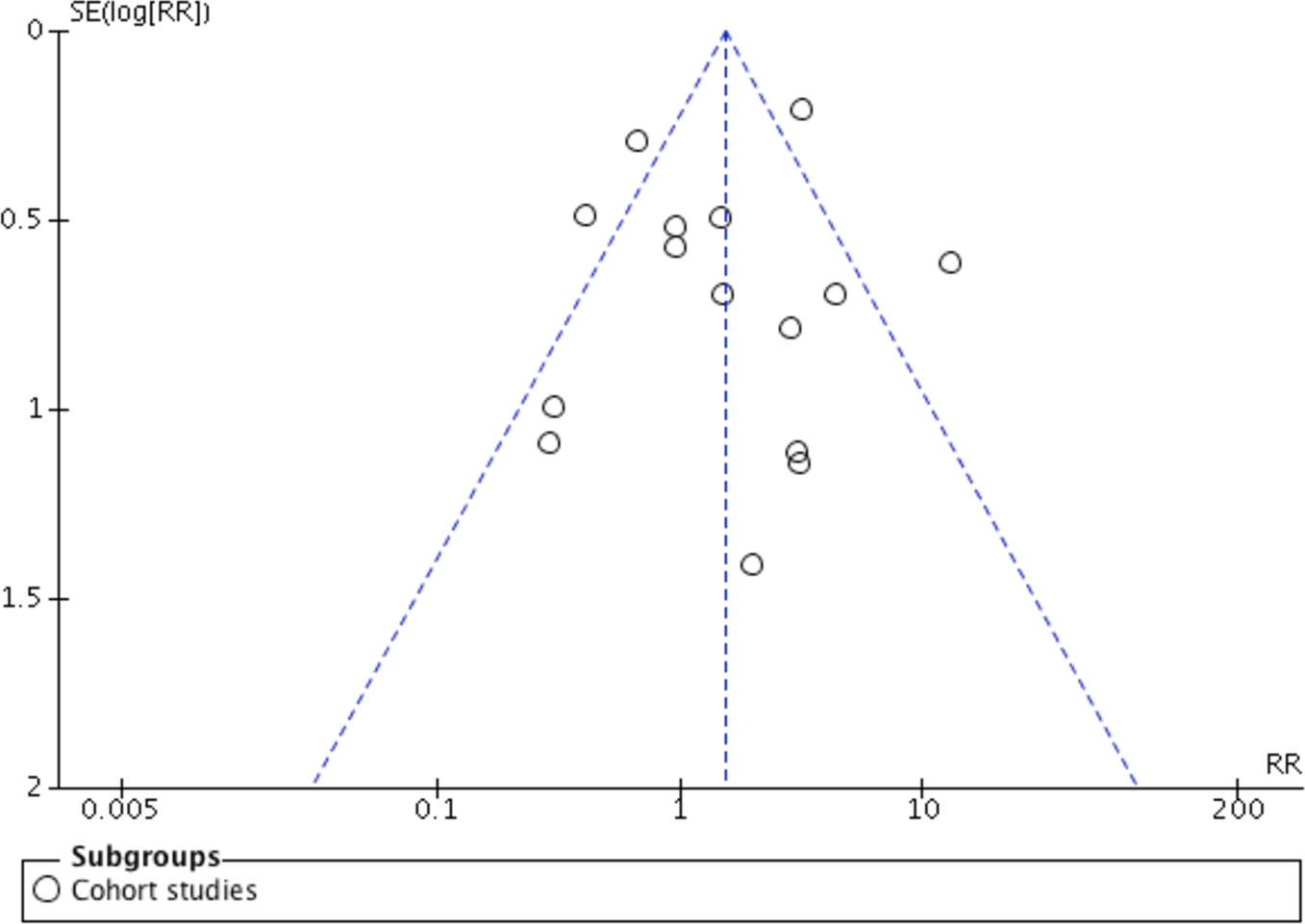

Supplement: S9 Fig — DOT, directly observed therapy; SAT, self-administered therapy. (TIF) [file pmed.1002595.s014.tif]

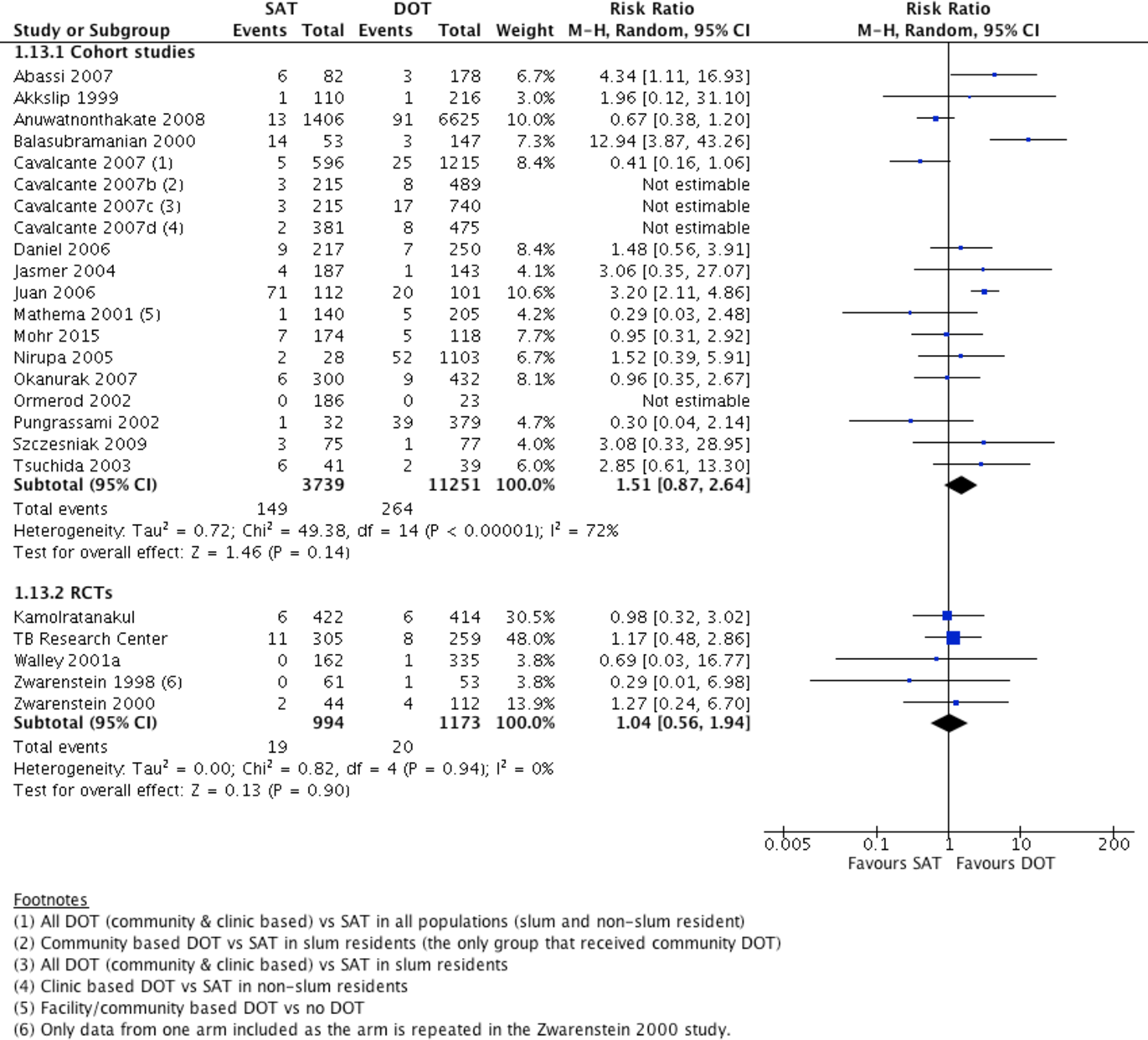

Supplement: S10 Fig — “Not estimable” denotes a subgroup within a study not included in the meta-analysis. DOT, directly observed therapy; SAT, self-administered therapy. (TIF) [file pmed.1002595.s015.tif]

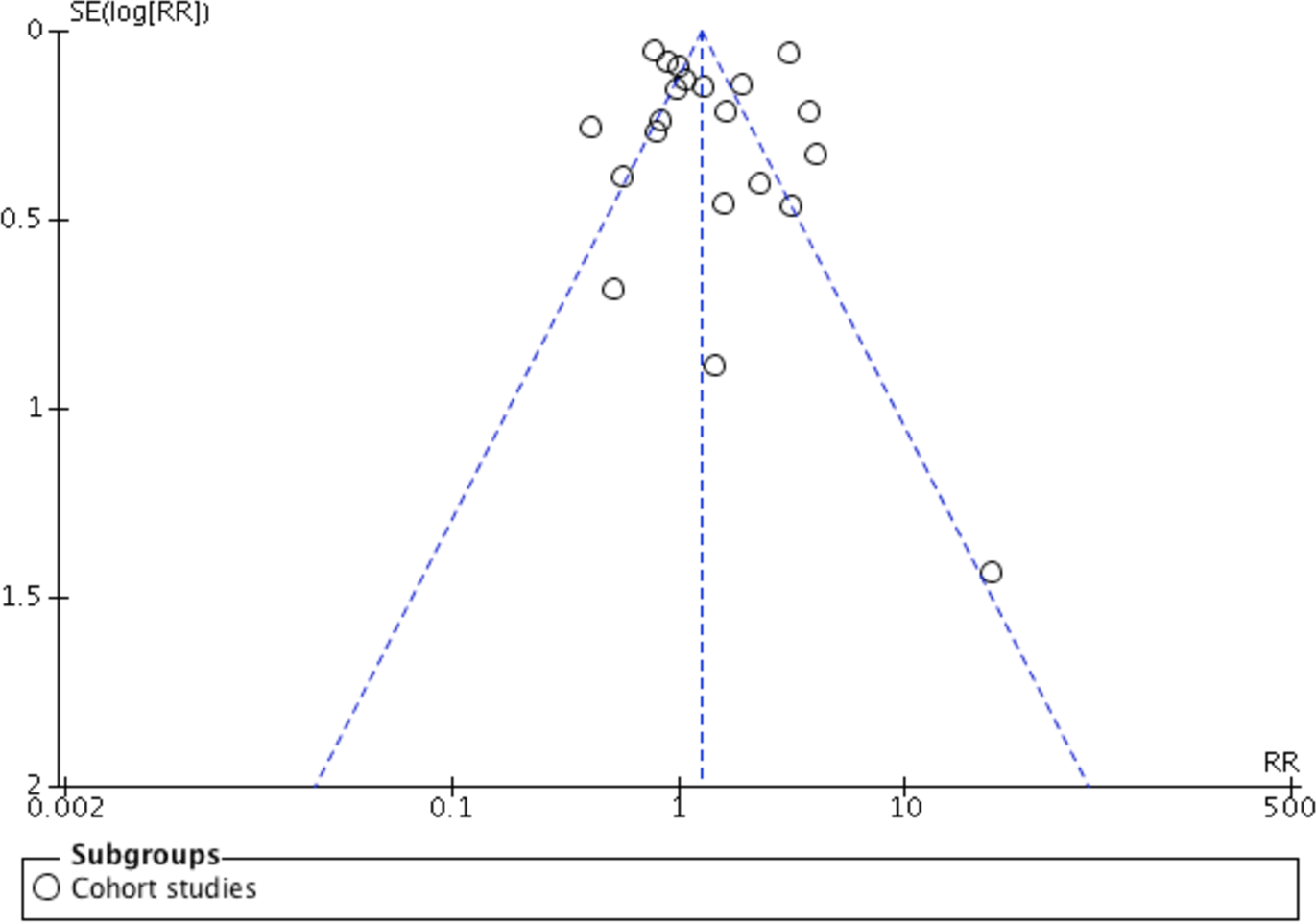

Supplement: S11 Fig — DOT, directly observed therapy; SAT, self-administered therapy. (TIF) [file pmed.1002595.s016.tif]

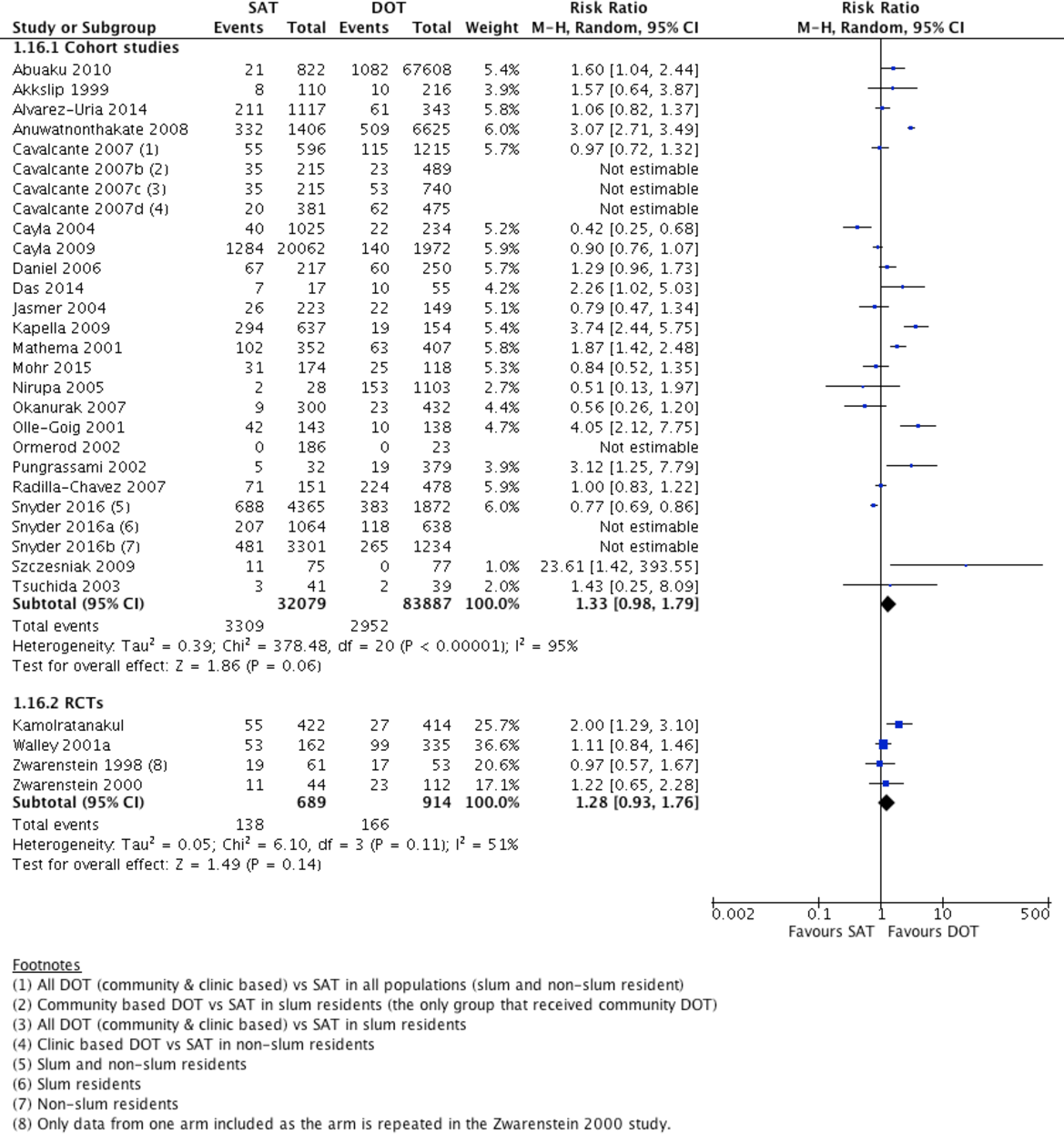

Supplement: S12 Fig — “Not estimable” denotes a subgroup within a study not included in the meta-analysis. DOT, directly observed therapy; SAT, self-administered therapy. (TIF) [file pmed.1002595.s017.tif]

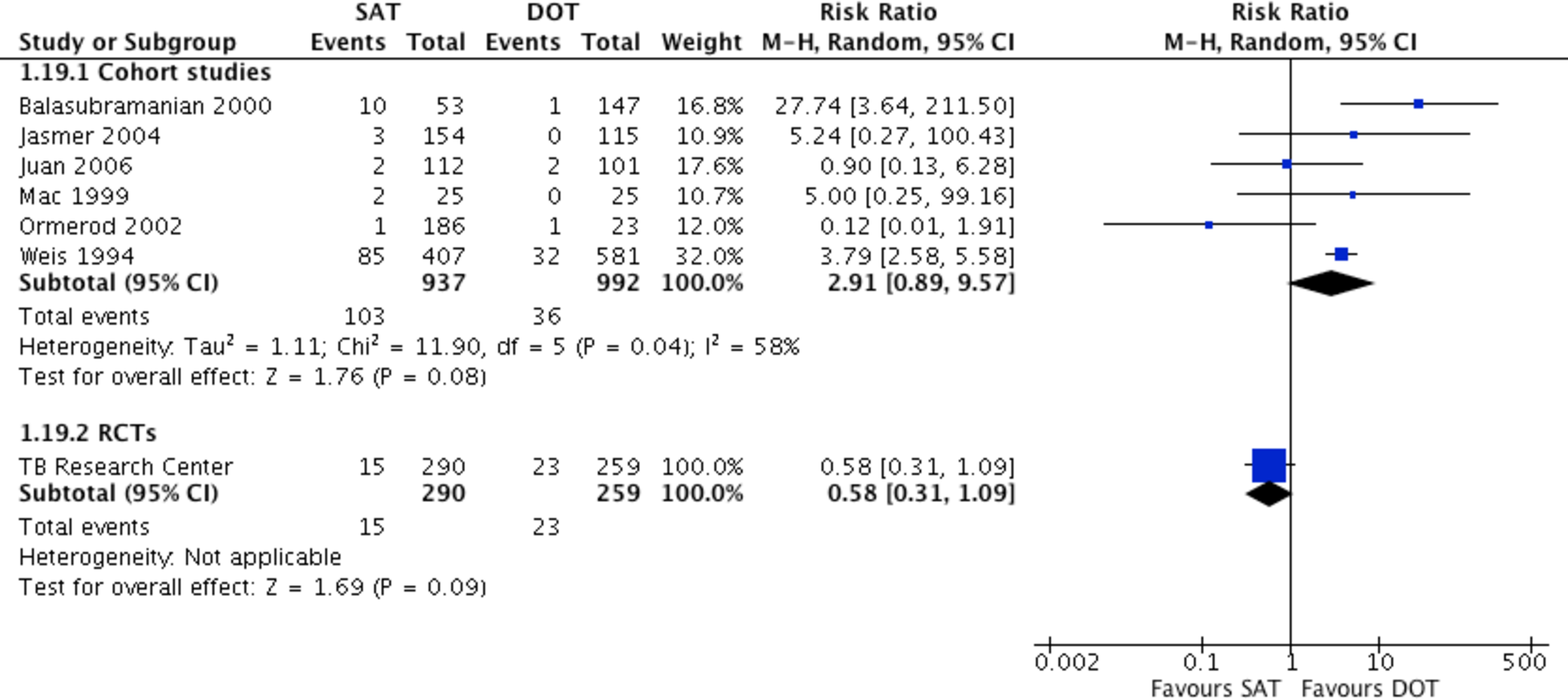

Supplement: S13 Fig — DOT, directly observed therapy; SAT, self-administered therapy. (TIF) [file pmed.1002595.s018.tif]

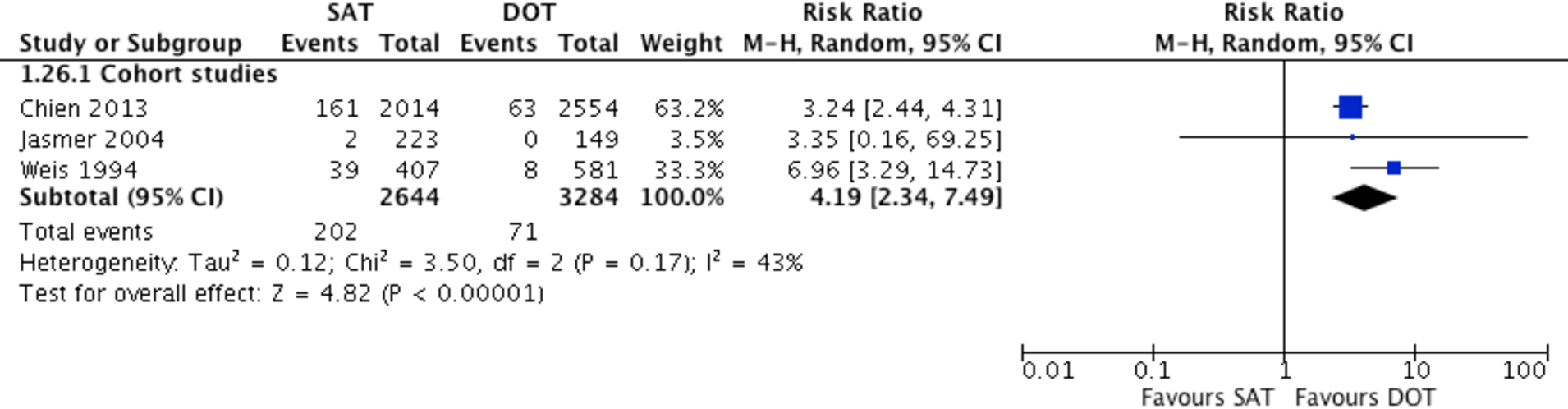

Supplement: S14 Fig — DOT, directly observed therapy; SAT, self-administered therapy. (TIF) [file pmed.1002595.s019.tif]

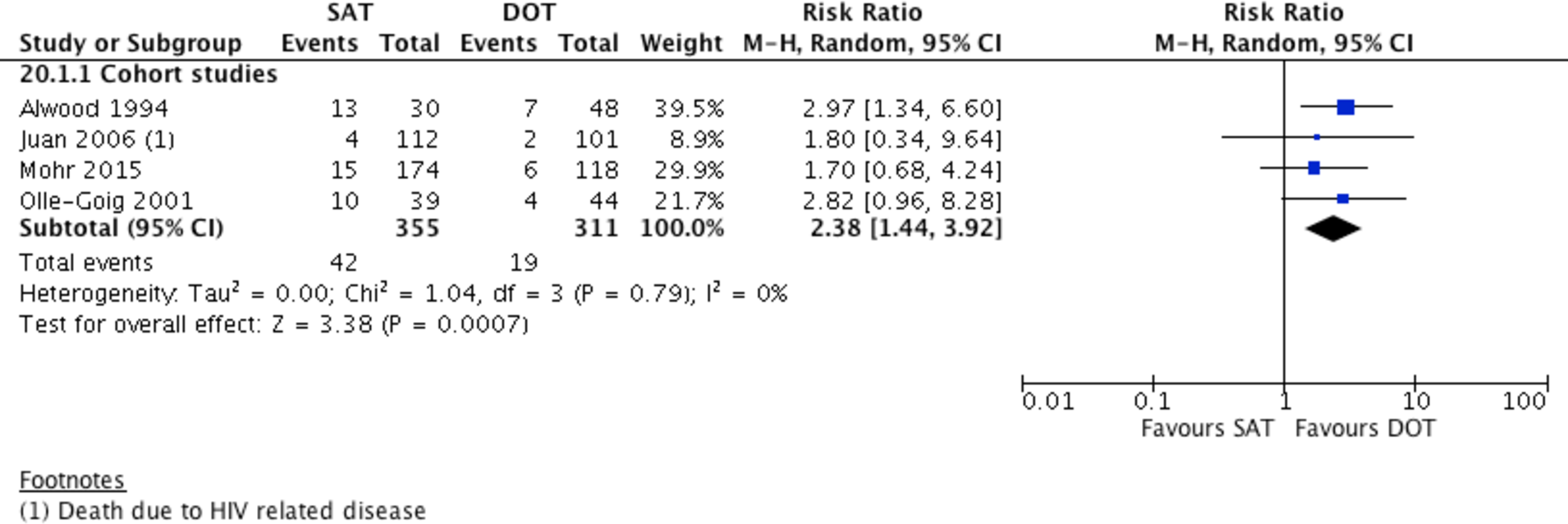

Supplement: S15 Fig — DOT, directly observed therapy; SAT, self-administered therapy; TB, tuberculosis. (TIF) [file pmed.1002595.s020.tif]

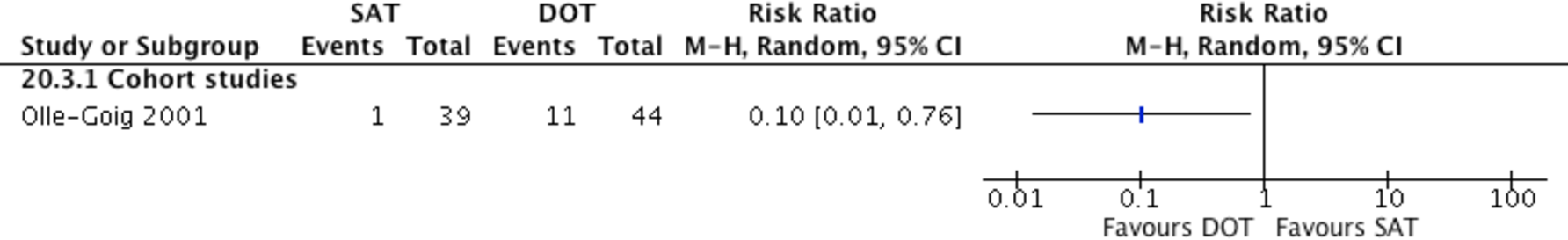

Supplement: S16 Fig — DOT, directly observed therapy; SAT, self-administered therapy; TB, tuberculosis. (TIF) [file pmed.1002595.s021.tif]

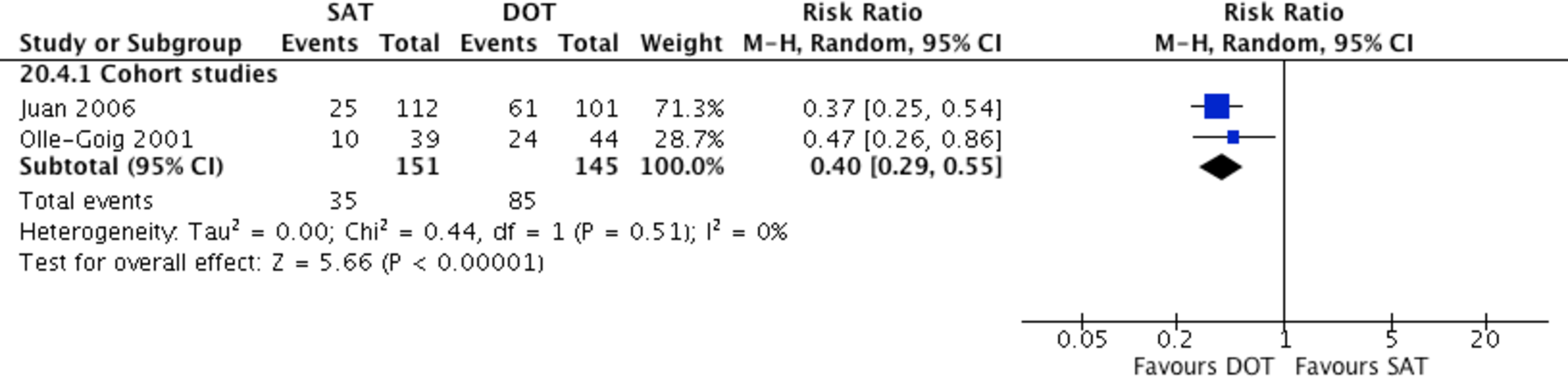

Supplement: S17 Fig — DOT, directly observed therapy; SAT, self-administered therapy; TB, tuberculosis. (TIF) [file pmed.1002595.s022.tif]

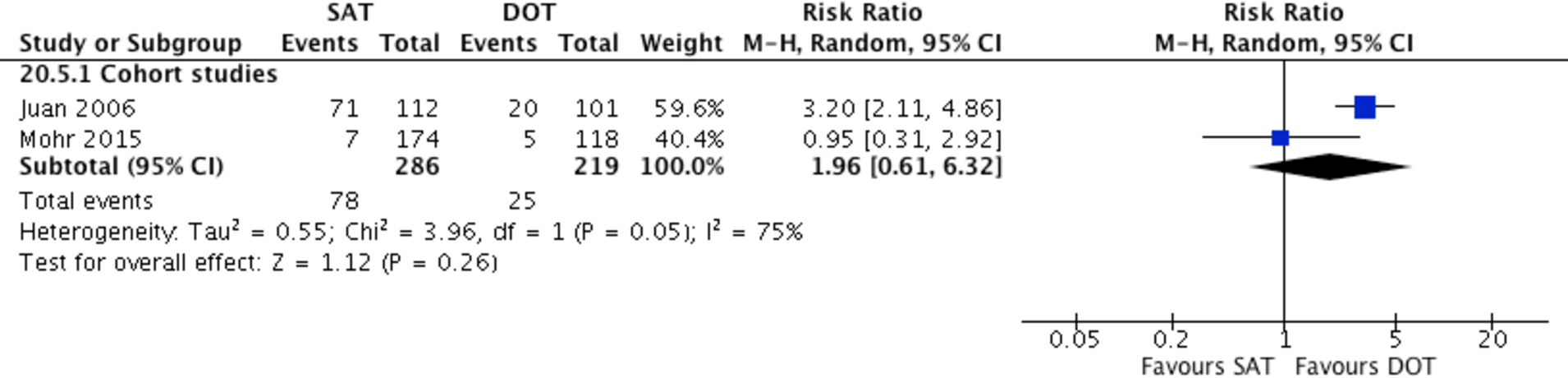

Supplement: S18 Fig — DOT, directly observed therapy; SAT, self-administered therapy; TB, tuberculosis. (TIF) [file pmed.1002595.s023.tif]

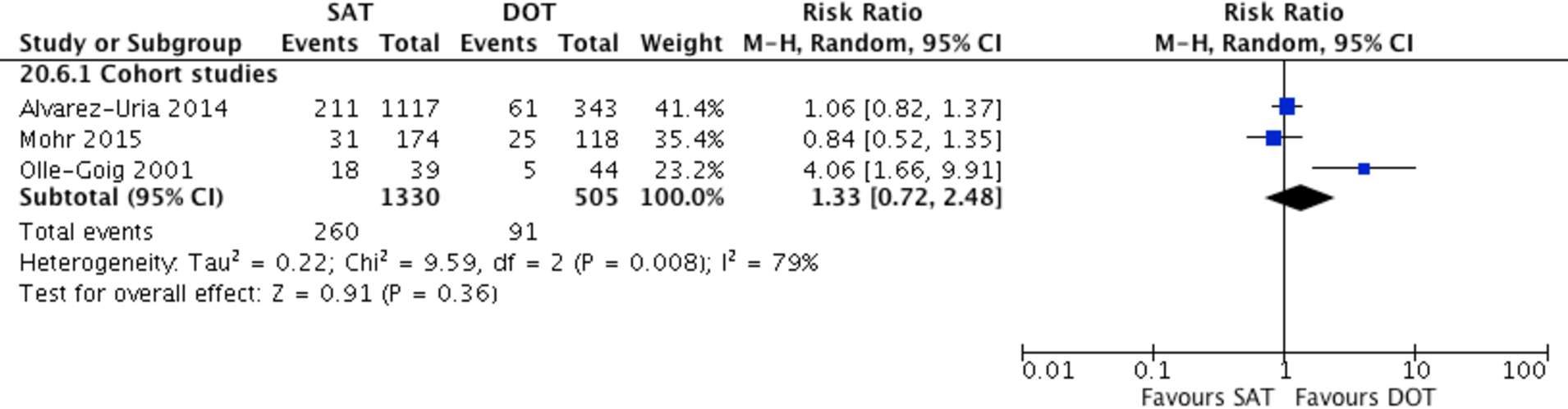

Supplement: S19 Fig — DOT, directly observed therapy; SAT, self-administered therapy; TB, tuberculosis. (TIF) [file pmed.1002595.s024.tif]

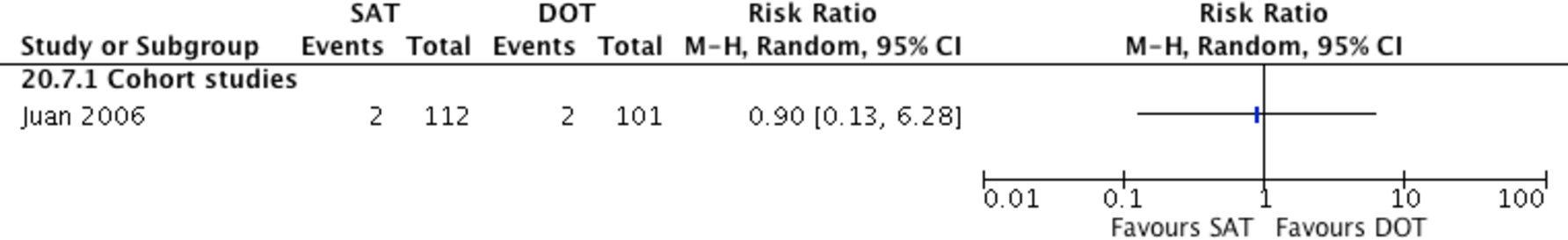

Supplement: S20 Fig — DOT, directly observed therapy; SAT, self-administered therapy; TB, tuberculosis. (TIF) [file pmed.1002595.s025.tif]

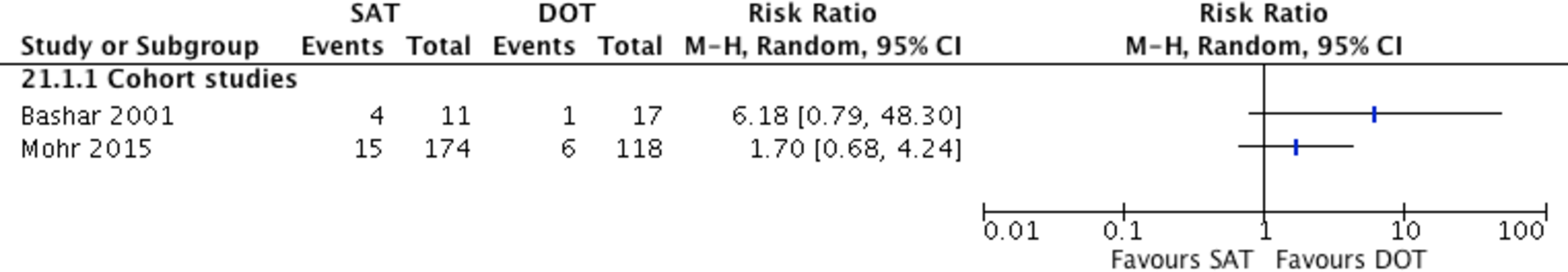

Supplement: S21 Fig — DOT, directly observed therapy; MDR-TB, multidrug-resistant tuberculosis; SAT, self-administered therapy. (TIF) [file pmed.1002595.s026.tif]

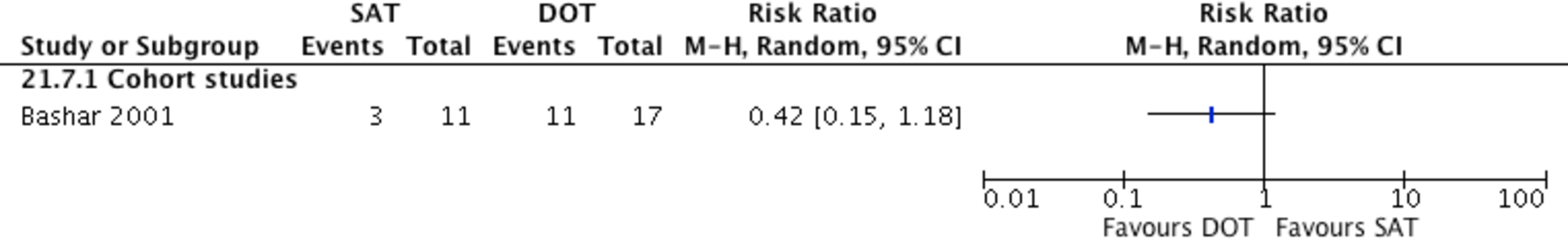

Supplement: S22 Fig — DOT, directly observed therapy; MDR-TB, multidrug-resistant tuberculosis; SAT, self-administered therapy. (TIF) [file pmed.1002595.s027.tif]

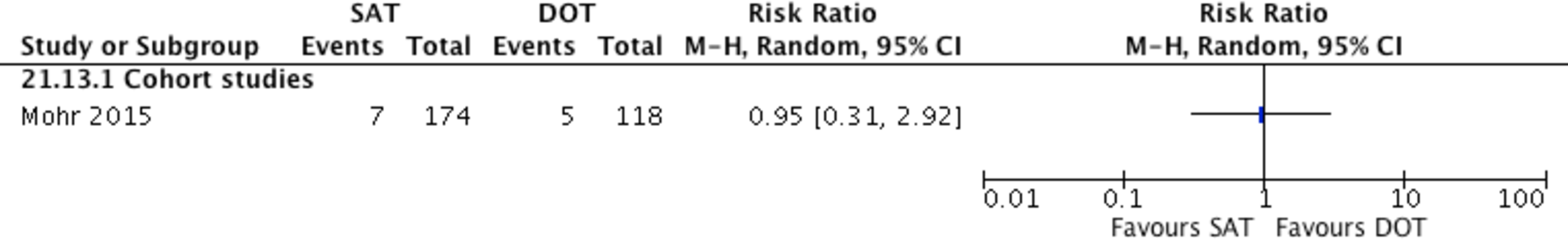

Supplement: S23 Fig — DOT, directly observed therapy; MDR-TB, multidrug-resistant tuberculosis; SAT, self-administered therapy. (TIF) [file pmed.1002595.s028.tif]

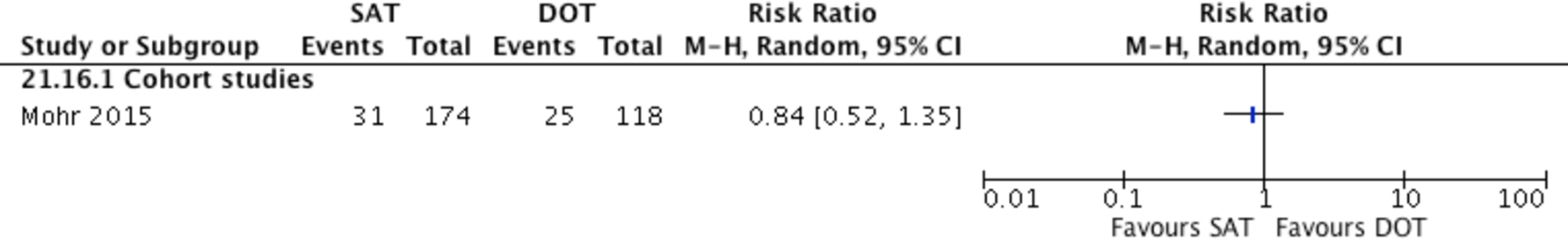

Supplement: S24 Fig — DOT, directly observed therapy; MDR-TB, multidrug-resistant tuberculosis; SAT, self-administered therapy. (TIF) [file pmed.1002595.s029.tif]

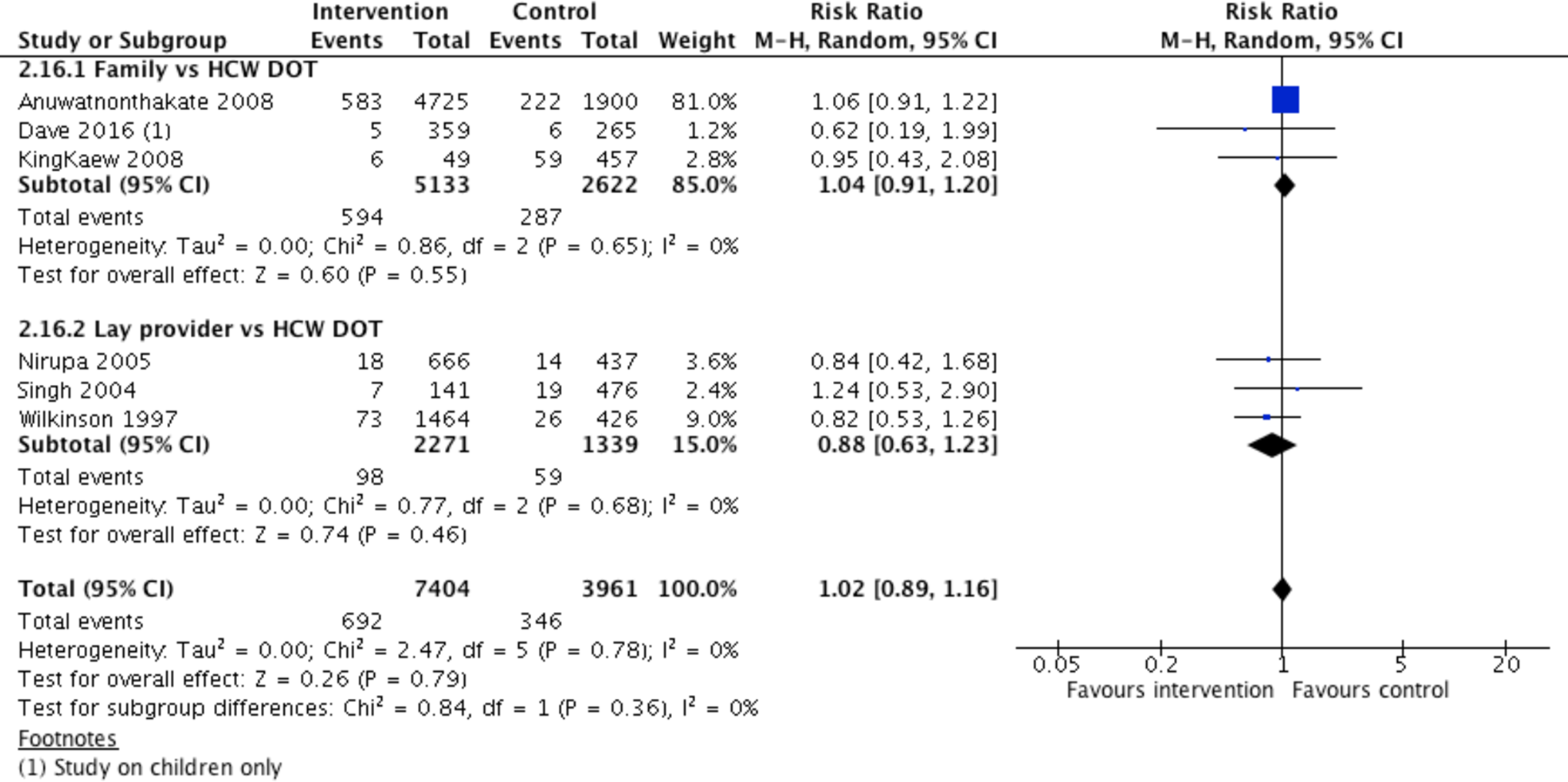

Supplement: S25 Fig — DOT, directly observed therapy. (TIF) [file pmed.1002595.s030.tif]

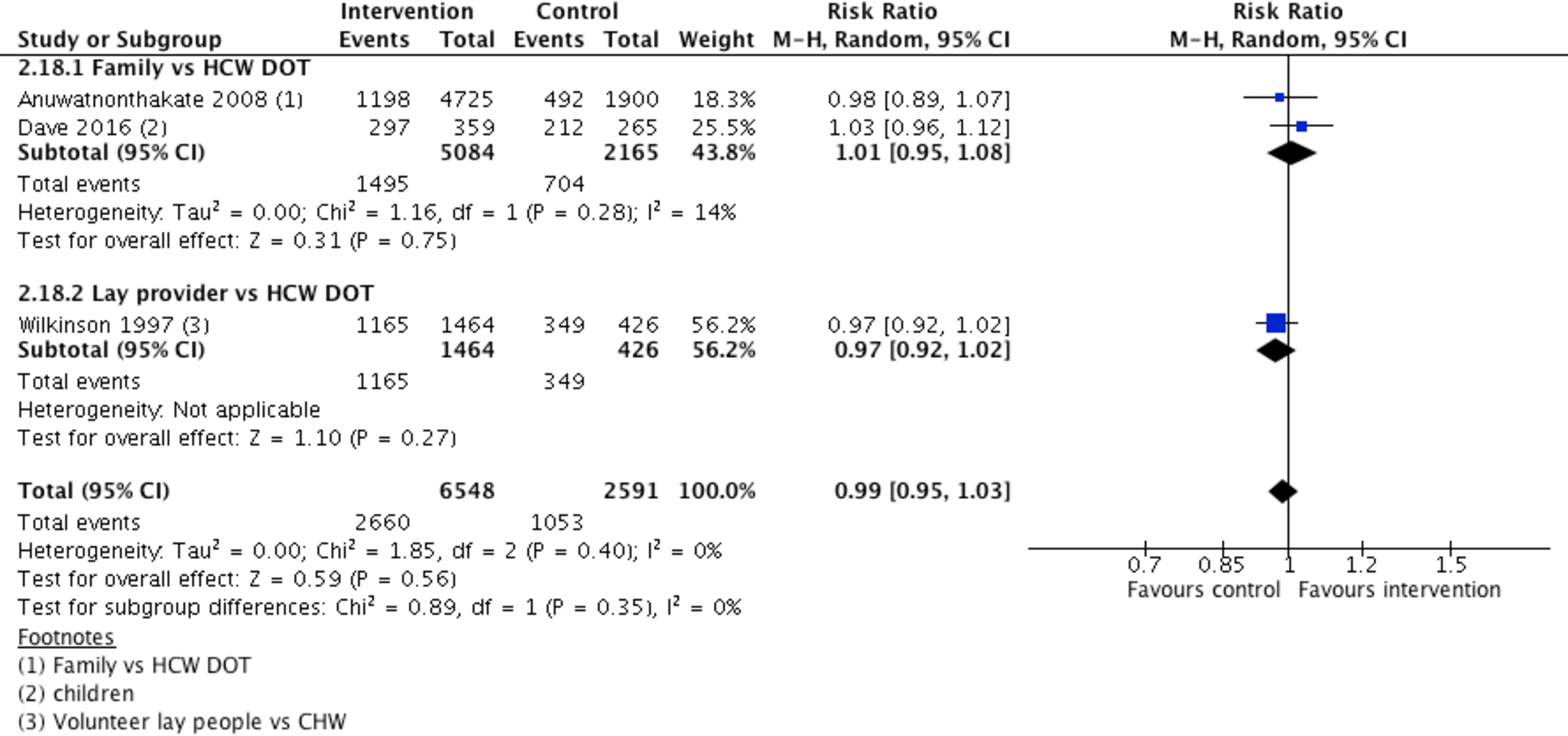

Supplement: S26 Fig — DOT, directly observed therapy. (TIF) [file pmed.1002595.s031.tif]

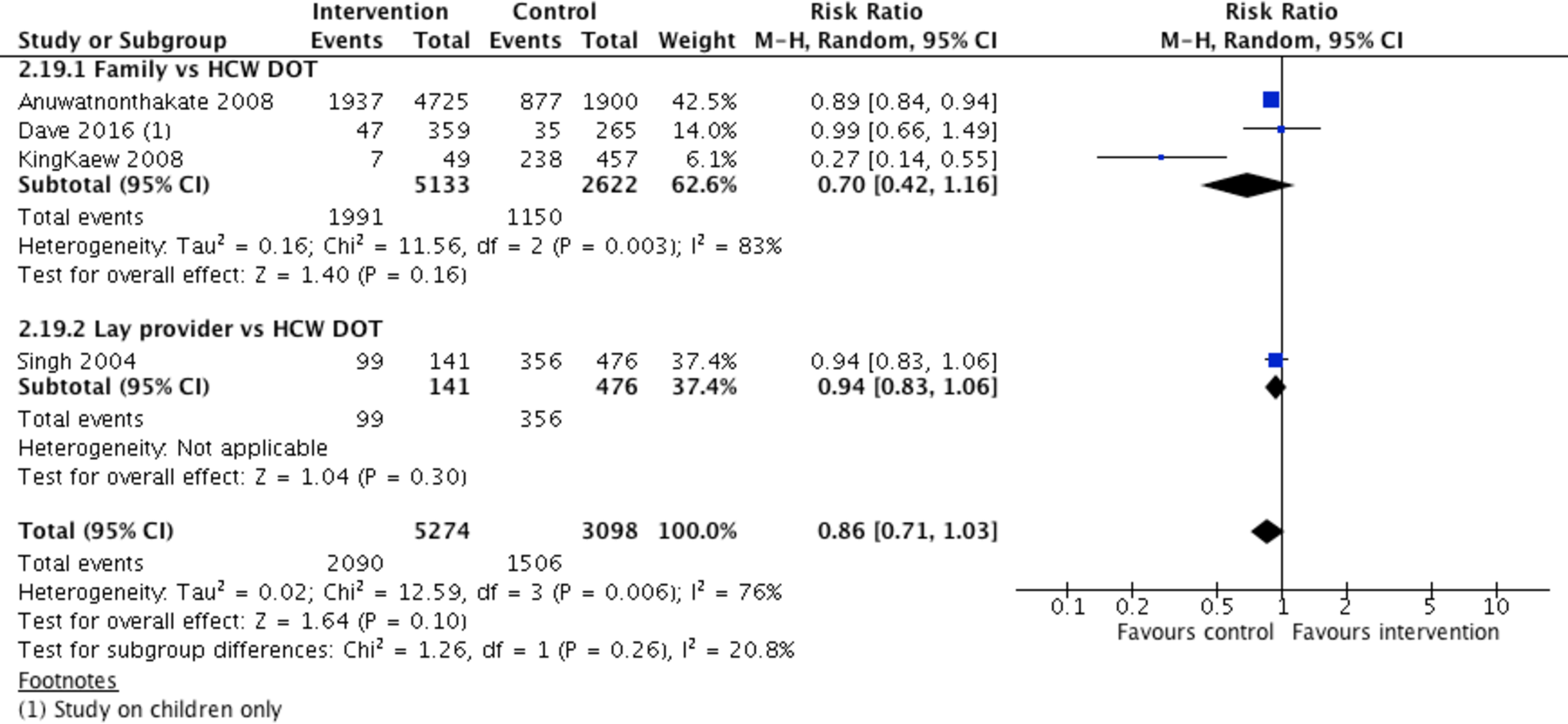

Supplement: S27 Fig — DOT, directly observed therapy. (TIF) [file pmed.1002595.s032.tif]

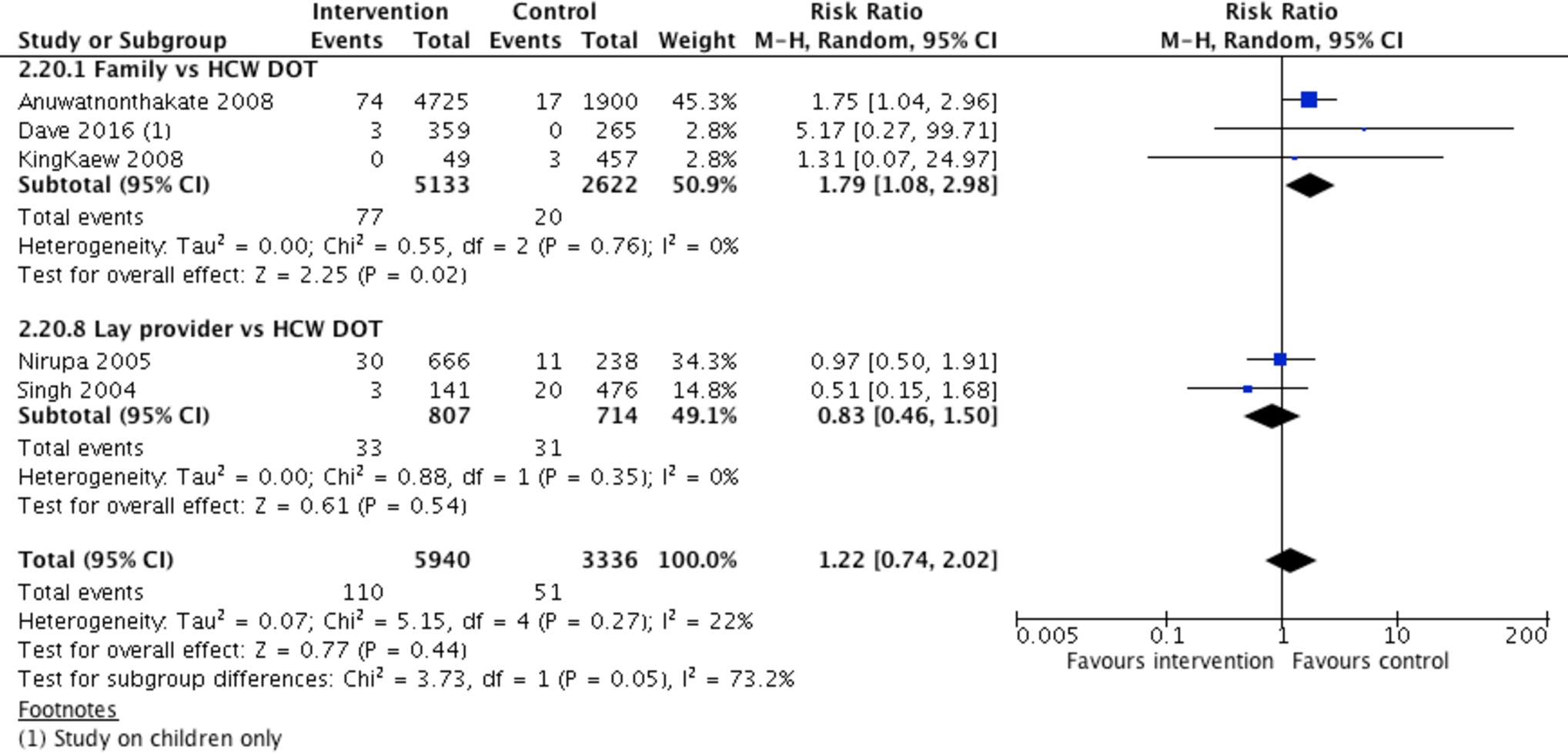

Supplement: S28 Fig — DOT, directly observed therapy. (TIF) [file pmed.1002595.s033.tif]

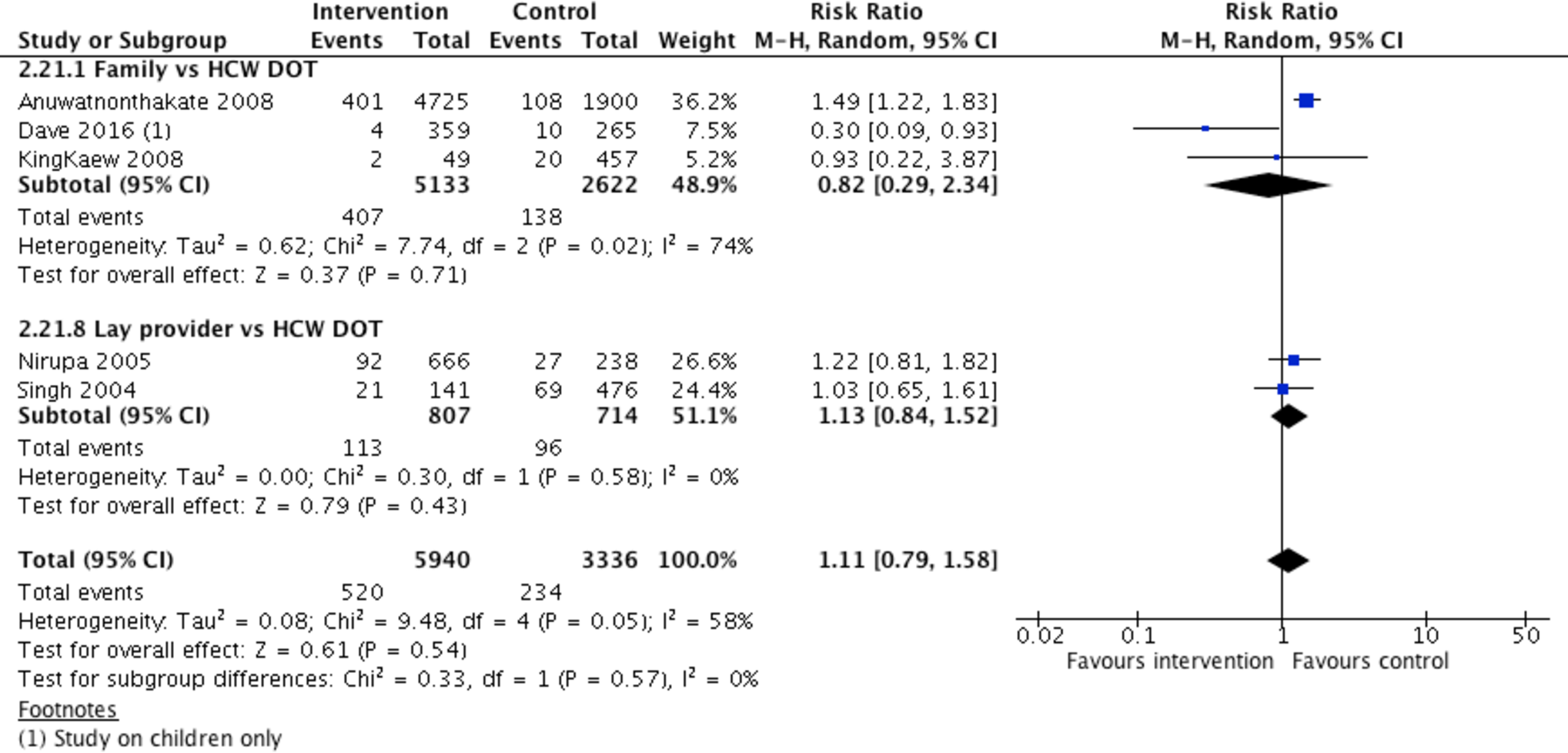

Supplement: S29 Fig — DOT, directly observed therapy. (TIF) [file pmed.1002595.s034.tif]

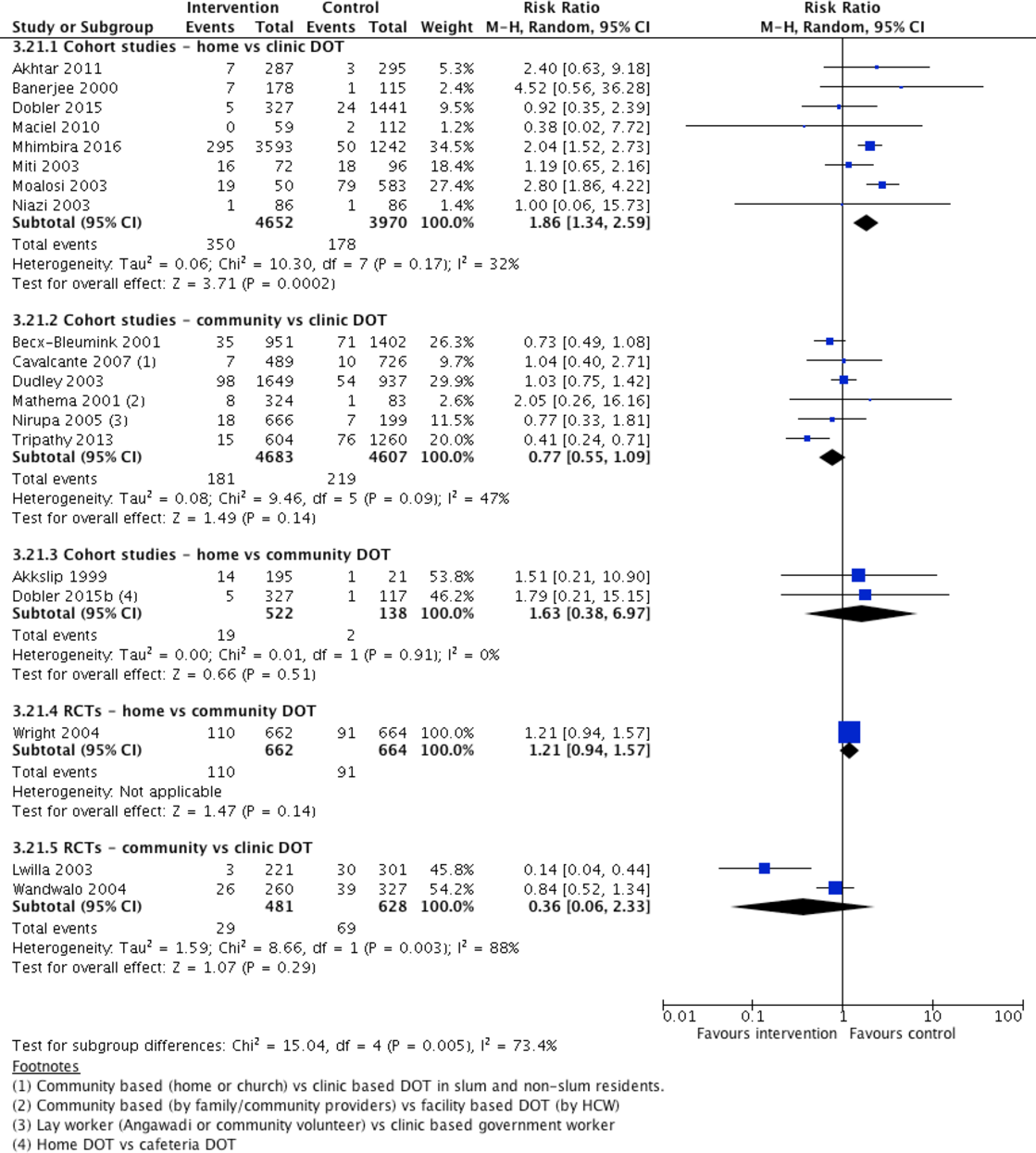

Supplement: S30 Fig — DOT, directly observed therapy. (TIF) [file pmed.1002595.s035.tif]

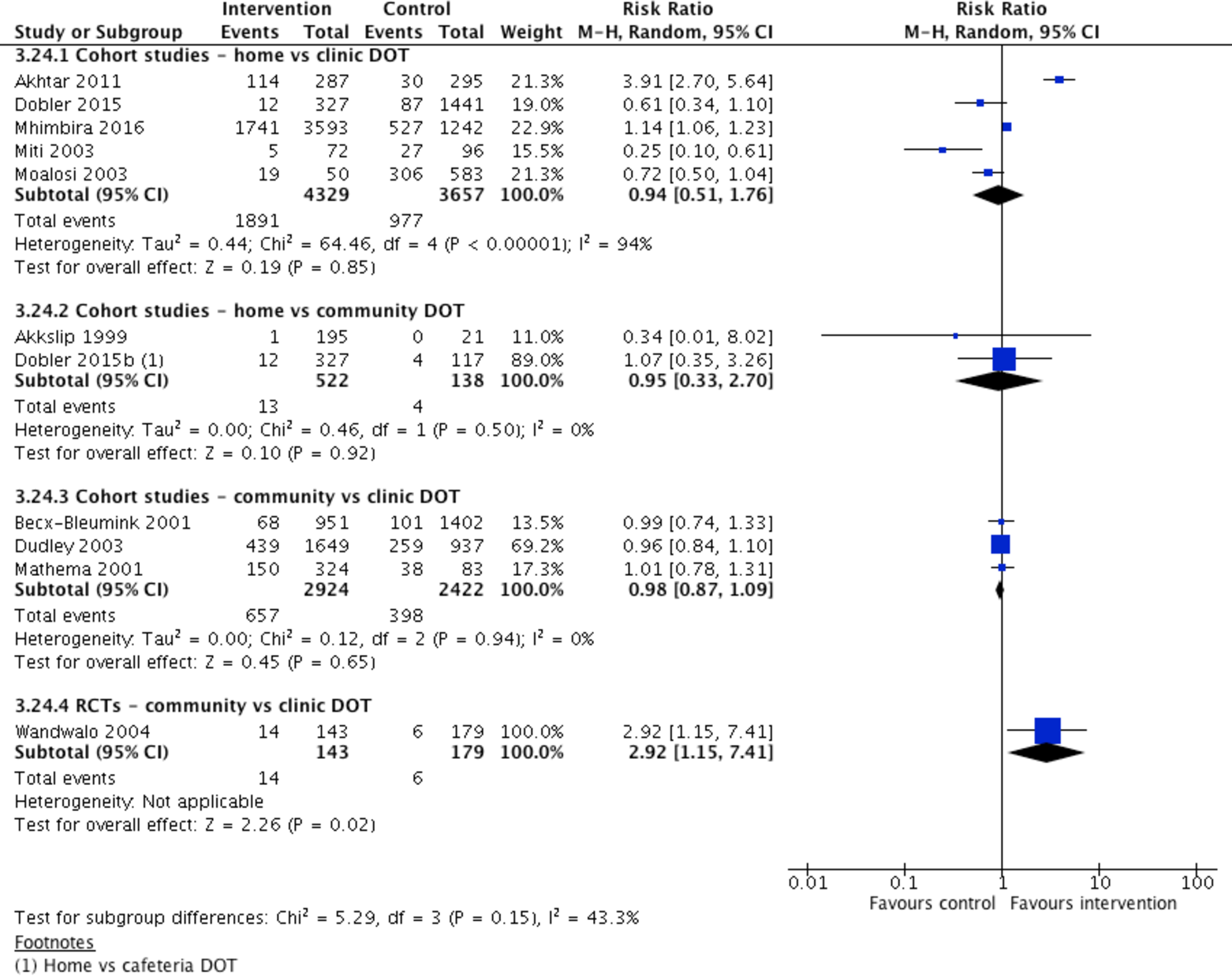

Supplement: S31 Fig — DOT, directly observed therapy. (TIF) [file pmed.1002595.s036.tif]

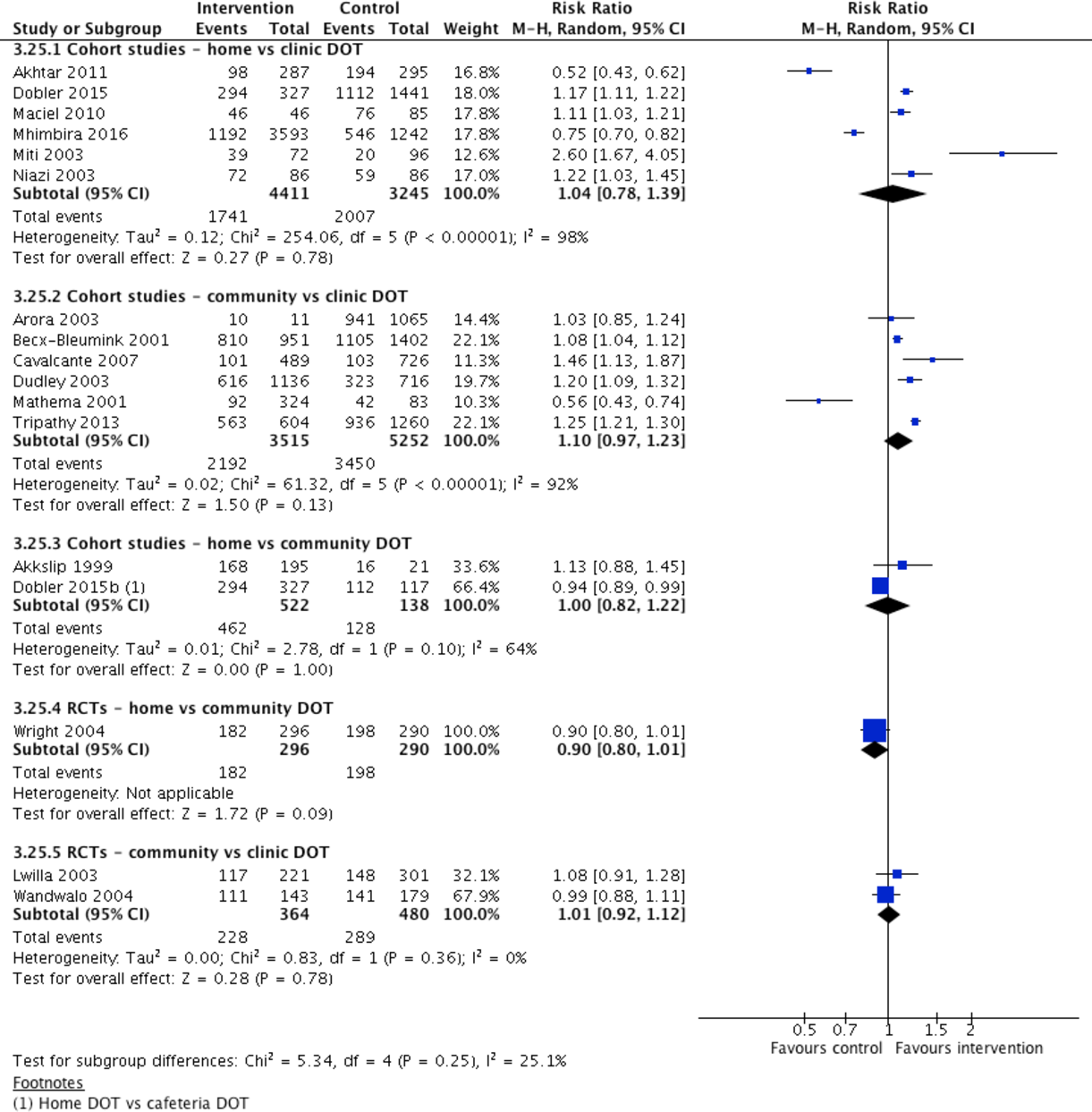

Supplement: S32 Fig — DOT, directly observed therapy. (TIF) [file pmed.1002595.s037.tif]

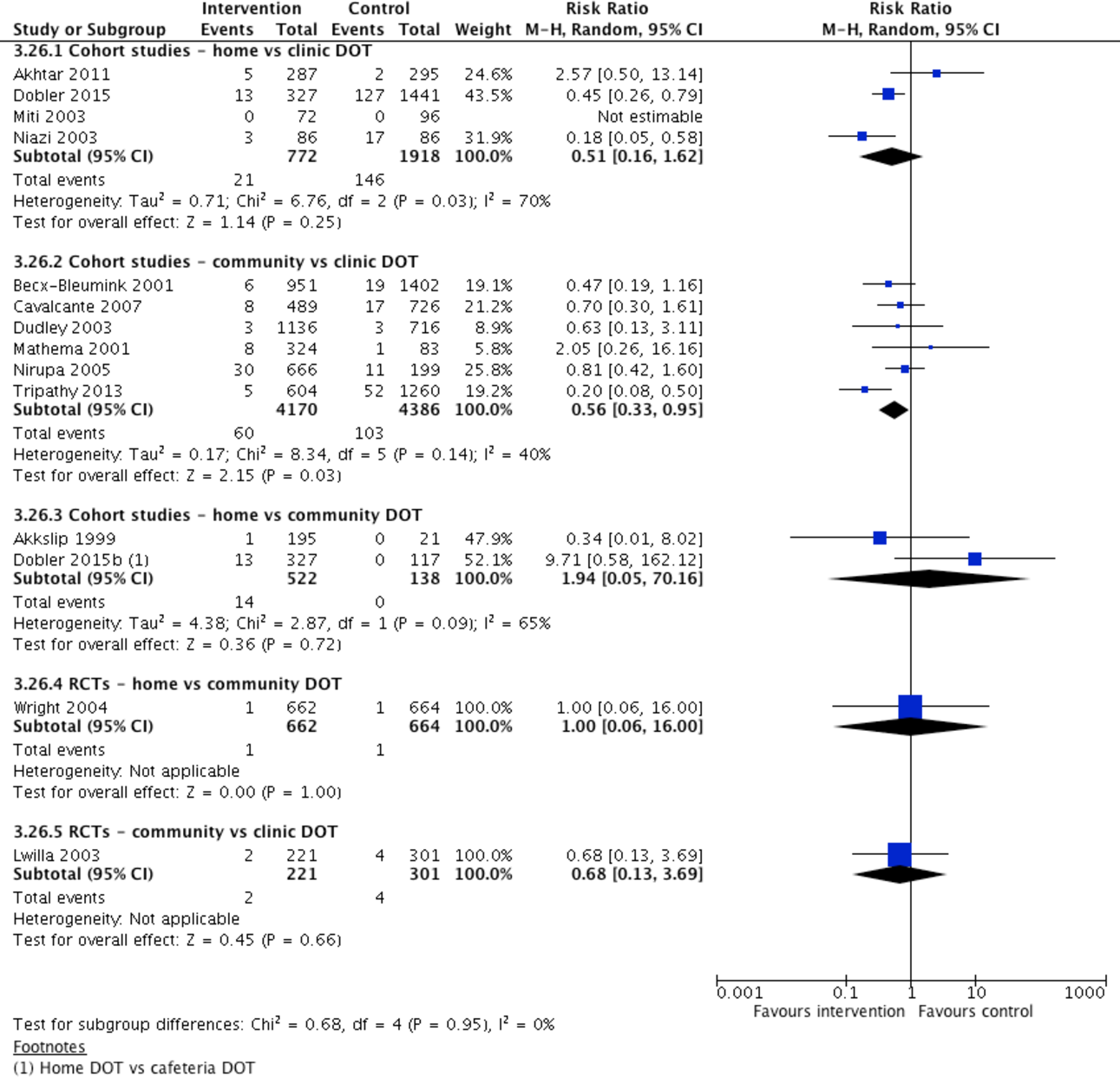

Supplement: S33 Fig — DOT, directly observed therapy. (TIF) [file pmed.1002595.s038.tif]

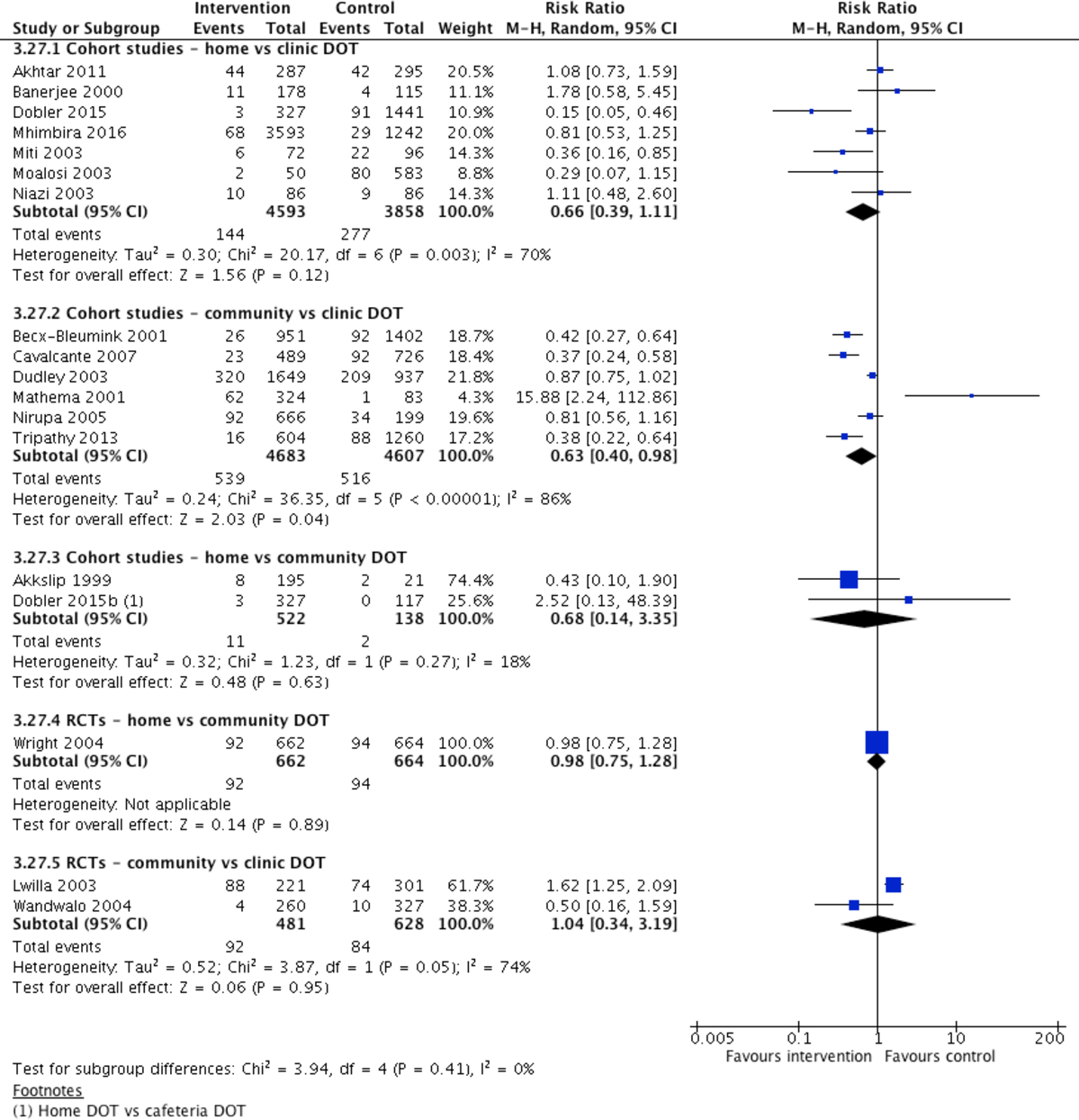

Supplement: S34 Fig — DOT, directly observed therapy. (TIF) [file pmed.1002595.s039.tif]

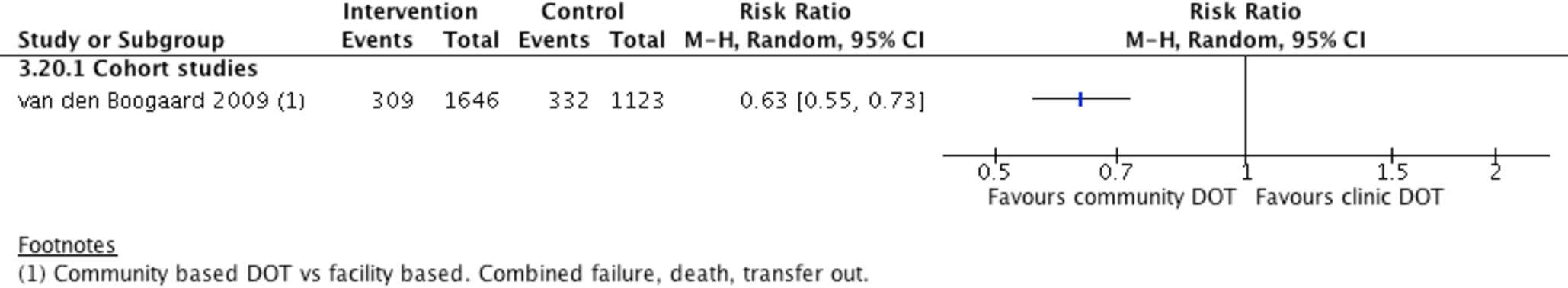

Supplement: S35 Fig — DOT, directly observed therapy. (TIF) [file pmed.1002595.s040.tif]

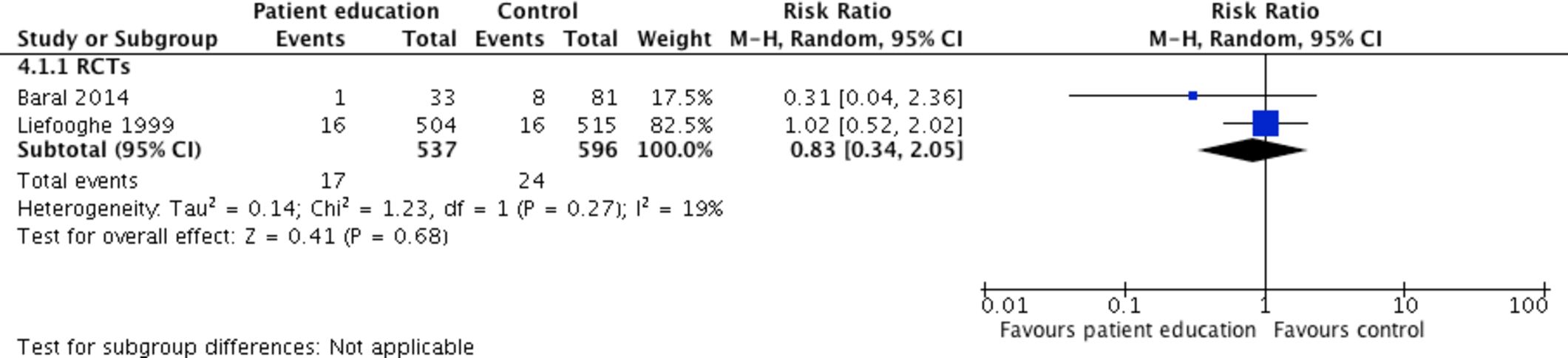

Supplement: S36 Fig — (TIF) [file pmed.1002595.s041.tif]

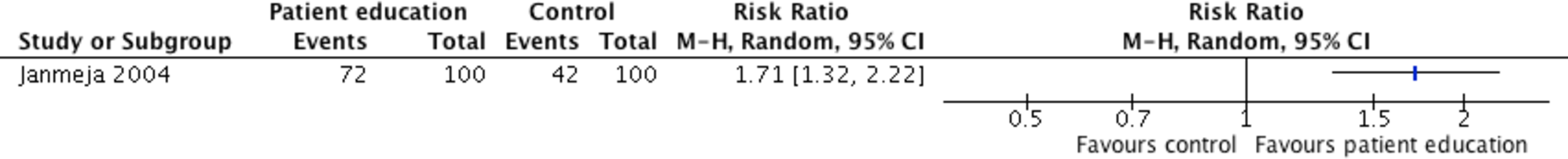

Supplement: S37 Fig — (TIF) [file pmed.1002595.s042.tif]

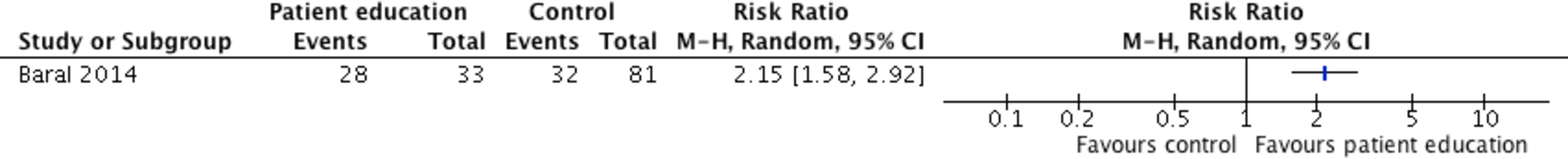

Supplement: S38 Fig — (TIF) [file pmed.1002595.s043.tif]

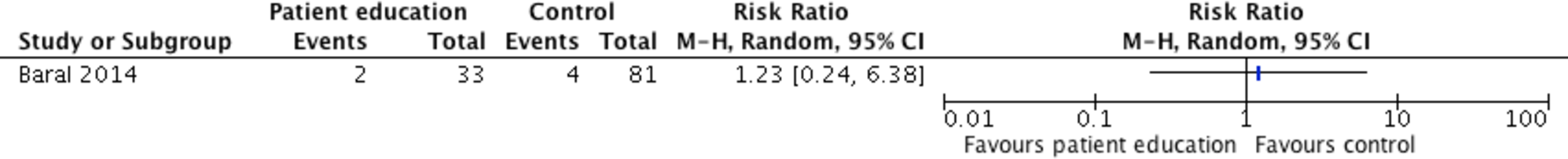

Supplement: S39 Fig — (TIF) [file pmed.1002595.s044.tif]

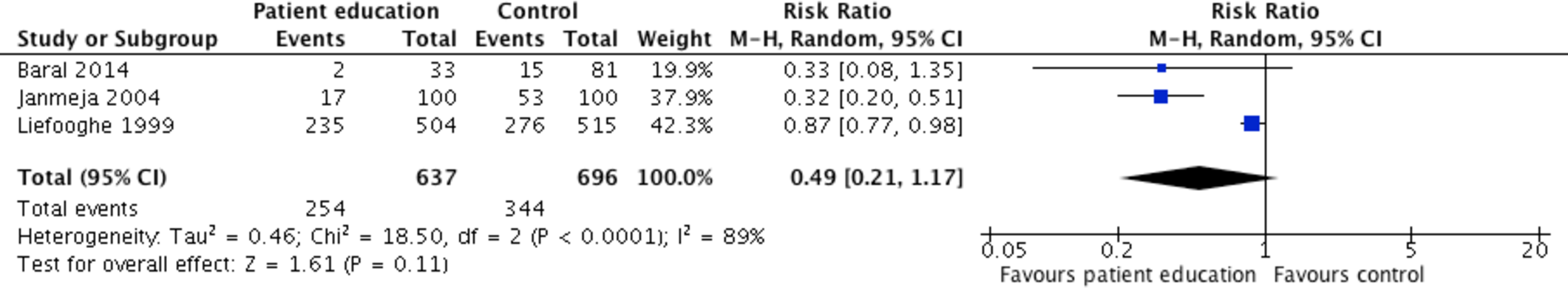

Supplement: S40 Fig — (TIF) [file pmed.1002595.s045.tif]

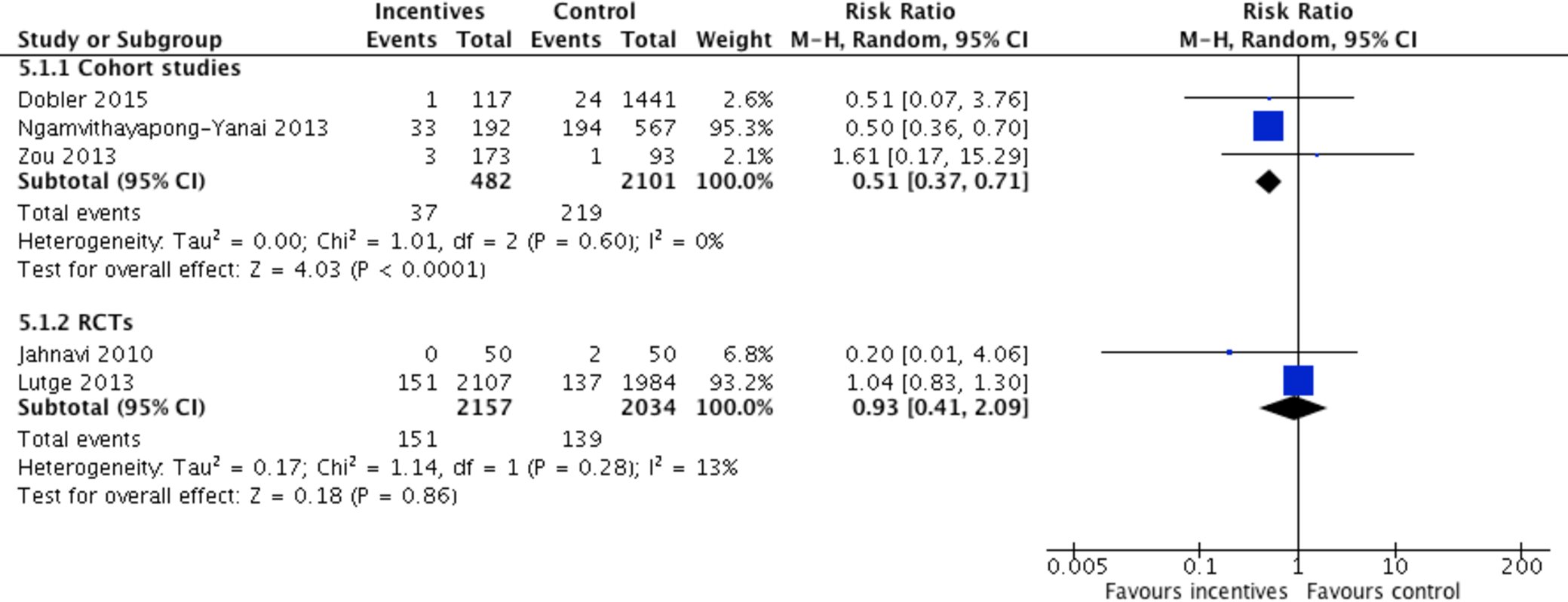

Supplement: S41 Fig — (TIF) [file pmed.1002595.s046.tif]

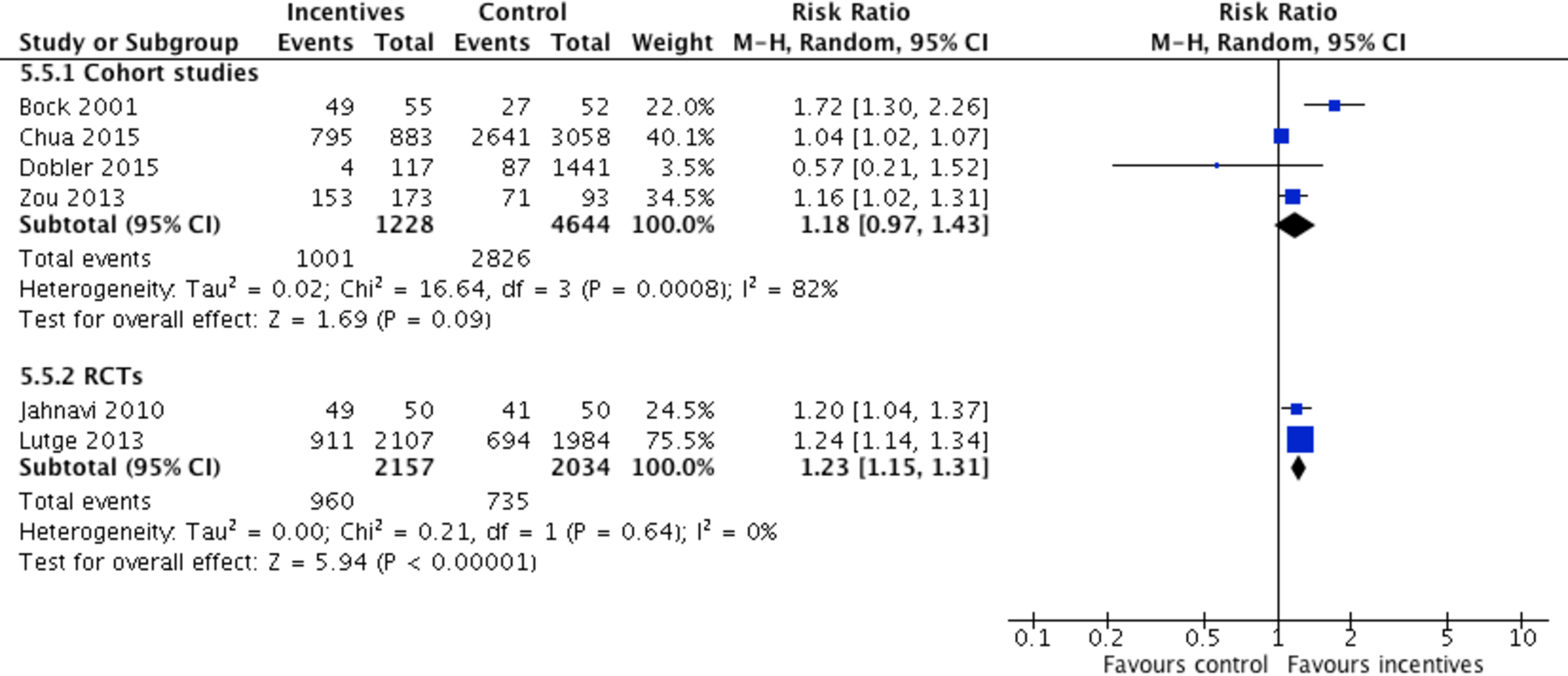

Supplement: S42 Fig — (TIF) [file pmed.1002595.s047.tif]

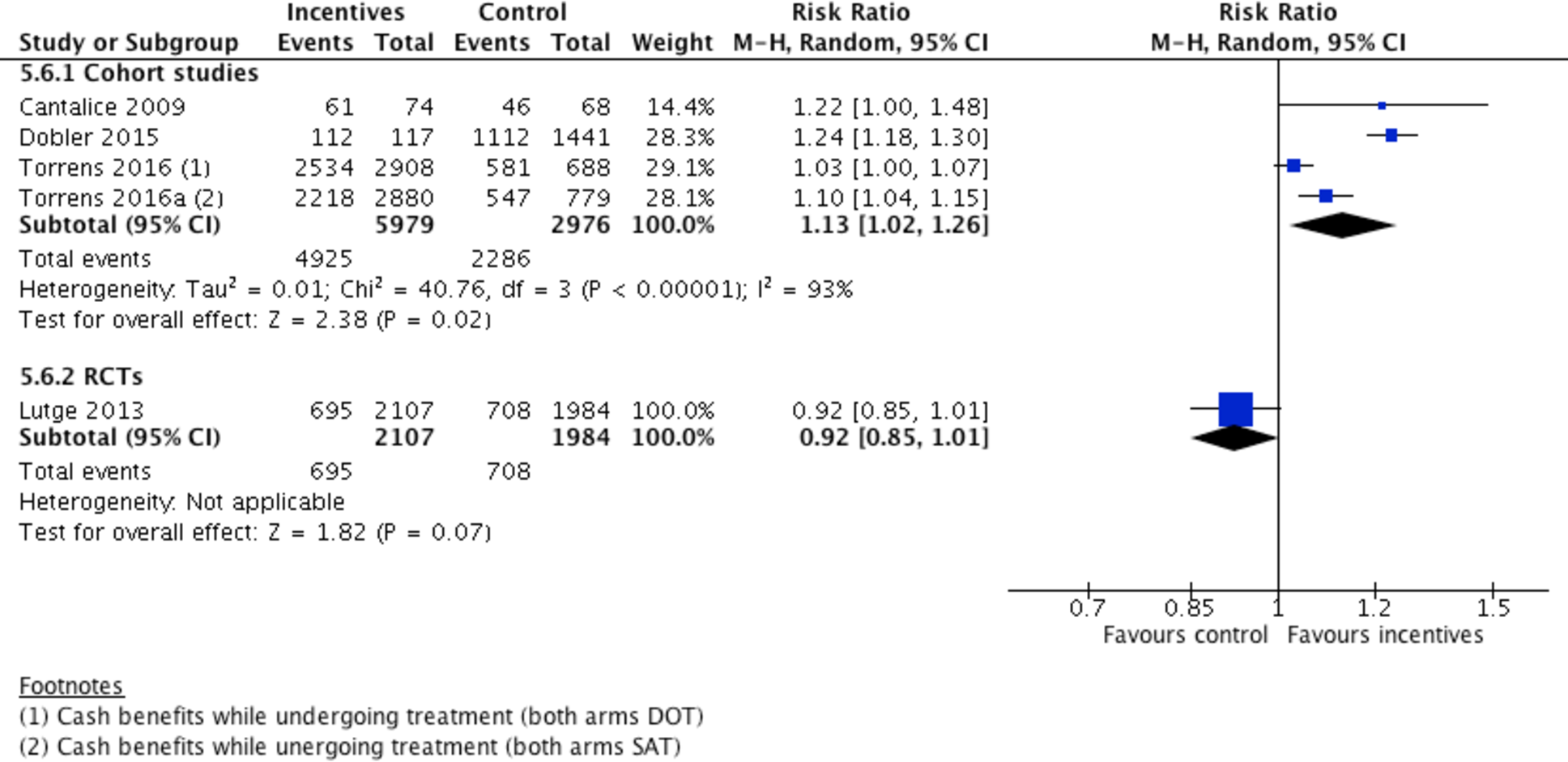

Supplement: S43 Fig — (TIF) [file pmed.1002595.s048.tif]

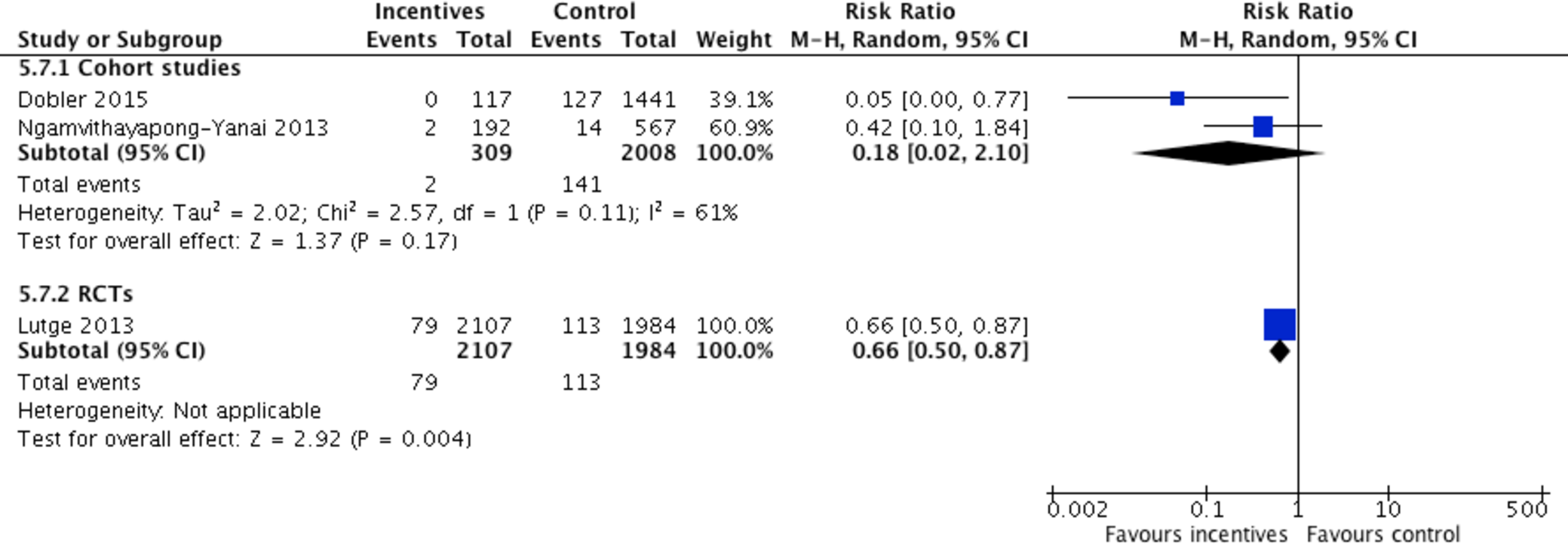

Supplement: S44 Fig — (TIF) [file pmed.1002595.s049.tif]

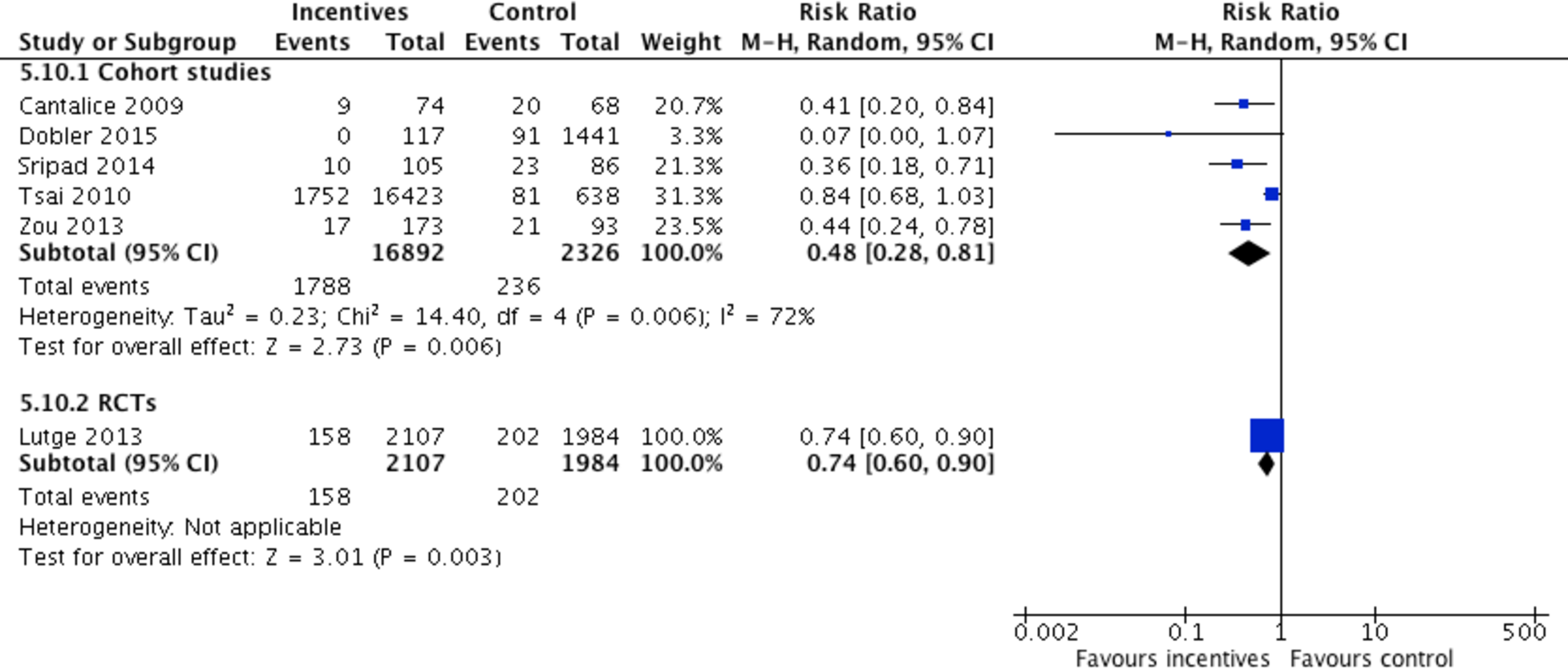

Supplement: S45 Fig — (TIF) [file pmed.1002595.s050.tif]

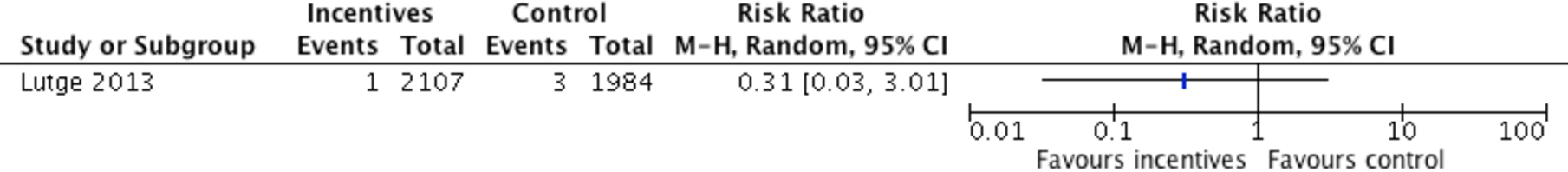

Supplement: S46 Fig — (TIF) [file pmed.1002595.s051.tif]

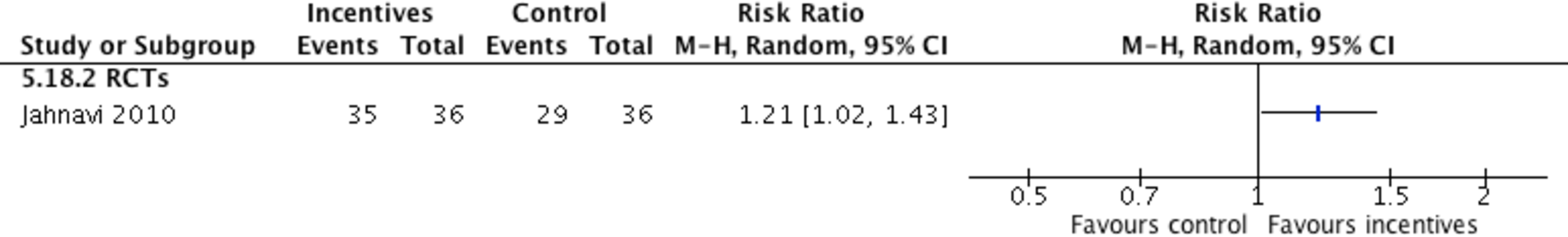

Supplement: S47 Fig — (TIF) [file pmed.1002595.s052.tif]

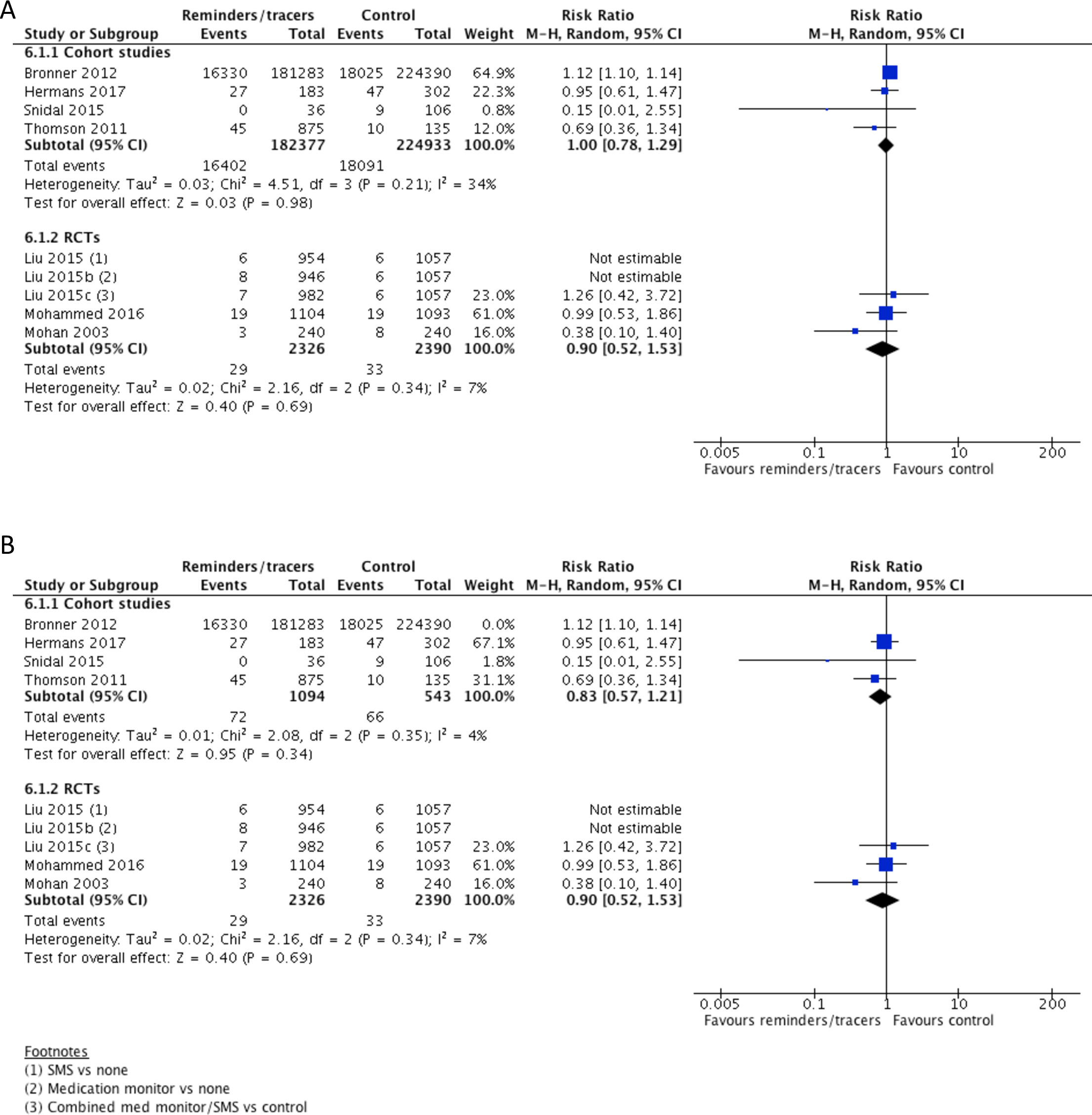

Supplement: S48 Fig — (A) Meta-analysis of mortality rates in patients receiving reminders/tracers in addition to standard care versus standard care alone. (B) Sensitivity analysis: removing the heaviest weighted study (Bronner 2012) in which control and intervention cohorts had significantly different pre-intervention mortality rates. “Not estimable” denotes a subgroup within a study not included in the meta-analysis. (TIF) [file pmed.1002595.s053.tif]

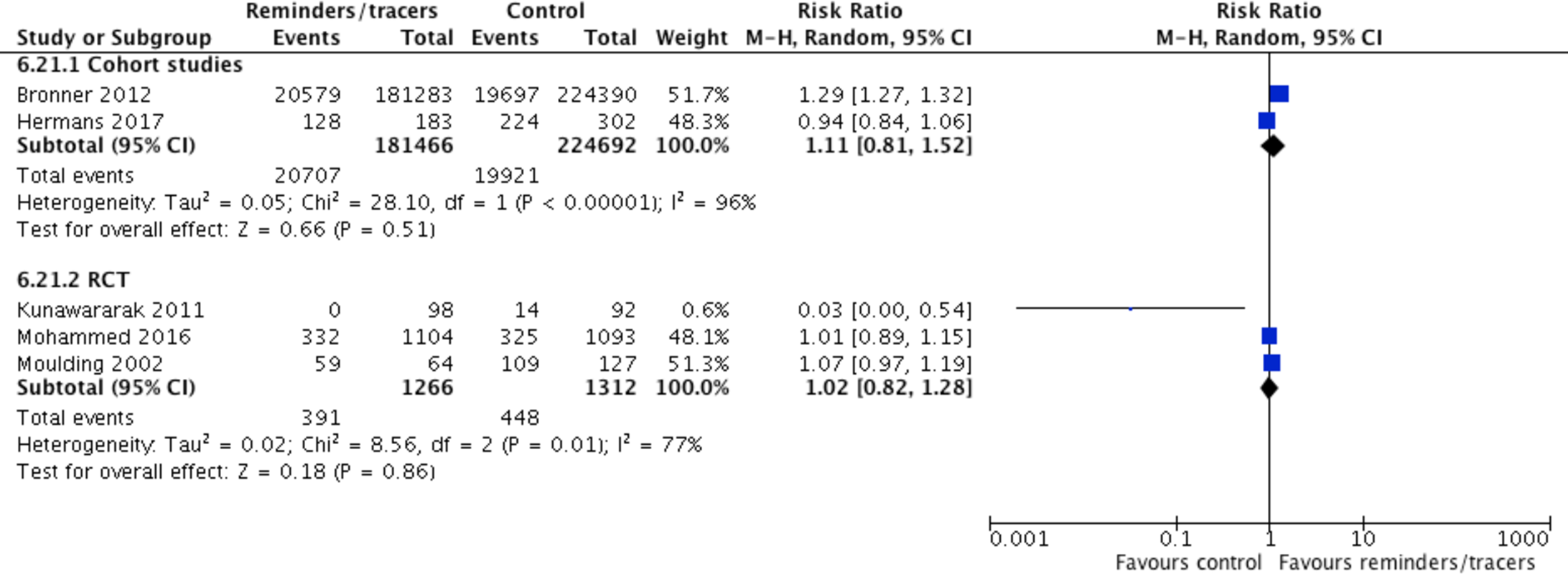

Supplement: S49 Fig — (TIF) [file pmed.1002595.s054.tif]

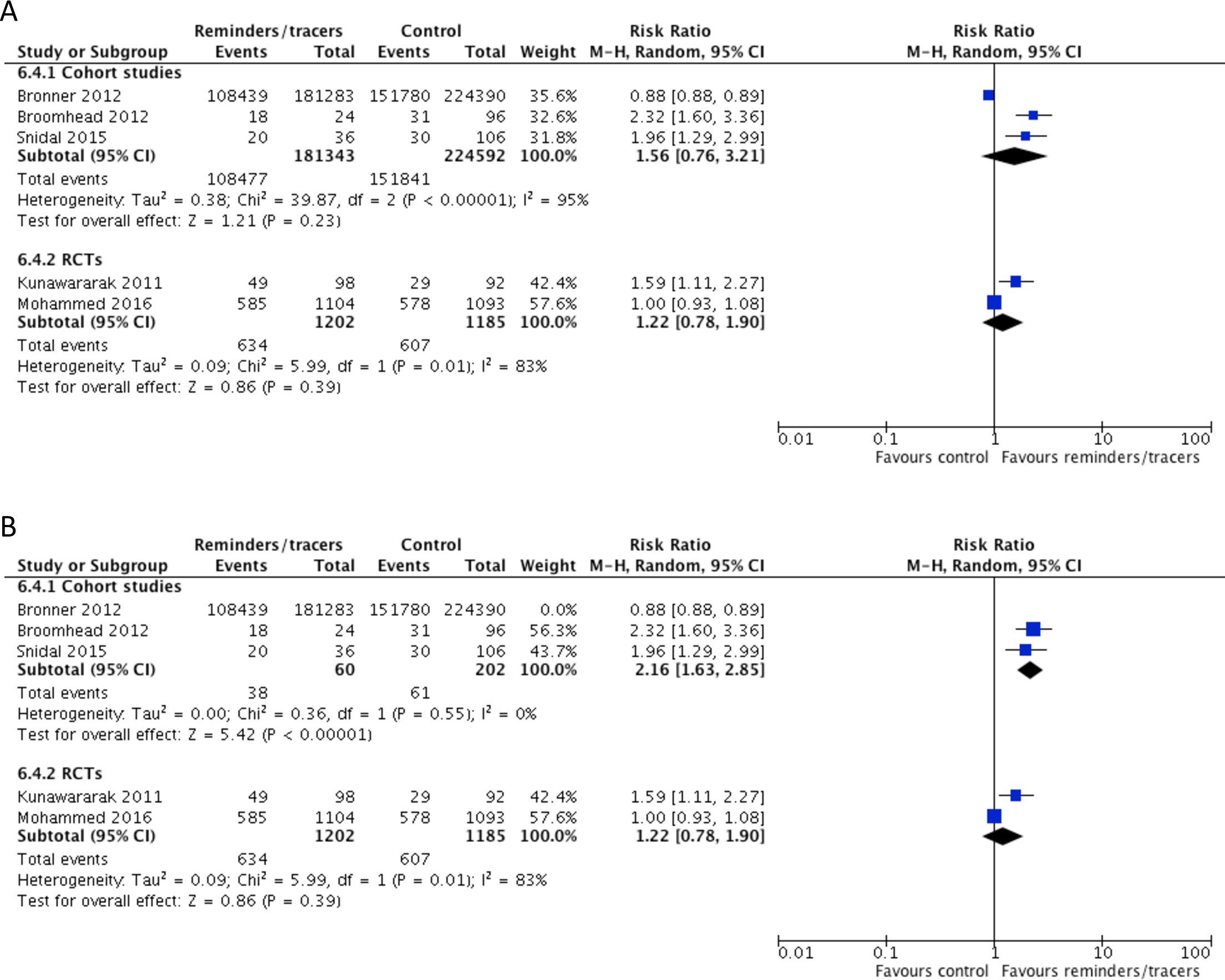

Supplement: S50 Fig — (A) Meta-analysis of cure rates in patients receiving reminders/tracers in addition to standard care versus standard care alone. (B) Sensitivity analysis: removing the heaviest weighted study (Bronner 2012) in which control and intervention cohorts had significantly different pre-intervention cure rates. (TIF) [file pmed.1002595.s055.tif]

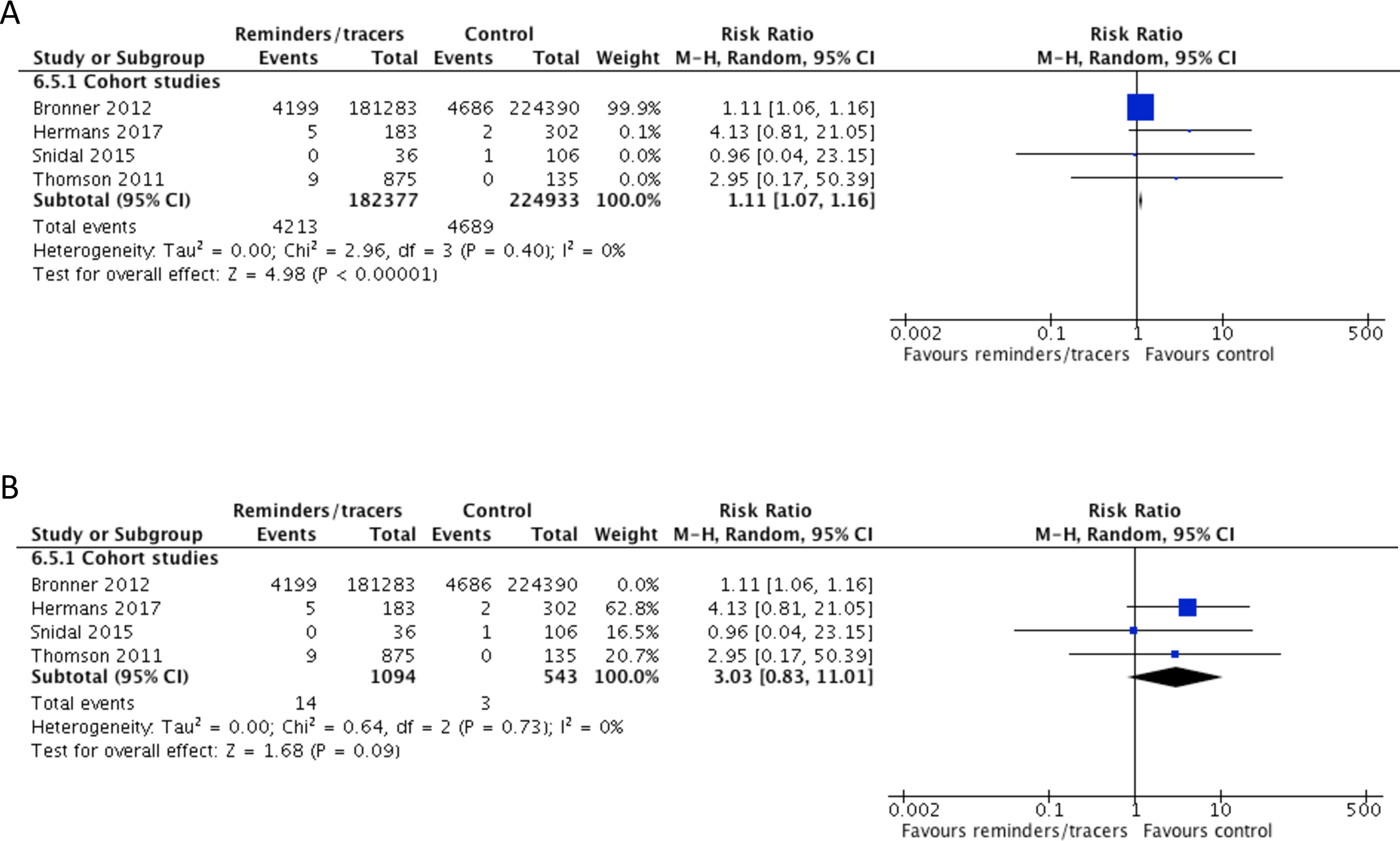

Supplement: S51 Fig — (A) Meta-analysis of treatment failure rates in patients receiving reminders/tracers in addition to standard care versus standard care alone—cohort studies. (B) Sensitivity analysis: removing the heaviest weighted study (Bronner 2012) in which control and intervention cohorts had significantly different pre-intervention treatment failure rates. (TIF) [file pmed.1002595.s056.tif]

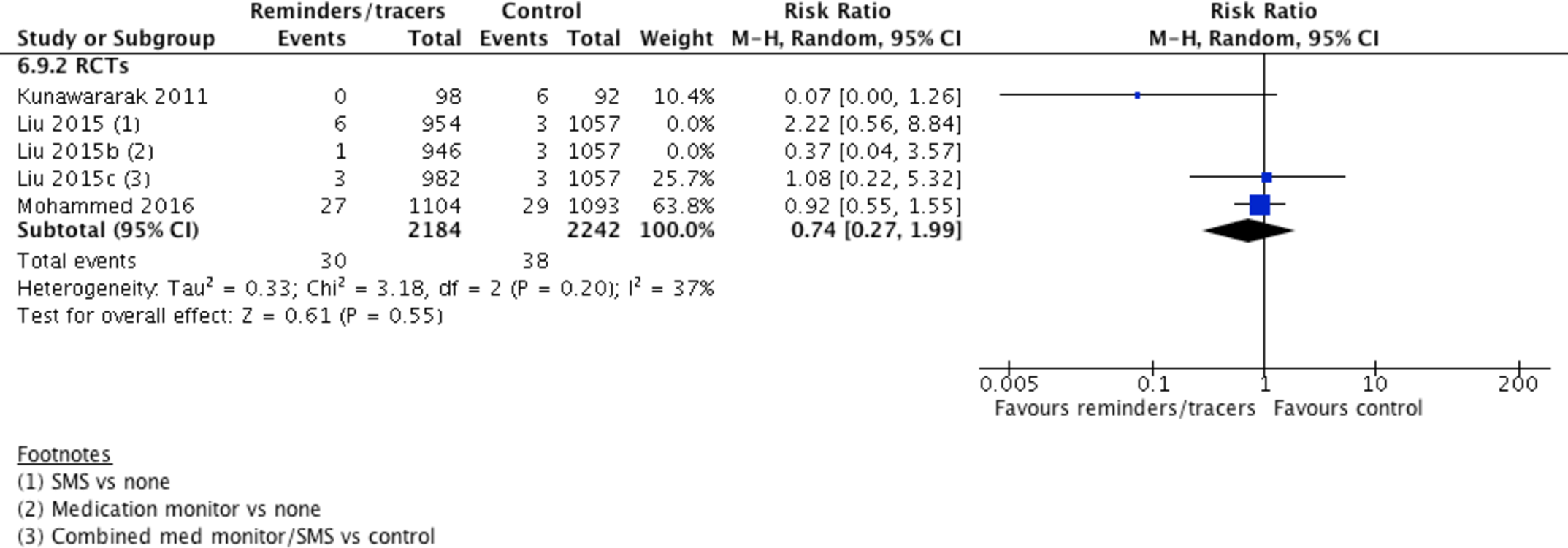

Supplement: S52 Fig — RCT, randomized controlled trial. (TIF) [file pmed.1002595.s057.tif]

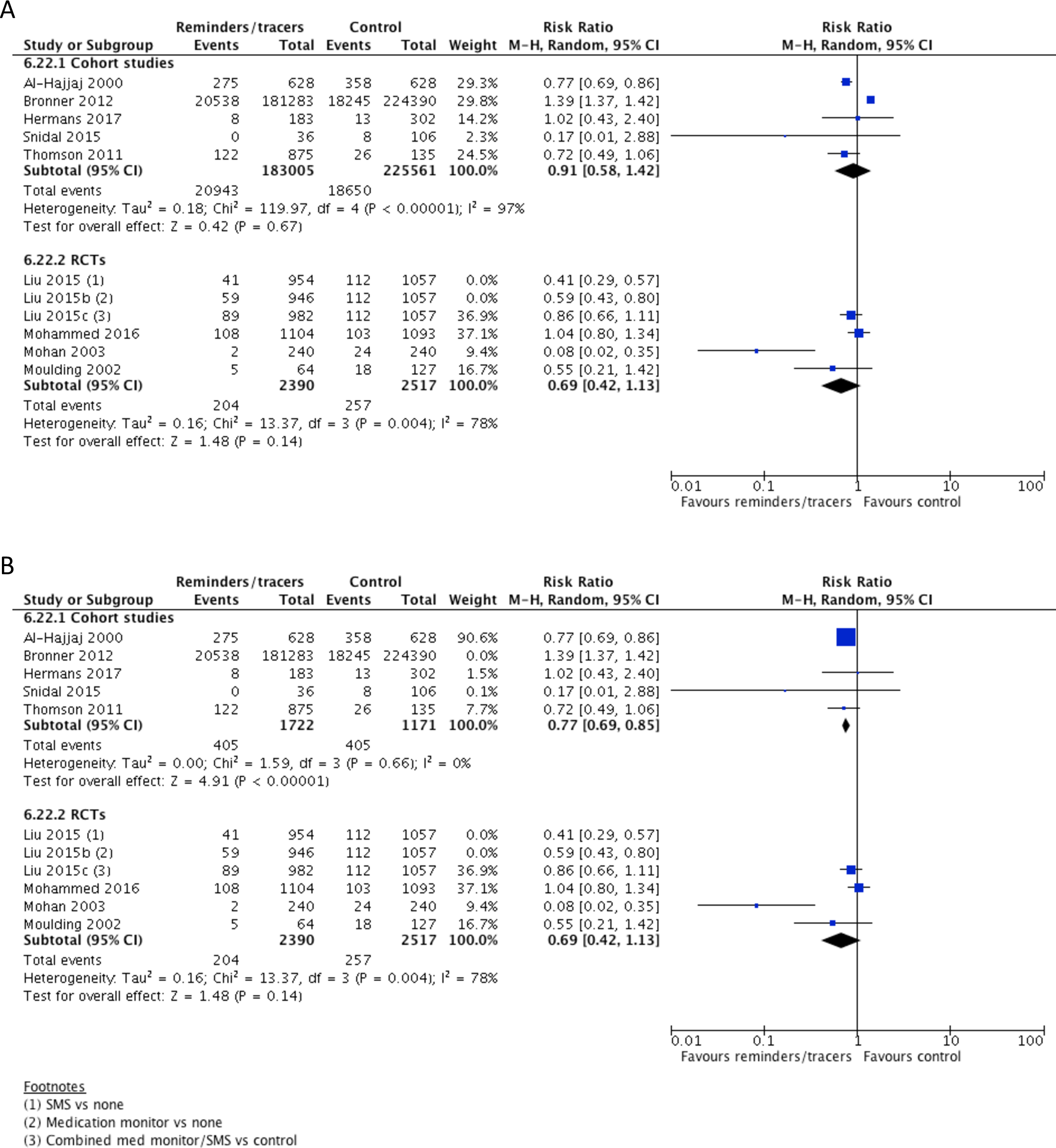

Supplement: S53 Fig — (A) Meta-analysis of rates of loss to follow-up in patients receiving reminders/tracers in addition to standard care versus standard care alone. (B) Sensitivity analysis: removing the heaviest weighted study (Bronner 2012) in which control and intervention cohorts had significantly different pre-intervention loss to follow-up rates. (TIF) [file pmed.1002595.s058.tif]

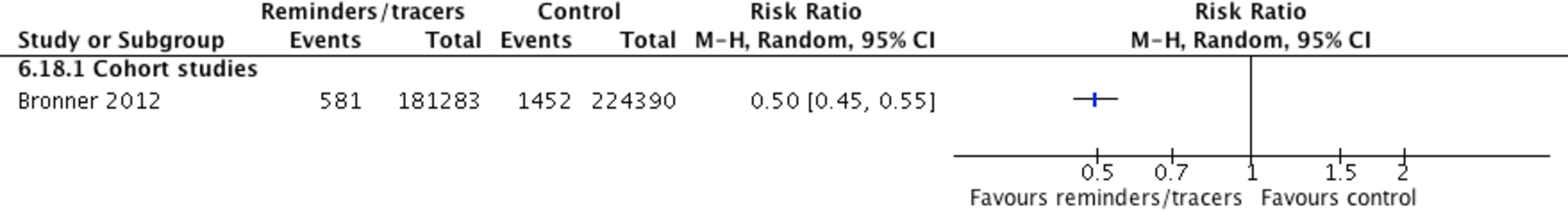

Supplement: S54 Fig — (TIF) [file pmed.1002595.s059.tif]

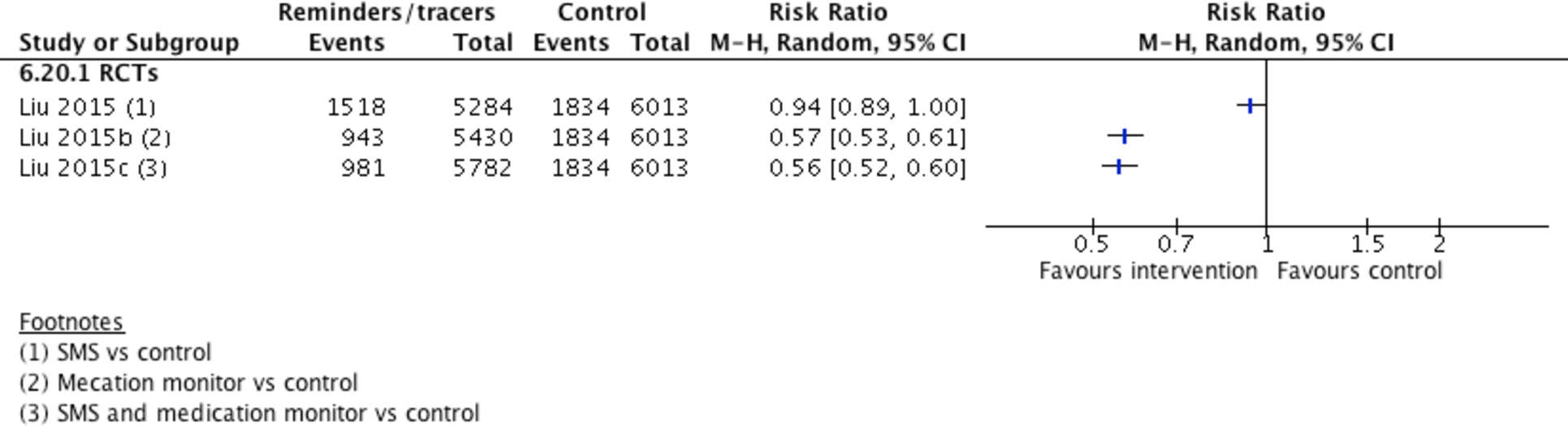

Supplement: S55 Fig — Poor adherence is defined as the percentage of patient-months in which at least 20% of doses were missed. (TIF) [file pmed.1002595.s060.tif]

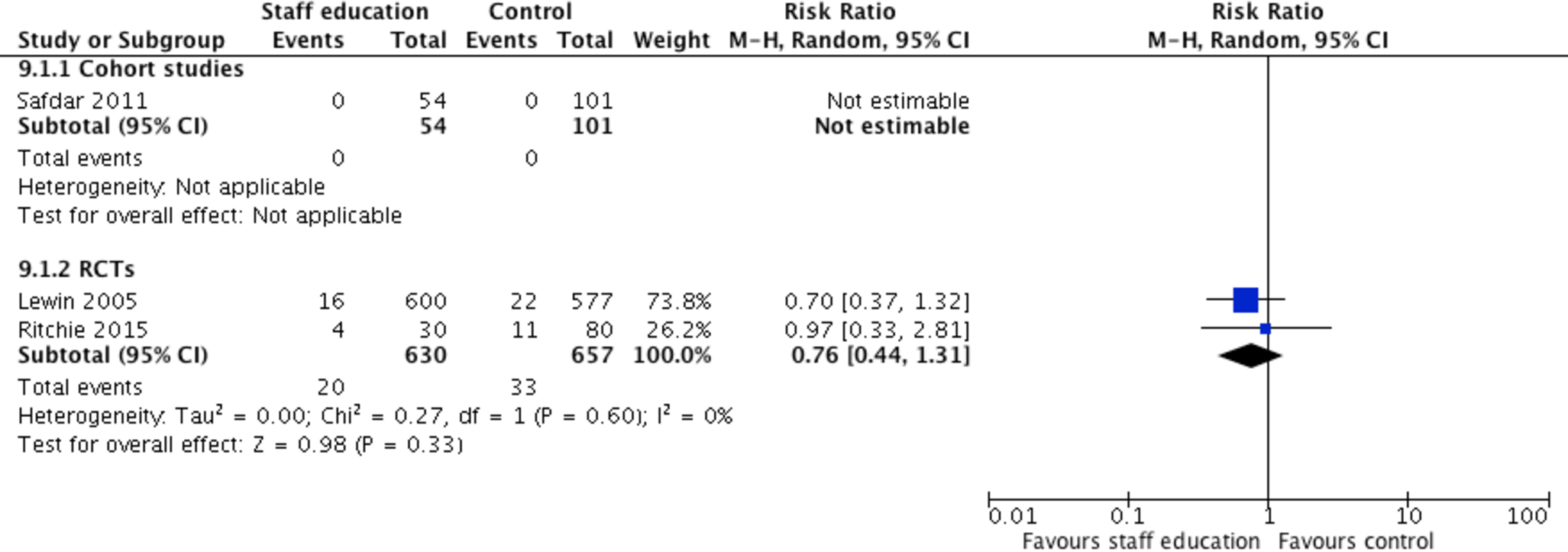

Supplement: S56 Fig — (TIF) [file pmed.1002595.s061.tif]

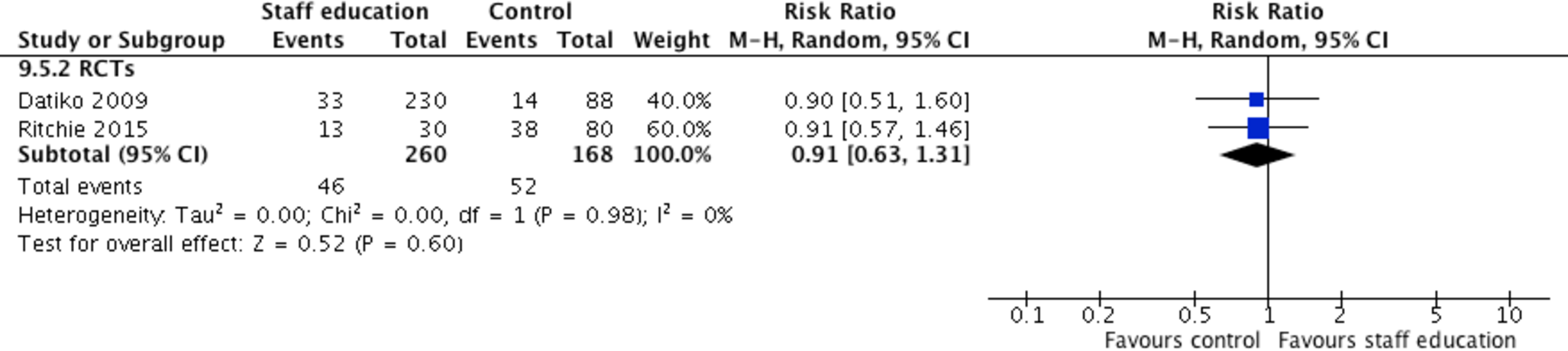

Supplement: S57 Fig — (TIF) [file pmed.1002595.s062.tif]

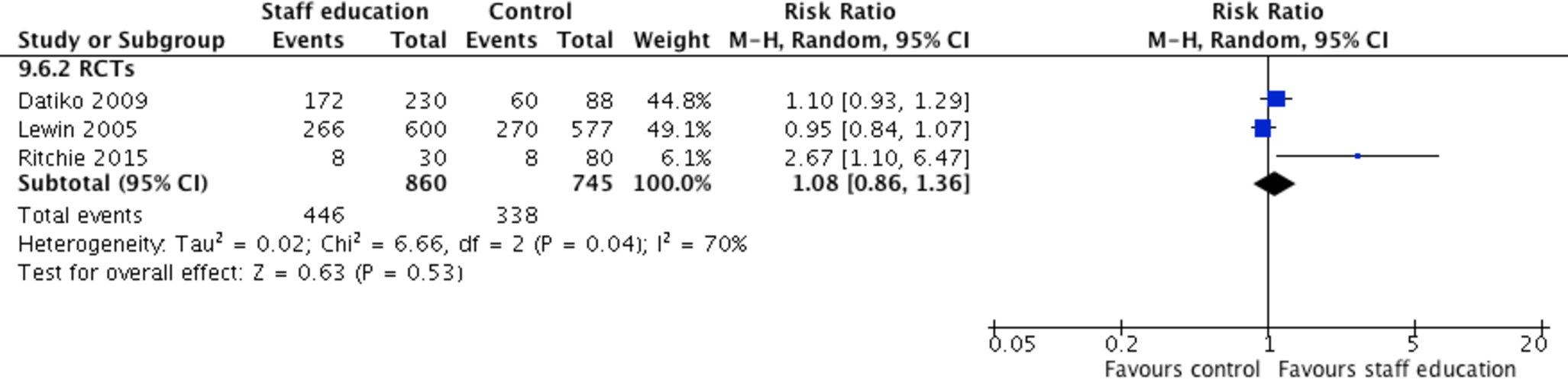

Supplement: S58 Fig — (TIF) [file pmed.1002595.s063.tif]

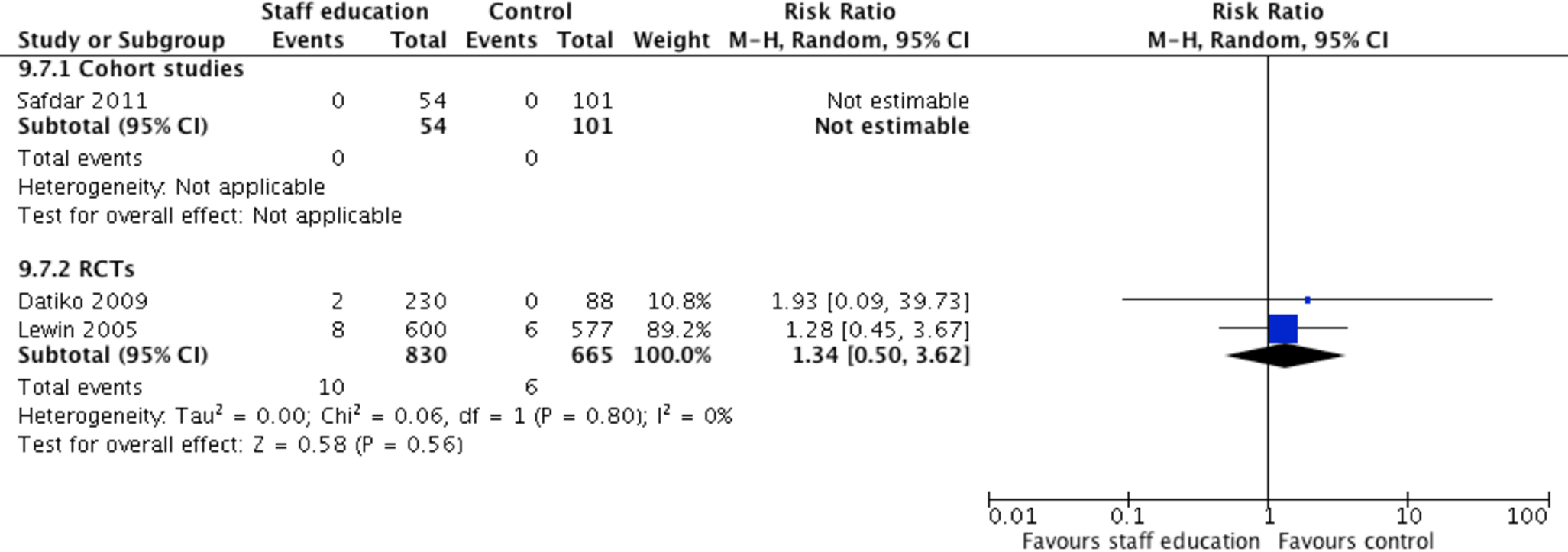

Supplement: S59 Fig — (TIF) [file pmed.1002595.s064.tif]

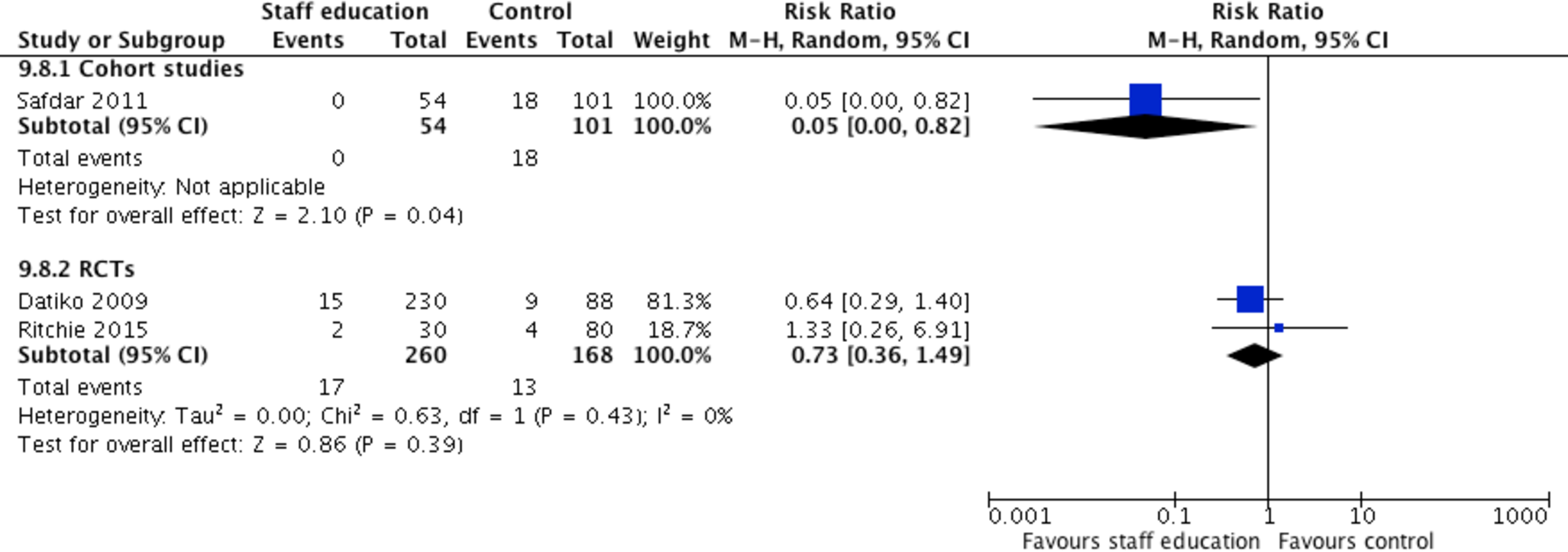

Supplement: S60 Fig — RCT, randomized controlled trial. (TIF) [file pmed.1002595.s065.tif]

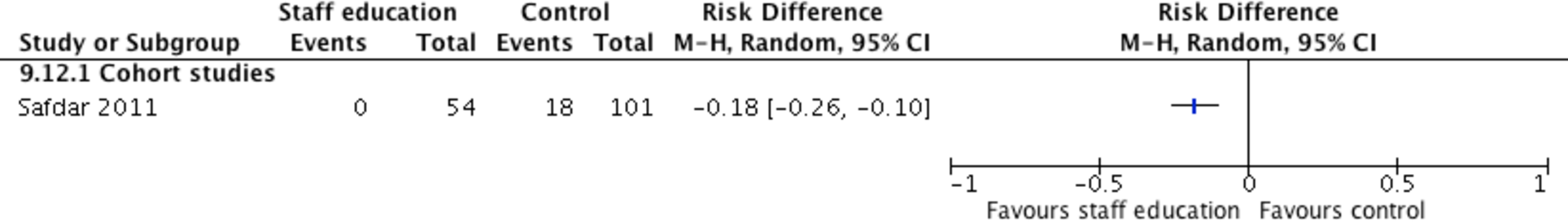

Supplement: S61 Fig — (TIF) [file pmed.1002595.s066.tif]

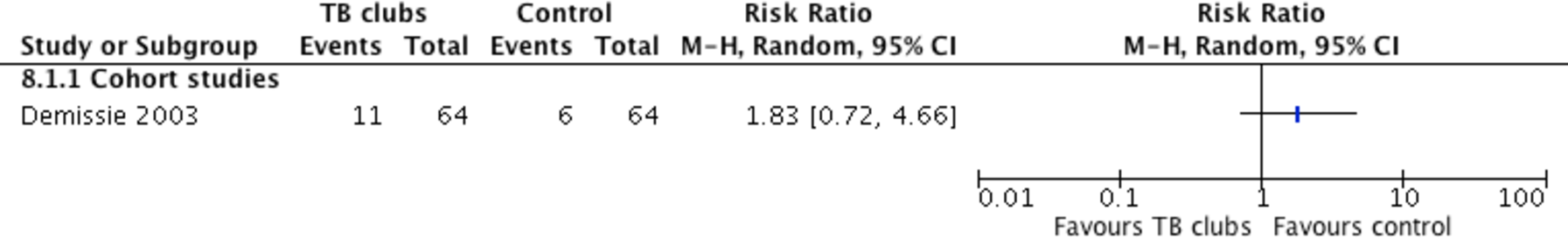

Supplement: S62 Fig — (TIF) [file pmed.1002595.s067.tif]

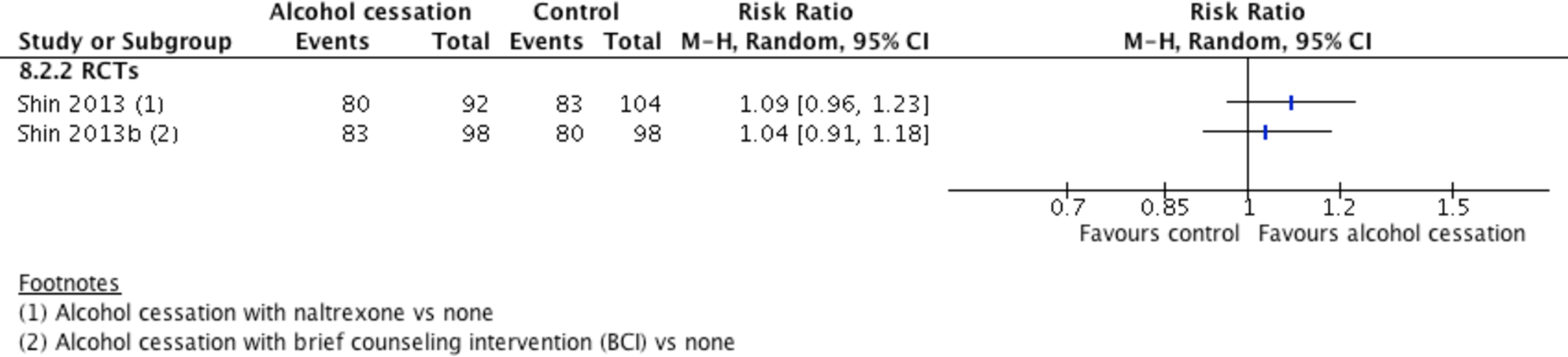

Supplement: S63 Fig — (TIF) [file pmed.1002595.s068.tif]

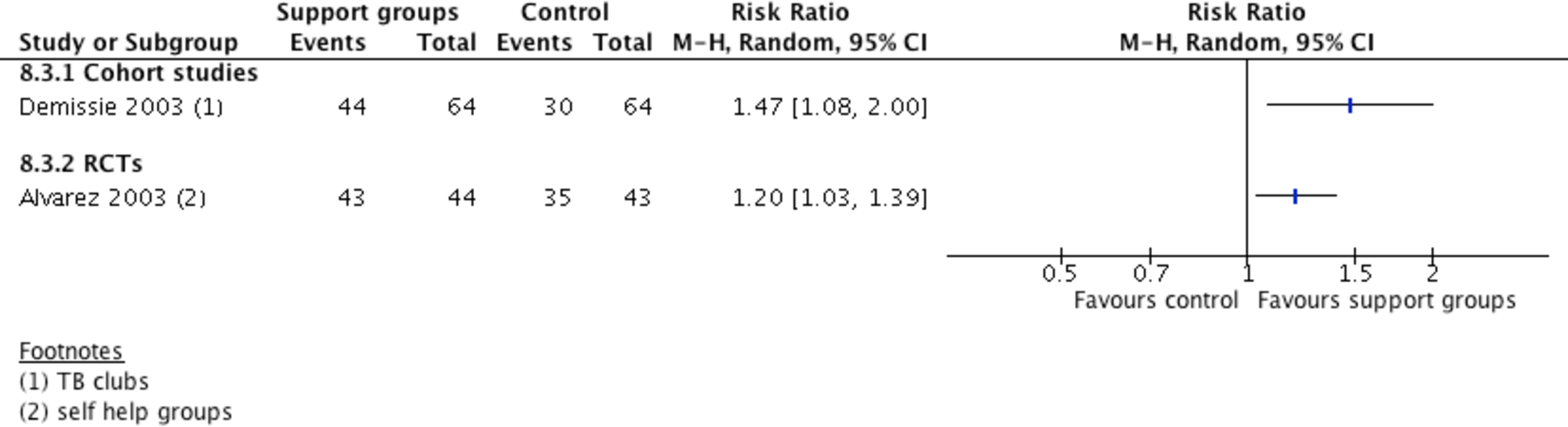

Supplement: S64 Fig — (TIF) [file pmed.1002595.s069.tif]

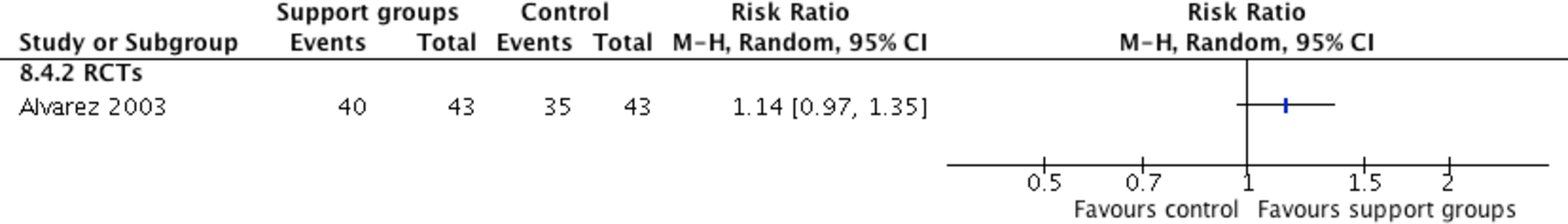

Supplement: S65 Fig — (TIF) [file pmed.1002595.s070.tif]

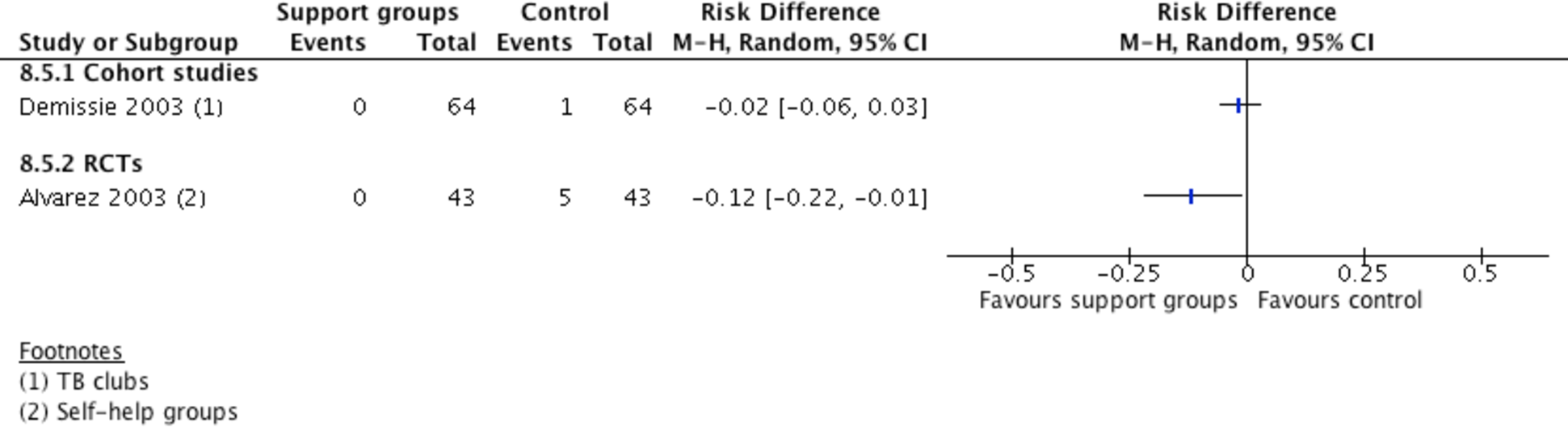

Supplement: S66 Fig — (TIF) [file pmed.1002595.s071.tif]

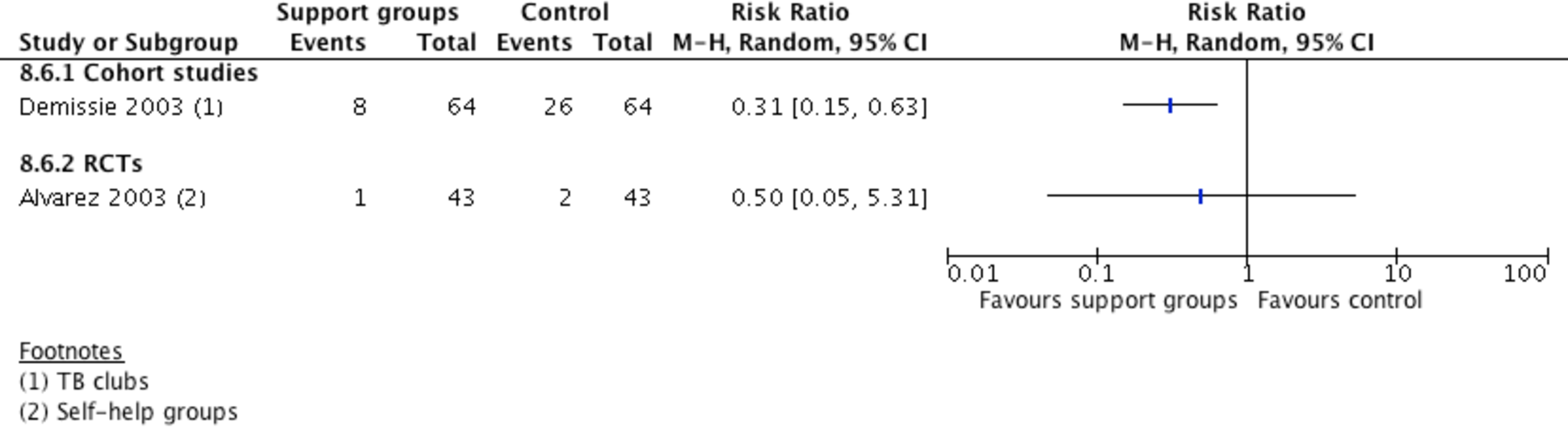

Supplement: S67 Fig — (TIF) [file pmed.1002595.s072.tif]

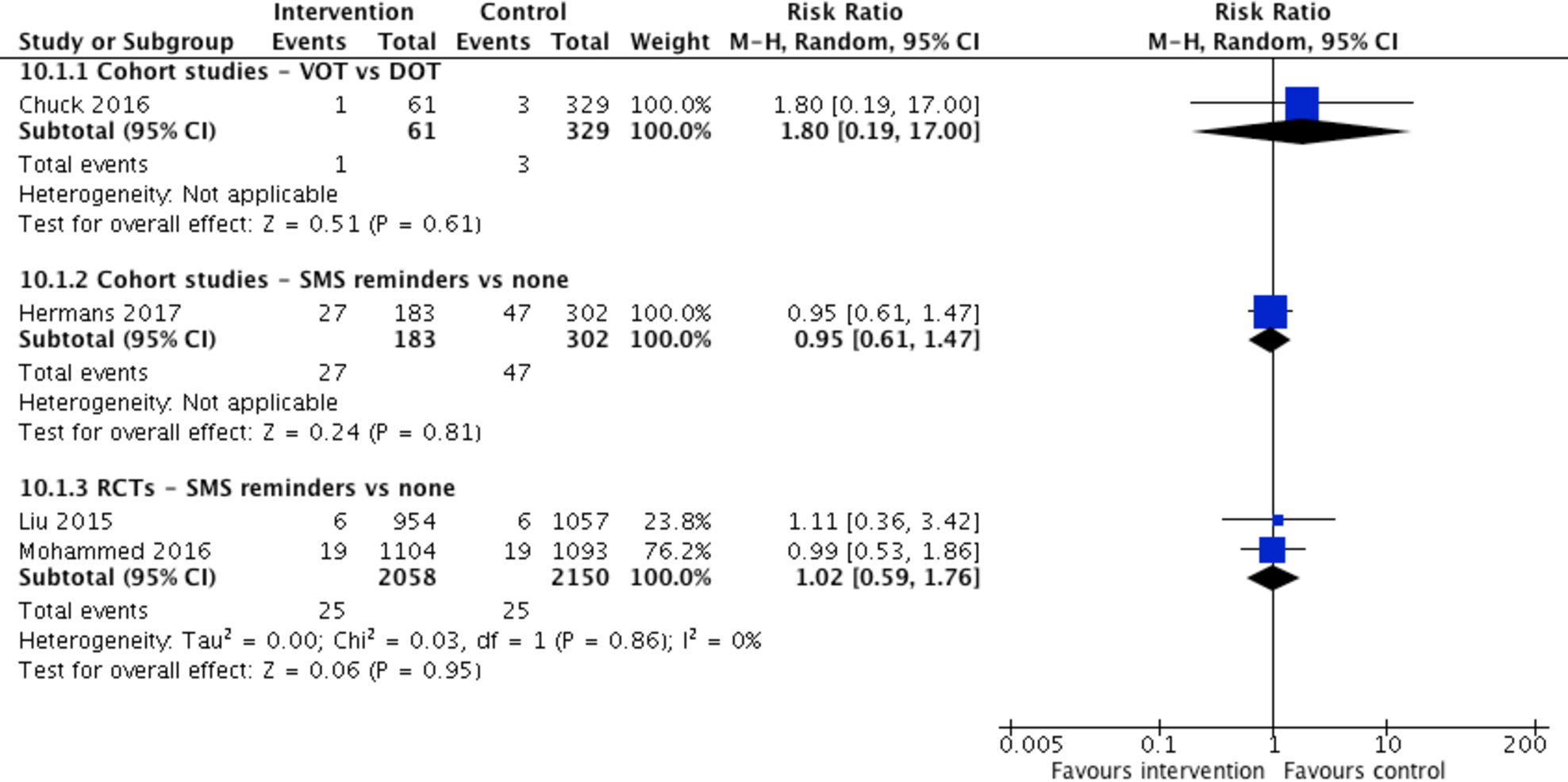

Supplement: S68 Fig — SMS, short message service; VOT, video-observed therapy. (TIF) [file pmed.1002595.s073.tif]

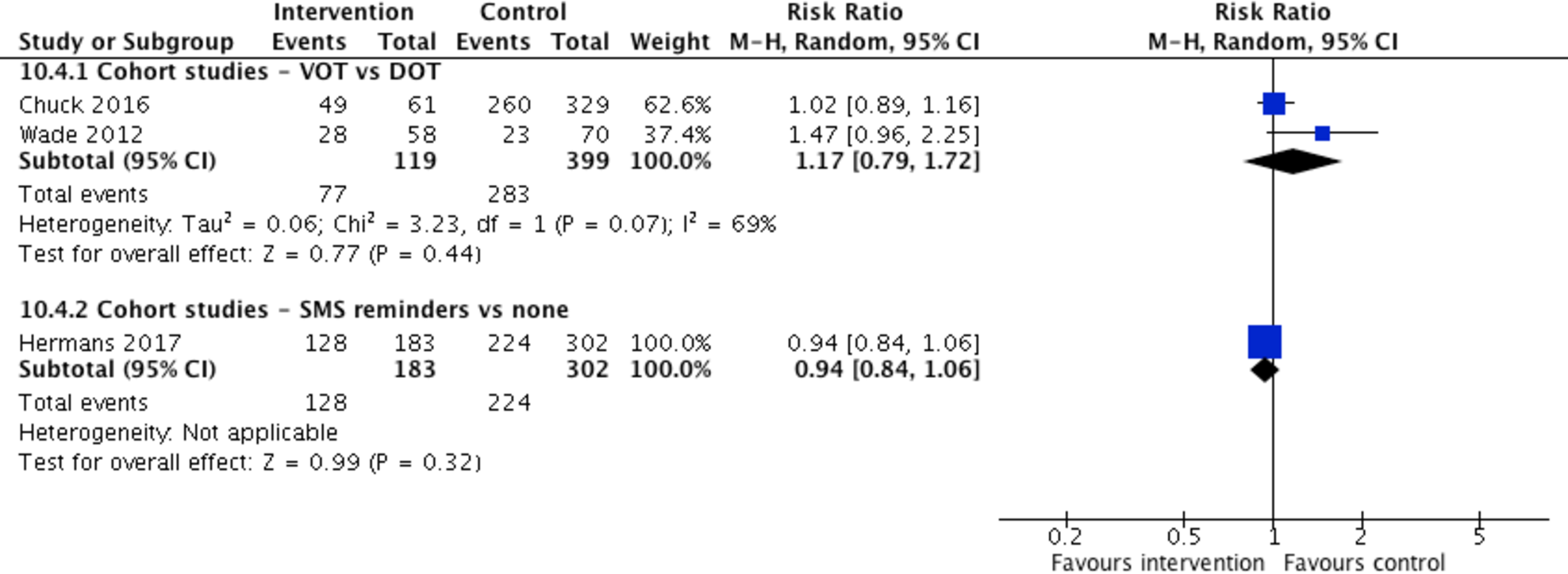

Supplement: S69 Fig — SMS, short message service; VOT, video-observed therapy. (TIF) [file pmed.1002595.s074.tif]

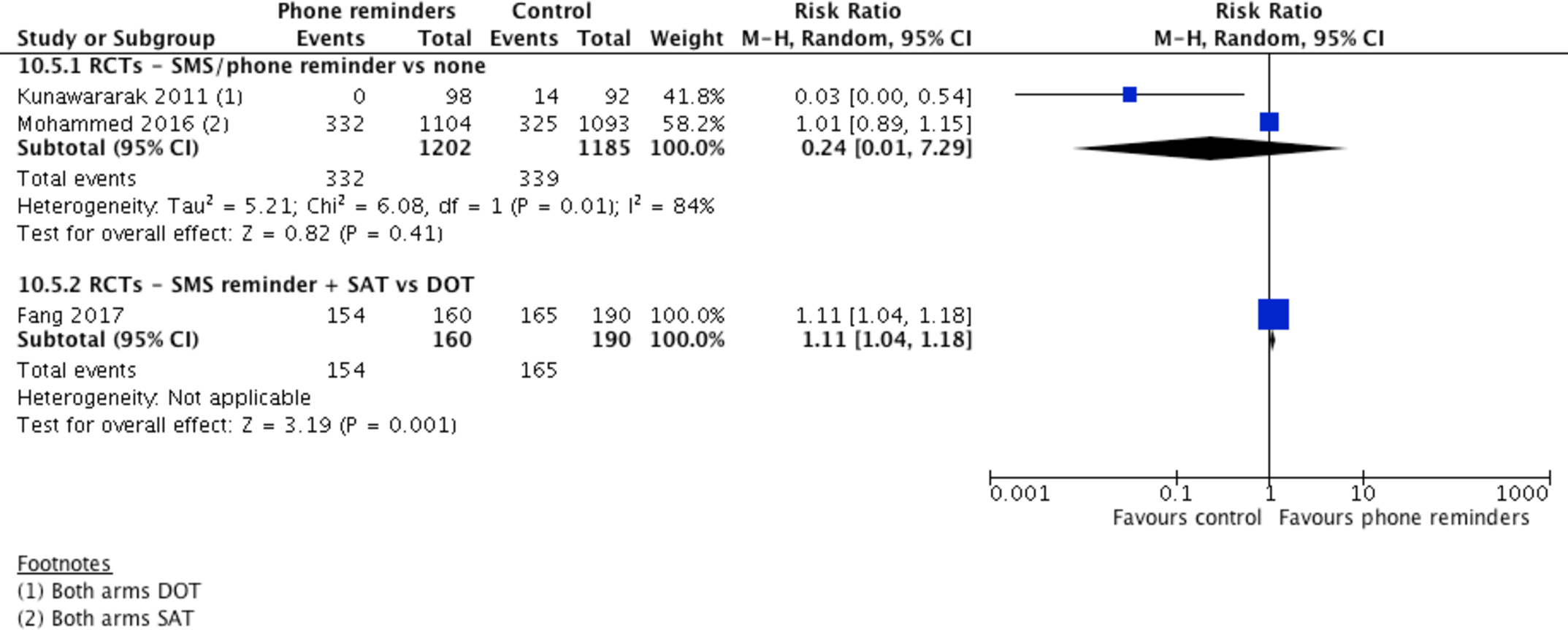

Supplement: S70 Fig — RCT, randomized controlled trial; SMS, short message service. (TIF) [file pmed.1002595.s075.tif]

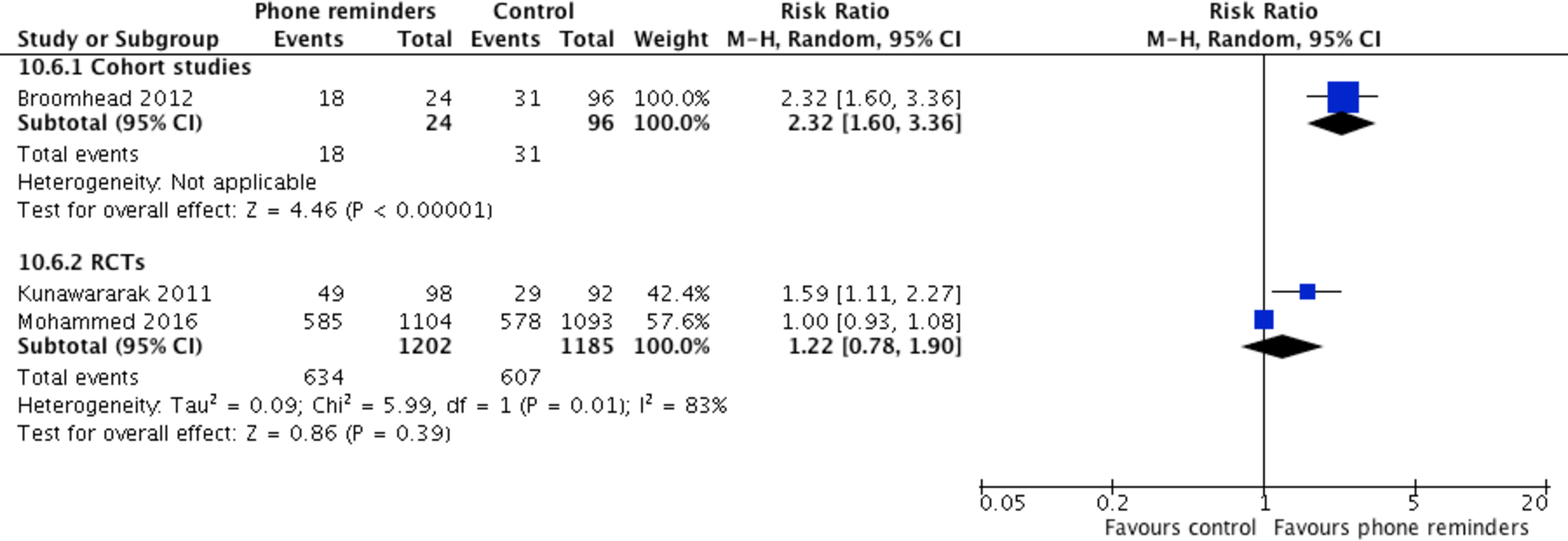

Supplement: S71 Fig — SMS, short message service. (TIF) [file pmed.1002595.s076.tif]

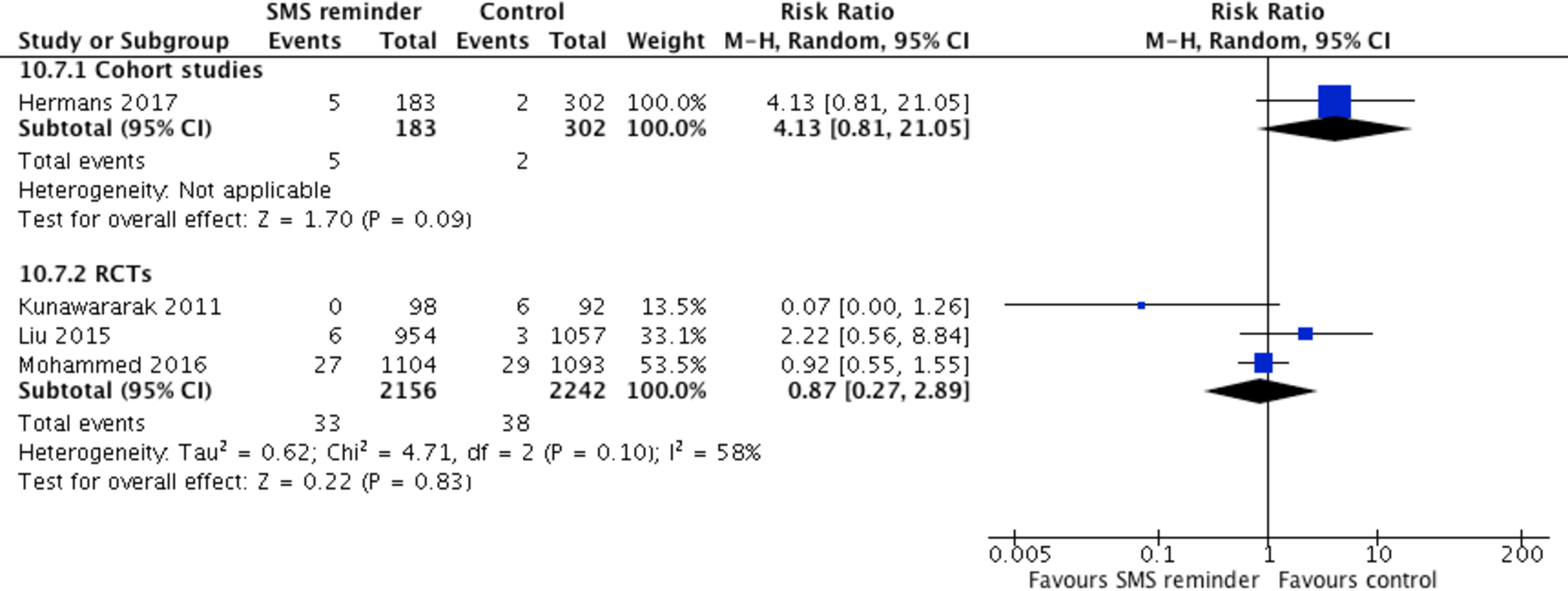

Supplement: S72 Fig — SMS, short message service. (TIF) [file pmed.1002595.s077.tif]

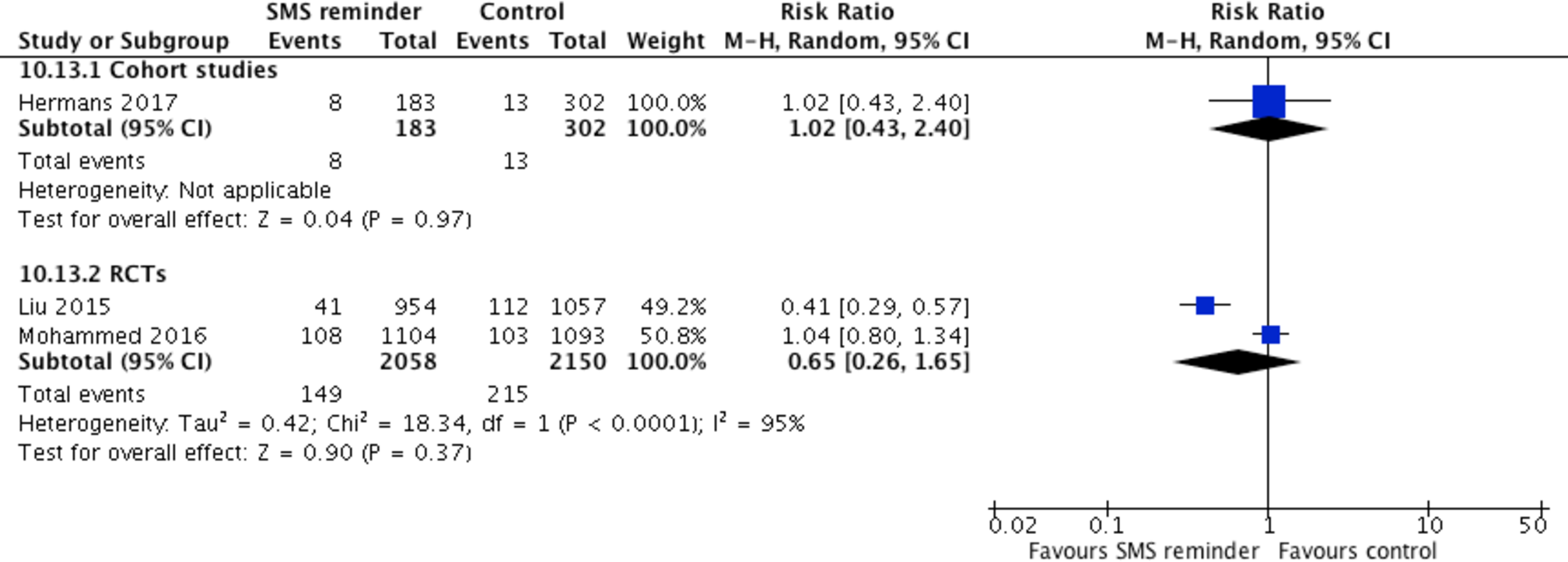

Supplement: S73 Fig — SMS, short message service. (TIF) [file pmed.1002595.s078.tif]

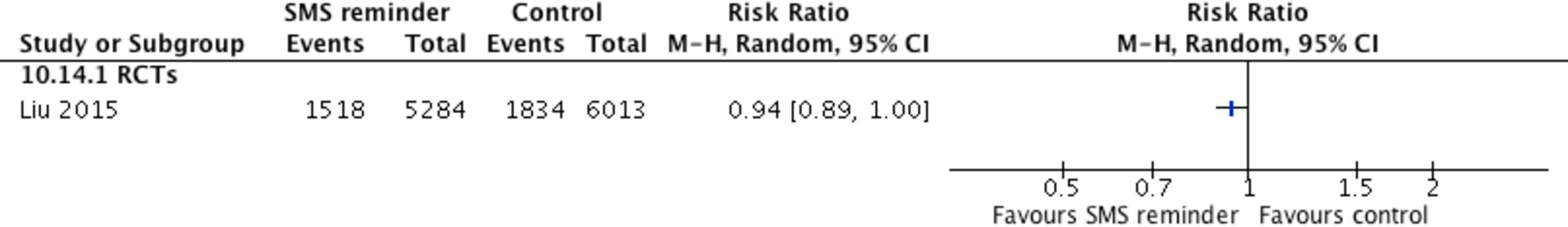

Supplement: S74 Fig — Poor adherence is defined as the percentage of patient-months in which at least 20% of doses were missed. SMS, short message service. (TIF) [file pmed.1002595.s079.tif]

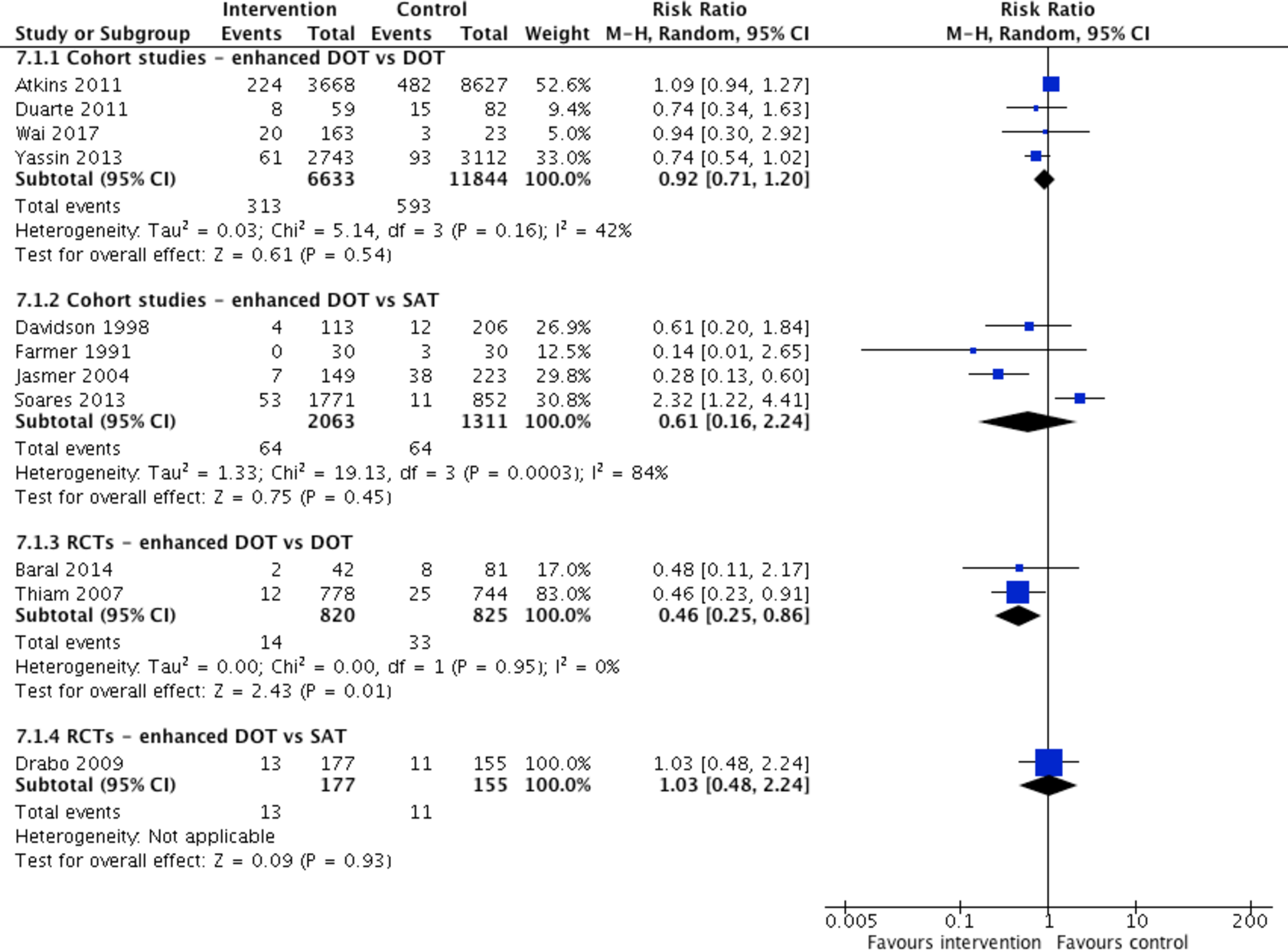

Supplement: S75 Fig — (TIF) [file pmed.1002595.s080.tif]

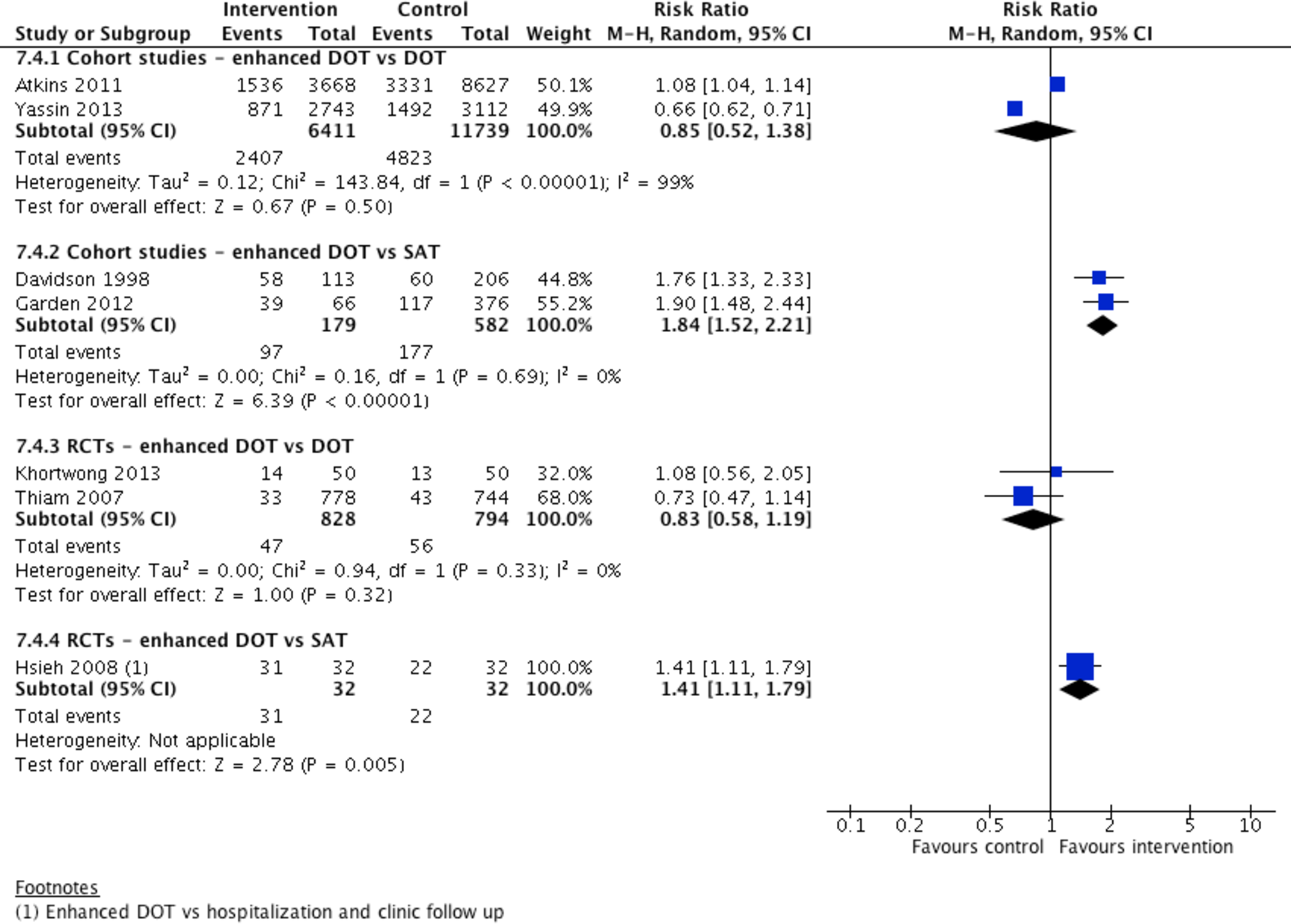

Supplement: S76 Fig — (TIF) [file pmed.1002595.s081.tif]

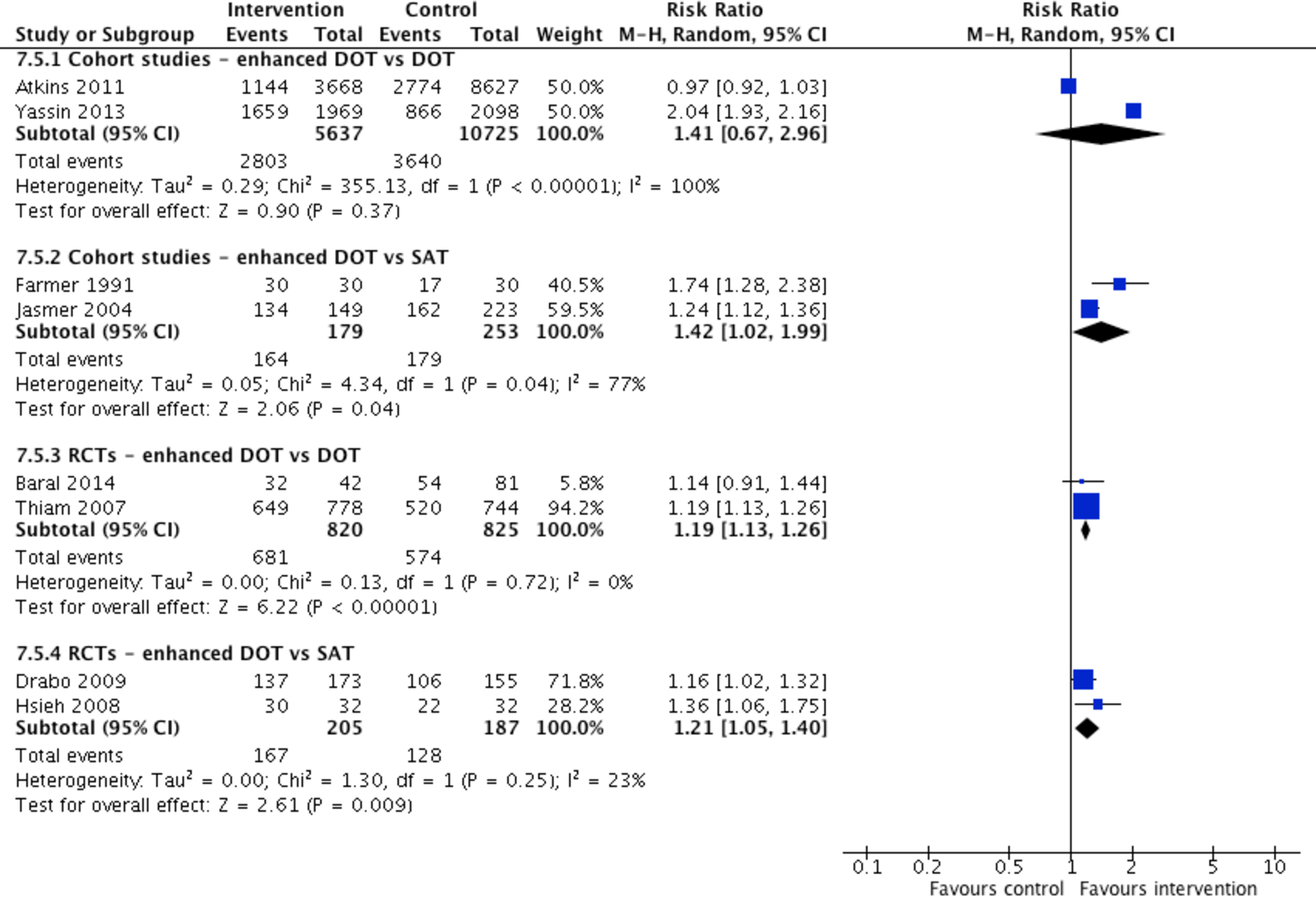

Supplement: S77 Fig — (TIF) [file pmed.1002595.s082.tif]

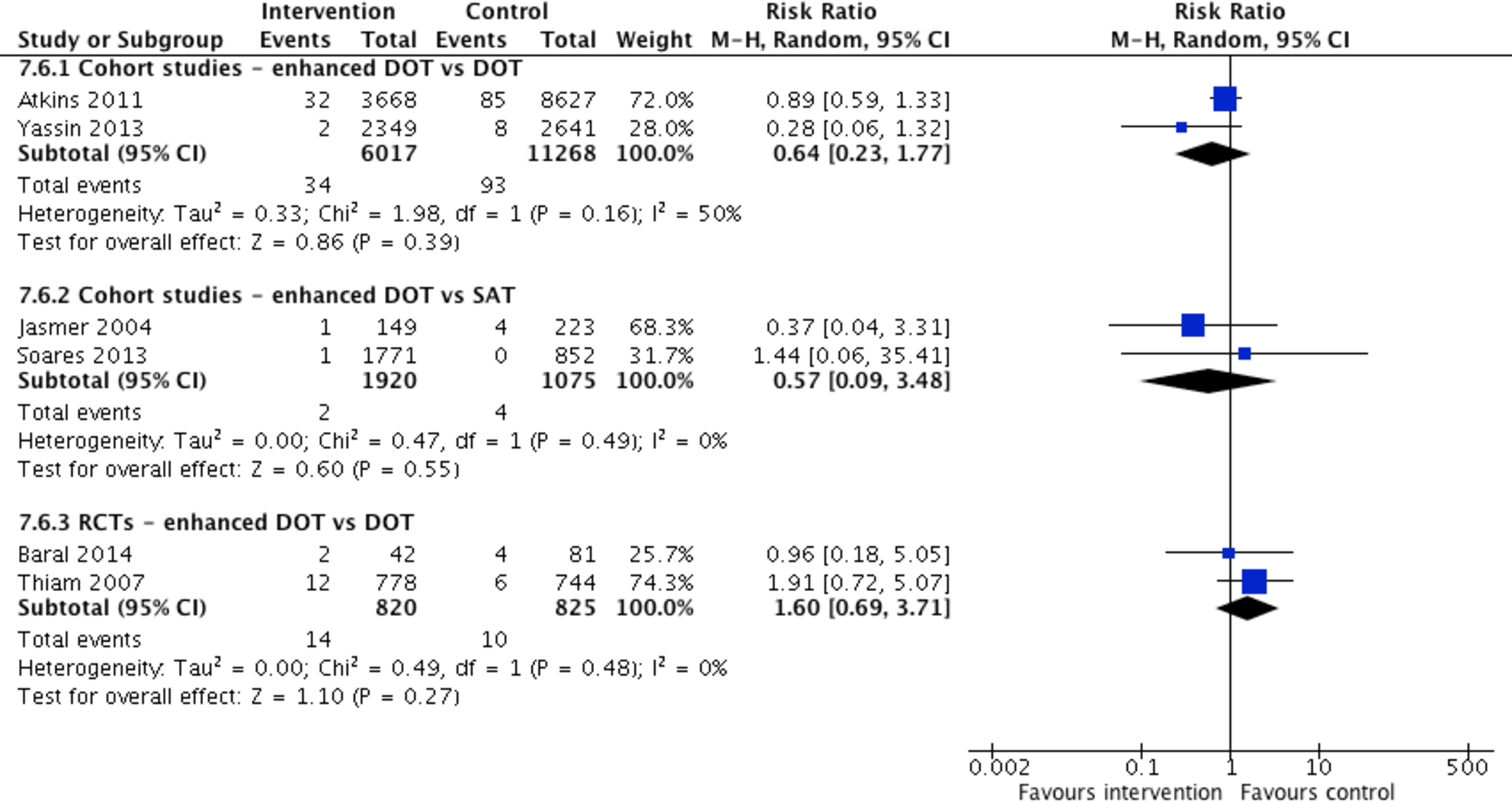

Supplement: S78 Fig — (TIF) [file pmed.1002595.s083.tif]

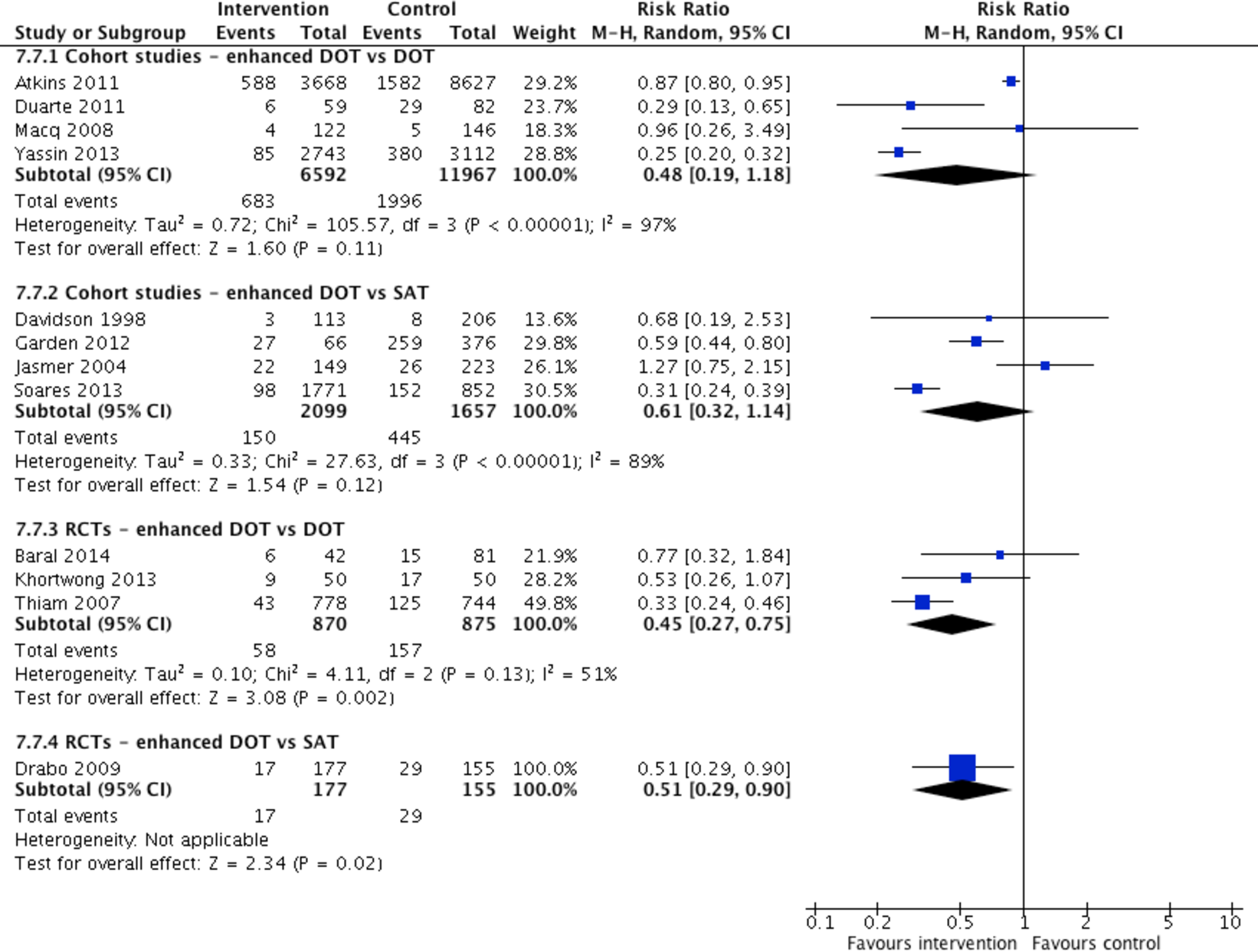

Supplement: S79 Fig — (TIF) [file pmed.1002595.s084.tif]

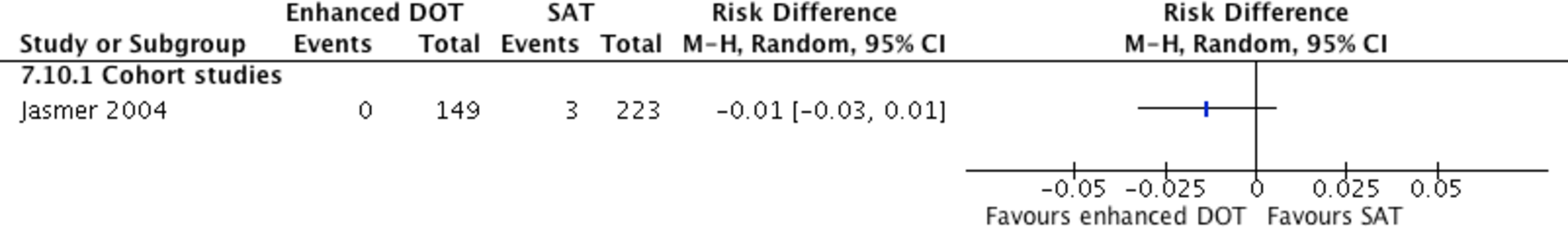

Supplement: S80 Fig — (TIF) [file pmed.1002595.s085.tif]

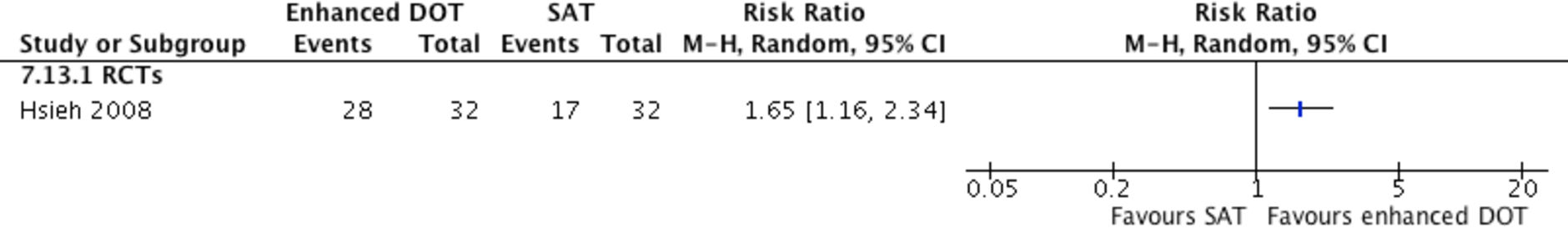

Supplement: S81 Fig — (TIF) [file pmed.1002595.s086.tif]

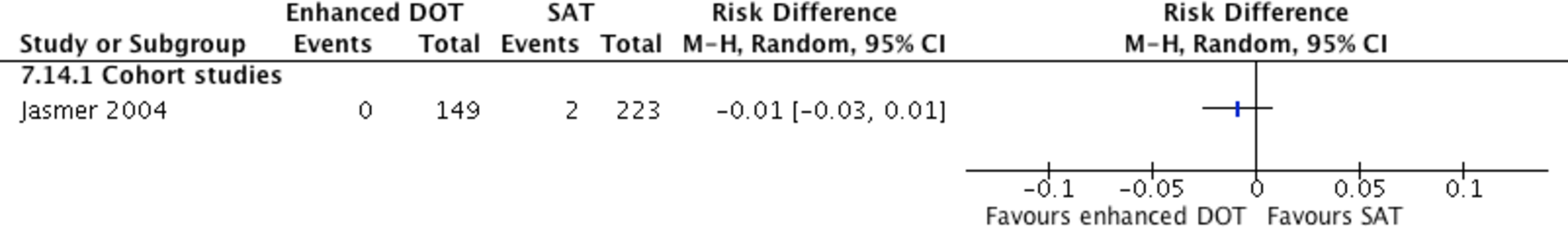

Supplement: S82 Fig — (TIF) [file pmed.1002595.s087.tif]
